# Review on the Accelerated and Low-Temperature Polymerization of Benzoxazine Resins: Addition Polymerizable Sustainable Polymers

**DOI:** 10.3390/polym13081260

**Published:** 2021-04-13

**Authors:** Bimlesh Lochab, Monisha Monisha, Nagarjuna Amarnath, Pratibha Sharma, Sourav Mukherjee, Hatsuo Ishida

**Affiliations:** 1 Materials Chemistry Laboratory, Department of Chemistry, School of Natural Sciences, Shiv Nadar University, Gautam Buddha Nagar, Uttar Pradesh 201314, India; mo447@snu.edu.in (M.M.); na639@snu.edu.in (N.A.); sm271@snu.edu.in (S.M.); 2 Department of Polymer Science and Engineering, Indian Institute of Technology, Hauz Khas, New Delhi 110016, India; pratibhasharma.venky@gmail.com; 3 Department of Macromolecular Science and Engineering, Case Western Reserve University, 10900 Euclid Avenue, Cleveland, OH 441067202, USA

**Keywords:** benzoxazine, polybenzoxazine, low-temperature polymerization

## Abstract

Due to their outstanding and versatile properties, polybenzoxazines have quickly occupied a great niche of applications. Developing the ability to polymerize benzoxazine resin at lower temperatures than the current capability is essential in taking advantage of these exceptional properties and remains to be most challenging subject in the field. The current review is classified into several parts to achieve this goal. In this review, fundamentals on the synthesis and evolution of structure, which led to classification of PBz in different generations, are discussed. Classifications of PBzs are defined depending on building block as well as how structure is evolved and property obtained. Progress on the utility of biobased feedstocks from various bio-/waste-mass is also discussed and compared, wherever possible. The second part of review discusses the probable polymerization mechanism proposed for the ring-opening reactions. This is complementary to the third section, where the effect of catalysts/initiators has on triggering polymerization at low temperature is discussed extensively. The role of additional functionalities in influencing the temperature of polymerization is also discussed. There has been a shift in paradigm beyond the lowering of ring-opening polymerization (ROP) temperature and other areas of interest, such as adaptation of molecular functionality with simultaneous improvement of properties.

## 1. Introduction

Benzoxazine (Bz) monomer is typically synthesized via Mannich-like condensation of a phenolic and a primary amine derivative in the presence of formalin or paraformaldehyde to form 1,3-benzoxazines (e.g., 3,4-dihydro-3-methyl-2H-1,3-benzoxazine). Bz contains a heterocyclic six-membered ring in an irregular chair conformation with oxygen and nitrogen atoms at the 1- and 3- positions, respectively. The monomer undergoes cationic ring-opening polymerization (ROP) to form polybenzoxazines (PBzs). Holly and Cope [1] first reported the synthesis of benzoxazine monomers, which was extended by Burke et al. [2,3] followed by Schreiber [4,5] and Higginbottom [6] to PBz resins. Ning and Ishida [7] reported the properties of PBzs in 1994. This led to an emergence of the PBz era. This class of polymers is an attractive alternative to many traditional thermosets, such as epoxies, phenol-formaldehyde, bismaleimides, cyanate ester resins, and even polyimides due to its excellent properties, rich design flexibility at the molecular level and competitive cost.

Since the discovery of PBzs, a large number of structural modifications have been considered to advance their applications. Due to their very complex structure and IUPAC names, the benzoxazine community has come up with an abbreviated monomer nomenclature where phenolic source is abbreviated in upper case character(s) and amine is abbreviated with lower case character(s); these two abbreviated names are connected by a hyphen. The corresponding polymer is abbreviated as poly(abbreviated name of monomer), and the same will be followed in this review article [8].

Depending on the molecular structure and functionality present in the monomer, they can be conveniently categorized into four generations. First generation benzoxazine includes monomers containing only benzoxazine functionality with rather benign substituents.

Second generation monomers possess additional functionalities, which undergoes either self-polymerization or crosslinking, or aid ROP reaction. In this category, benzoxazines can undergo other polymerization mechanisms in addition to the basic oxazine ring-opening reaction. Third generation benzoxazines include structures where oxazine groups are present in the main-chain of polymer, in the side-chain and as terminal groups to be classified as the main-chain, side-chain, and telechelic benzoxazines, respectively. These polymeric precursors have higher molecular weight than the traditional monomeric-type benzoxazines. Due to their oligomeric and polymeric architectures, they exhibit thermoplastic polymer-like properties prior to the crosslinking reactions. Preparation of self-supporting film thus becomes possible using this class of oligomeric precursors. Fourth generation benzoxazines possess smart architectures which include functionalities that affect formation of more stable in situ structures; these impart a set of unbelievable extraordinary properties to PBz frameworks, extending their scope to unconventional applications, which are limited otherwise by ordinary benzoxazine architecture.

In general, PBzs emerged as a promising class of thermoset polymers exhibiting versatility in a wide range of applications including adhesives [9,10,11,12,13,14], flame resistant polymers [15,16,17,18], cathodic material in batteries [19,20,21,22,23,24], coatings [14,25,26,27,28], materials for aerospace applications [29], carbon dioxide adsorbent [30,31,32,33,34], detection of metal ions [35,36,37,38], 3D printing [39] and electronics [40,41]. Besides effective compatibilization with many polymers, they also offer notable properties such as good mechanical strength [42,43,44,45,46,47,48], high thermal stability [49,50,51,52,53,54,55,56,57], flame retardance [58,59,60], good chemical and water resistance [61,62,63], near-zero shrinkage during polymerization [42,64,65], low dielectric properties [66,67,68,69] and lower surface free energy than PTFE being fluorine free structures [70,71,72,73].

To mitigate environmental issues, designing and structural changes of monomers that allow degradation of thermoplastics is gaining importance. Likewise, thermosets polymers based on bio-origin feedstocks and incorporation of labile linkages are being considered by the research community to allow reprocessability, recycling, and to tackle degradation. Recyclable and reprocessable PBz based on dynamic sulfide linkages [74] and labile Si–O–Ph bonds [75] are reported. A reprocessable polybenzoxazine thermoset is also achieved using nature inspired catechol-Fe^3+^ coordination chemistry [76].

The purpose of this review is not to introduce benzoxazines using natural renewable resources or development of flame-retardant polymers without using toxic additives. These are the reasons for polybenzoxazines to be attractive class of polymers for the green and sustainable technologies and are well recognized [77,78]. However, in order for the benzoxazine resins to be effectively and widely used to make them green, sustainable technologies, there are a few areas that need to overcome. One of such obstacles is to polymerize benzoxazine resins at low temperatures. Upon successful understanding of the effective approaches, it becomes possible to potentially green and sustainable materials to be actually useful materials.

## 2. Classification of Benzoxazine Monomers

### 2.1. First Generation Benzoxazines

#### 2.1.1. General Benzoxazine Synthesis and Structure

**Figure 1:**
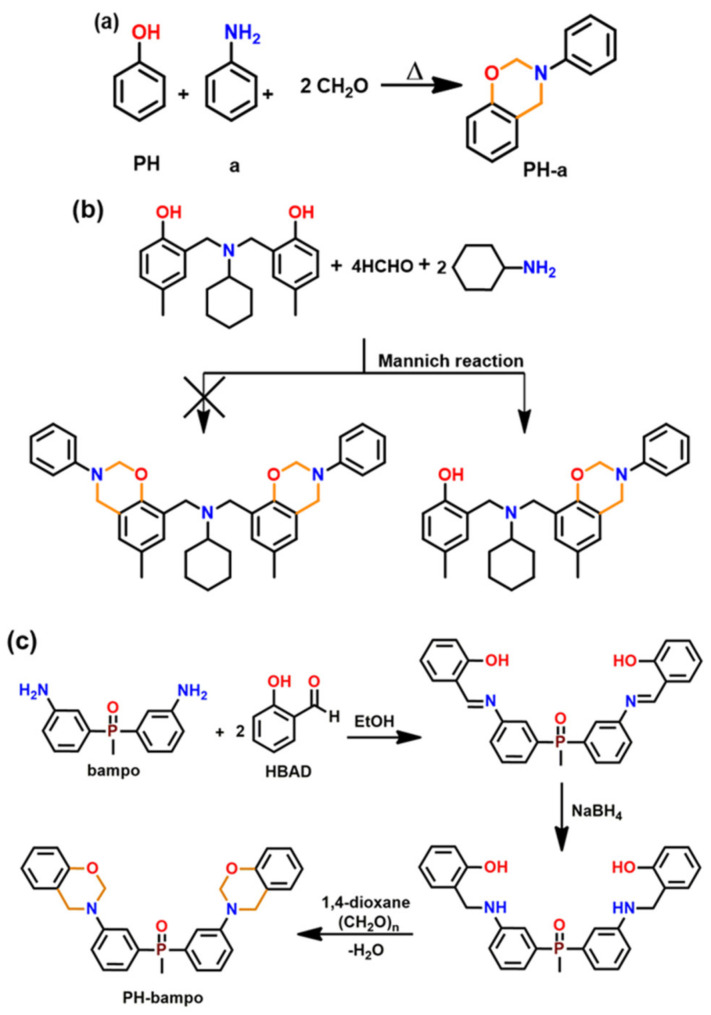
Synthesis of Bz monomers (**a**) one-step synthesis of representative benzoxazine monomer using phenol (PH), aniline (**a**) and formaldehyde, (**b**) asymmetric benzoxazine [80] and (**c**) multi-step synthetic approach [81].

Amongst other generations, they are the simplest structures and are formed by either one-step via the Mannich-like condensation or multi-step synthesis of an *o*-phenolaldehyde compound with any primary amine in solvent or solventless conditions [79]. The substituents present in the final benzoxazine monomers are rather inert groups, and thus, the polymerization mechanisms are the simplest among all the generations. A typical synthetic scheme for Bz monomer is shown in Figure 1.

It must be noted that not all phenolic-OH groups may condense with amine and formaldehyde to form oxazine ring. Interestingly, a dimeric phenol with an amine bridge led to the formation of an asymmetric mono-oxazine inevitably instead of expected bisbenzoxazine as in Figure 1b. This unusual and inequivalence of phenolic-OH reactivity is due to the existence of a strong intramolecular H-bonding (bond length ~0.18 nm) of one phenolic-OH with the >N- group, hampering its accessibility for ring-closure reaction [80].

Aside from highly reduced reactivity by intra-molecular hydrogen bond formation, lack of free *o*-position of phenolic structure can also prevent oxazine ring formation. Phenolic compounds, such as pyrogallol, 3-methoxycatechol, novolac, lignin, and tannin that contain some phenolic groups with no open *o*-position will remain as such rather than being involved in formation of oxazine. Such uncondensed OH groups will act as ring-opening initiators and/or catalysts and are known to shorten shelf life unless they are inactivated via intramolecular hydrogen bonding interactions or modified to chemical latent structures. In certain monomers, when some components interfere with the traditional mode, a three-step synthesis is found to be convenient in place of a one-step Mannich condensation reaction as shown in Figure 1c [81].

**Figure 2:**
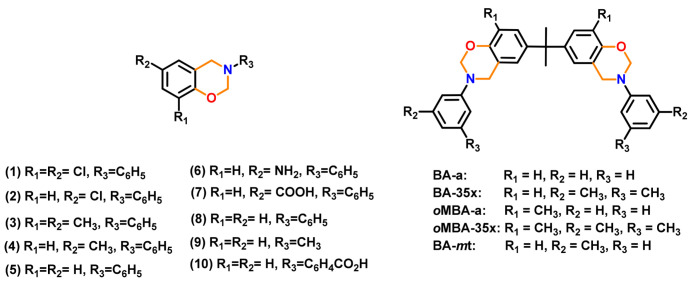
Representative mono- and bis-oxazine structures [82,83].

In general, the oxazine functionality number can be varied depending upon the nature of the starting materials and reaction conditions. The representative structures of mono- and bis-oxazine monomers based on phenols and amines in this first generation are shown in Figure 2.

**Figure 3:**
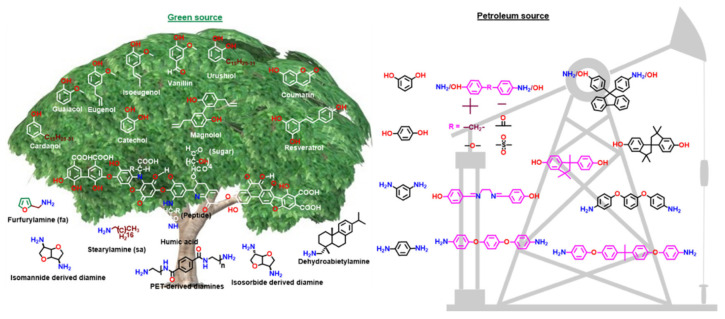
Representative bio- and petro-sourced phenolic and amine resources utilized to form Bz monomers.

To reduce the carbon foot print, exploration of non-petrochemical feedstocks that are obtained naturally or in bio-wastes is actively sought both as solvent or solvent-free reactions [84]. These include cardanol (C) [85,86,87,88,89,90]; guaiacol (G) [89,91,92]; eugenol [93,94]; isoeugenol [95]; vanillin (V) [96,97,98]; umbelliferone (U) [99]; catechol [100]; cinnamic, ferulic, coumaric [101,102] and phloretic acid [101,103,104]; magnolol [105]; resveratrol [106]; humic acid (coal origin) [107]; daidzein [108]; naringenin [109]; arbutin-linked phenol [110]; levulinic acid based diphenolic acid [111,112]; sesamol [113]; apigenin [84] and amines such as stearylamine [93], dopamine [114], furfuryl amine (fa) [95,115], aminolysed poly(ethylene terephthalate) [116] and isomannide diamine (ima) [89], as shown in Figure 3.

They exhibit the potential to compete with the existing petro-based high performance thermosets [117]. Lignin is a vast source of renewable phenols and used to produce biobased polymers. In general, besides as source for feedstocks replacement, greenness of the overall process should also be a criterion to judge the sustainability when compared with fossil-derived materials [118].

As proposed by Burke and Ishida [2,119], formation of benzoxazine monomer (PH-a) in acidic conditions preferentially proceeds via iminium ion intermediate instead of the sequential reaction of aniline and resultant intermediate with formaldehyde molecules, as supported by ionization mass spectrometry (ESI-MS) with infrared multiple photon dissociation (IRMPD) spectroscopy [120]. Small ring strain in oxazine ring due to the presence of two heteroatoms, N and O, demands a modestly high temperature ~140–240 °C to mediate ROP under catalyst-free conditions [121]. Besides thermal polymerization, monomers are also susceptible to electrochemical polymerization in acetonitrile/alkali aqueous media [122,123,124]. Experimentally, it was observed that the PBzs properties, especially thermal degradation characteristics, are improved by the incorporation of amines with the tendency to extend physical and chemical crosslinked networks [50,51,53,125,126,127,128,129]. Replacement of aniline with furfurylamine (fa) [130] and tritylaniline [131] showed predominance of amine-based polymer linkages.

#### 2.1.2. Mono-Oxazine Functional Monomers

The PBzs based on mono-oxazine monomers are mainly linear and may be lightly branched with the exception of PH-a, which is lightly crosslinked, whereas higher functionality monomers improve the crosslink density and, thereby, affect other properties of the polymer. The functionality of either phenol or primary amine is altered to increase the oxazine functionality in the monomer. The higher functionality ensures an infinite network growth, which is beneficial for thermosets. The intra-molecular hydrogen bond formation, propagation [80,132] and thermal dissociation of the mono-oxazine monomer compete with chain propagation reaction preventing growth of high-molecular weight linear structures. Another potential problem is the relatively easy evaporation of small, monofunctional monomer during polymerization process. Very recently, this termination of the chain propagation was attributed to the cyclic oligomer formation [133].

#### 2.1.3. Multi-Oxazine Functional Monomers

Problems associated with mono-oxazine functional monomers can be overcome by adopting multi-oxazine functional monomers. Ning and Ishida [134] synthesized first bis-benzoxazine using bisphenol-A (BA) with methylamine (ma) and aniline (a). Differential scanning calorimetry (DSC) kinetic studies revealed that polymerization proceeds via an autocatalytic mechanism [135]. The temperature and activation energy (*E*_a_) of polymerization of Bz monomers was found to increase under high pressure differential scanning calorimetry (HP DSC), and the resultant polymer showed a decrease in both *T*_g_ and thermal stability than the polymers polymerized at normal pressure [136].

Besides BA, other bisphenols with various units between two phenyl groups and their bridging group are used to affect the PBz structure and tailor their properties. Simultaneously, similar variations are explored in the amine component, too. Representative bis-phenols and bis-amines reported in Bz chemistry are shown in Figure 3.

**Figure 4:**
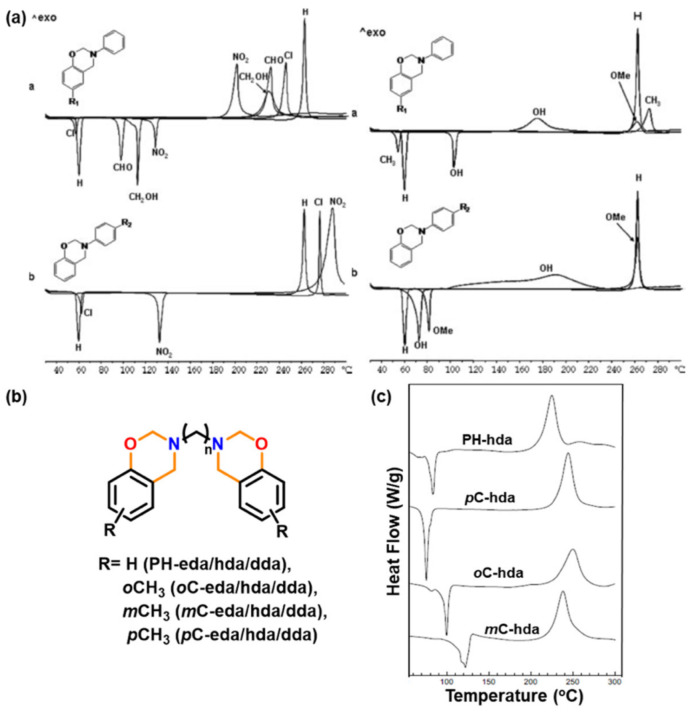
DSC thermograms of the (**a**) unsubstituted, *para*-, *ortho*- and *meta*-substituted mono-oxazine by varying substituents in phenol and aniline ring [138]. Copyright 2008. Reproduced with permission from Wiley Periodicals, Inc. (**b**) Bisbenzoxazine based on diaminohexane by varying methyl substituent in phenol ring (*o*-, *m*- and *p*-cresol) and (**c**) non-isothermal DSC thermograms of the PH-hda, *p*C-hda, *o*C-hda and *m*C-hda monomers based on diaminohexane (hda) [137]. Copyright 2009. Reproduced with permission from Elsevier Ltd., Amsterdam, The Netherlands.

A predominance of BA based benzoxazine monomers with a monofunctional amine is widely reported in literature. The bifunctionality in benzoxazine can also be affected in reverse fashion, by condensation of bis-amine with mono-phenol. Besides formation of new structures, such variation at molecular level imparts additional benefits of low viscosity during both synthesis and processing [137]. Regioselectivity, which is controlled by the position of the electron-donating and electron-withdrawing groups on the benzene ring, influences ROP temperature, *T*_p_ [138]. If an electron-withdrawing group is located *p*- to the phenolic-OH, this leads to a lower *T*_p_ due to resonance stabilization of the ring-opened structure; however, if located *p*- to the amine group, this has an opposite effect as illustrated in Figure 4a. PBzs based on *o/m/p*-cresol vs. phenol revealed methyl substitution at the *o*-position increases *T*_p_, suggesting a decrease in the reactivity of the monomer as shown in the structures of the monomers provided in Figure 4b and DSC traces in Figure 4c. The rate of polymerization is highest when R = H i.e., unsubstituted > *p*-(methyl) > *o*-(methyl) [137].

Of particular interest is the replacement of methyl to methoxy group at the *meta*-position with respect to the phenolic-OH led to a substantial reduction in *T*_p_ [139].

A structurally induced effect is studied in the benzoxazine monomers based on BA and 4,4’-methylenediphenol (BF) with aniline (a) and cyclohexylamine (cha) [140]. The BA based monomers produce a higher concentration of oligomers than those based on BF. This suggests that the nature of bridge between phenyl group affects the basicity of oxygen and nitrogen atom of the oxazine ring, which influences oxazine ring-opening and thus the subsequent formation of oligomers.

**Figure 5:**
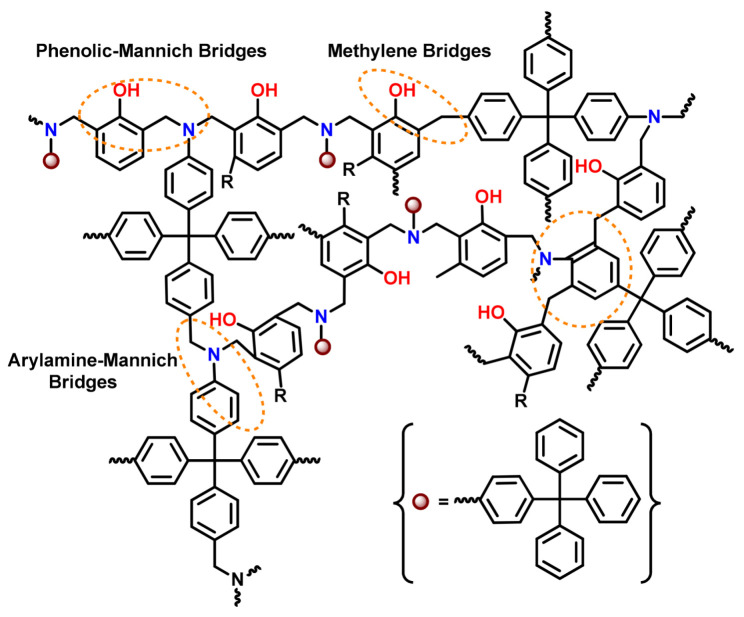
Polybenzoxazine framework with variable linkages in poly(C-ta) [131]. Copyright 2016. Reproduced with permission from Elsevier Ltd.

The nature of substituents, primarily electronic and steric effects, not only affects the temperature required for ROP but also governs the nature of linkages (mainly, *N*,*O*-acetal and/or Mannich structure) formed in the resultant polybenzoxazine network. For instance, Ishida et al. [141] reported the existence of non-Mannich type linkages, arylamine- and methylene-type, in addition to the traditional Mannich-bridges. Tritylaniline-based mono-oxazine sourced PBz framework revealed various such type of bonding as shown in Figure 5 [131]. ROP with methyl-substituted anilines revealed methyl substituents at the *o*-position of the arylamine ring sterically hinder the polymerization process. While the *m*-position facilitates polymerization at lower temperatures to generate bisphenolic methylene linkages, on the other hand, arylamine Mannich bridges that have reacted to the *p*-positions on arylamine ring appeared to be less thermally stable and may cleave during polymerization to yield methylene linkages [142]. The activation of the *p*-position on arylamine ring increases the extent of ring-opening during polymerization whereas the rings of the non-activated materials are much less likely to fragment and release the free amine.

**Figure 6:**
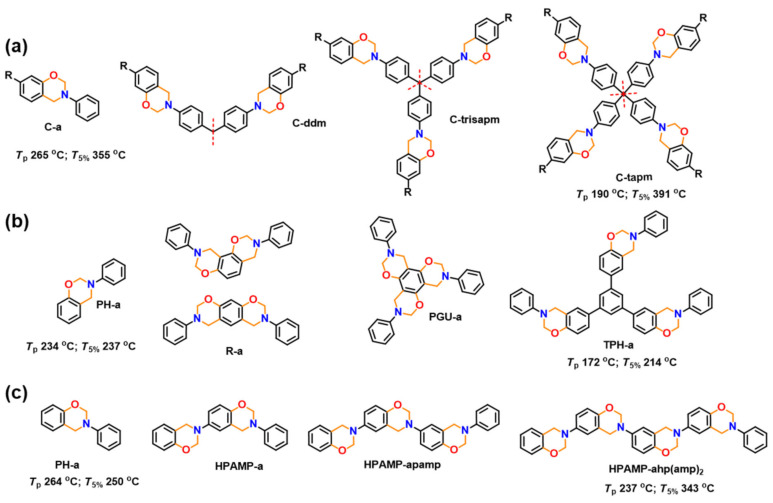
Higher functionality benzoxazine monomers [87,143,144,145,146,147].

A systematic enhancement of the number of oxazine functionality in symmetric fashion in the monomer from 1 to 4 (Figure 6a) led to a decrease in *T*_p_ from 265 to 190 °C as a result of close proximity of benzoxazine units [87]. The values of *T*_g_ (58–109 °C), thermal stability (*T*_5%_ 355–391 °C), char yield (13–37%), limiting oxygen index (LOI) (23–31) and storage modulus (3.6–66.5 MPa) improved significantly due to the growth of polymer network from one- to four-directions.

In a similar analogy, the existence of mono-, bis- and tris-oxazine functionality on the same benzene core revealed lowering in *T*_p_ with increase in functionality due to its specific condensed structure and the electronic effects of the aryl ethers at 1,3- and 1,3,5-positions as shown in Figure 6b [143]. On the contrary, an extreme lowering in thermal stability is observed at highest functionality which is due to the inability for PBz network growth as a result of non-availability of free *ortho*-position [144]. Interestingly, tris-oxazine with 1,3,5-triphenyl benzene (TPHB) core based Bz monomer (TPHB-a) showed a much higher *T*_p_ of 238 °C than phloroglucinol (PGU) based monomer PGU-a, suggesting close spatial proximity of oxazine rings is essential to lowering the ROP temperature [145]. Similar to the aforementioned example of an electron-donating methoxy group *para*- to the amine group, the reactivity of the resorcinol-based benzoxazine is highly elevated [146].

Sini and Endo [147] synthesized a series of di-, tri- and tetra- functional Bz monomers in ladder form via a multi-step synthetic procedure (Figure 6c) and found a reduction in *T*_p_ from 264 °C (mono) to 237 °C (tetra) and a simultaneous increase in char yield from 44% to 62% with an increase in the number of oxazine rings.

**Figure 7:**
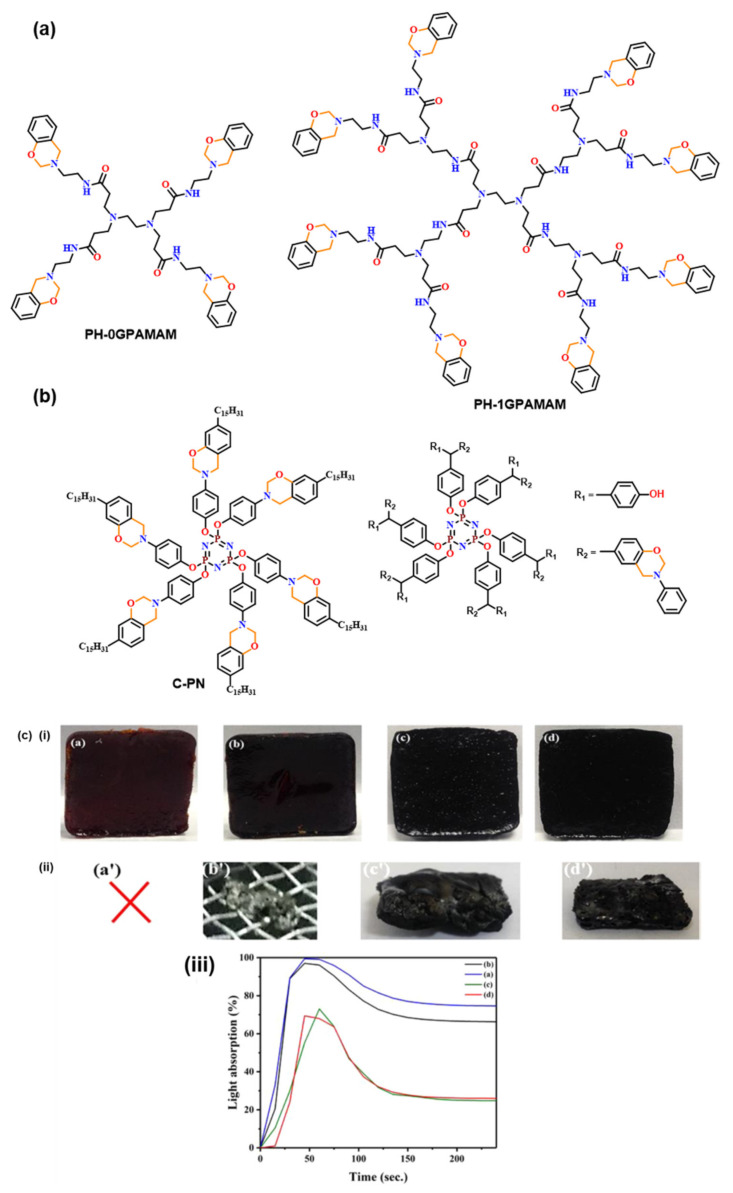
Higher order benzoxazine monomers with (**a**) 0th and 1st generation PAMAM dendritic [143], (**b**) phosphazene core [18,148] and (**c**) flame resistive analysis: digital images of cured samples [l × w × h: (25.0 ± 0.1) × (25.5 ± 0.1) × 3.0 mm] of poly (C-trisapm) and poly (C-PN) blends in different ratio (a) 100:0, (b) 90:10, (c) 20:80 and (d) 0:100 (i) before and (ii) after smoke density test; (iii) plot of light absorption by sensor with time during burning of the sample [18]. Copyright 2017. Reproduced with permission from American Chemical Society, Washington, DC, USA.

Interestingly, the dendrimers synthesized by Lu et al. [143], as shown in Figure 7a,b, exhibited a modest effect of increased number of dendritic arms on *T*_p_; however, the dendrimers in general showed lower *T*_p_’s than ordinary benzoxazines. A 4- and 8-functionality of benzoxazine monomers is obtained using different generation poly(amido amine) (PAMAM) dendritic amines. The 0th generation dendrimer with 4 oxazine ring showed *T*_p_ of 224 °C, whereas for the 1st generation dendrimer with 8 oxazine ring in the molecule, *T*_p_ = 220 °C. The spherical molecular architecture allowed a lowering of monomer viscosities. In general, the low viscosity of Bz monomer is advantageous as it allows solventless synthesis of other rigid monomers, which otherwise demands solvents for the synthesis [86]. Dendritic organic–inorganic hybrid cyclotriphosphazene (PN) based benzoxazine also revealed halogen-free flame resistivity as shown in Figure 7b,c [18,148].

Another example of dendrimer that has been reported by Lin et al. [149] showed little effect to the number of oxazine groups per molecule on the *T*_p_ at higher generations despite showing generally lower *T*_p_ than mono- or bis-oxazine benzoxazines. A facile one-pot Mannich condensation divergent approach was adopted with triphenylamine (tpa) as the core and phenol (tpa–2NH_2_–OH, as the AB_2_ branching group) to synthesize dendrimers with both bridged and surface oxazine surface groups. The 1st generation dendrimer with 9 oxazine rings per molecule showed the *T*_p_ of 231 °C, and the 2nd generation with 21 oxazine groups in the molecule, *T*_p_ = 235 °C, and the 3rd generation dendrimer with 45 oxazine groups per molecule, *T*_p_ = 235 °C. 

Besides dendritic, hyperbranched benzoxazine monomers based on trifunctional amine such as triphenyl amine (tpa) [150] and Jeffamine T-403 [151] with phenol and bisphenol A are also reported.

**Figure 8:**
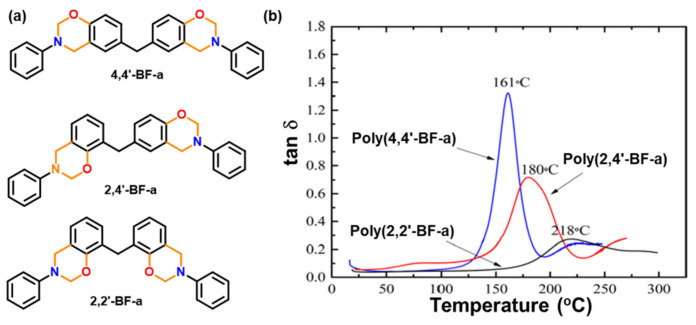
(**a**) Structures of isomeric BF-a monomers [152]. (**b**) Variation in tan δ of polybenzoxazines [152]. Copyright 2014. Reproduced with permission from American Chemical Society.

Aside from increasing the number of oxazine functionalities in the monomer, the isomeric effect of oxazine ring position also shows a profound effect on polymerization characteristics. The *T*_p_ decreased in the order of 4,4′-, 2,4′-, and 2,2′-isomers albeit showing only less than 10 °C difference between 4,4′- and 2,2′-isomers. A 2,2′-isomer of bisphenol F based polymer exhibited a much higher thermal stability and *T*_g_ than the 2,4′- and 4,4′- isomer as shown in Figure 8, which is attributed to better packing and high crosslink density for the 2,2′-based PBz framework. The superiority of the 2,2′-isomer over the 4,4′-isomer is highly unexpected, since all other PBz polymer literature studying the effect of isomerism reports otherwise [152].

Incorporation of a rigid aromatic structure in the PBz backbone such as fluorenyl and naphthol resulted in an improvement in properties, especially thermal stability, high char yield, LOI, and flame-retardance [153,154,155,156,157]. Incorporation of fluorene moiety in the Bz monomer imparted photoluminescence and UV stabilization characteristics to the PBz [158].

**Figure 9:**
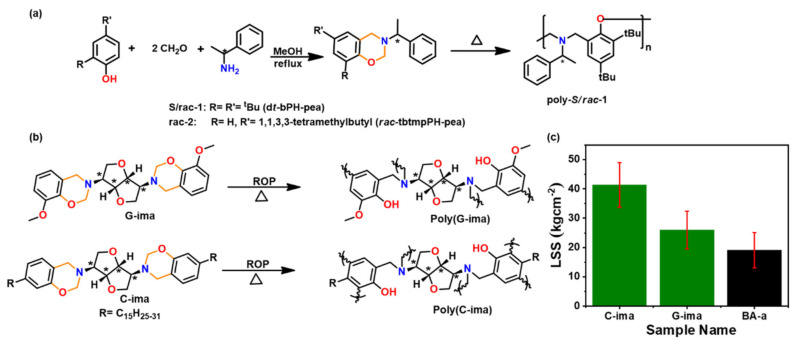
Synthesis and ROP of chiral Bz monomers (**a**) *S*- tbtmpPH-pea and *rac*-tbtmpPH-pea mono-oxazine [160], (**b**) G-ima and C-ima bis-oxazine [89] and (**c**) lap shear strength (LSS) values of C-ima, G-ima and BA-a benzoxazine based thermosets at room temperature [89]. Copyright 2019. Reproduced with permission from American Chemical Society.

Similarly to thermoplastics, properties of PBz framework are also affected by the chirality of the monomer [89,159]. The *S*-configuration origin PBz (*S*-tbtmpPH-pea) showed a slightly higher *T*_g_ (31 °C) than the racemic (*rac*-tbtmpPH-pea) (19 °C) due to stereoregular arrangement of units in the polymer backbone [160]. However, these values are much lower than the reported values for mono-oxazine based resins, due to blocked *o*- and *p*- positions with bulky *t*-butyl groups (Figure 9a). Recently Nagarjuna et al. [89] reported utility of chiral biobased isomannide sourced diamine (ima) to induce chirality in bis-oxazine monomers. This resulted in an unusual multiplet oxazine ^1^H NMR signals as compared to singlet signal, which are typically observed in traditional Bz monomers. The incorporation of rigid isomannide core resulted in fully biobased chiral PBz as illustrated in Figure 9b, a comparable *T*_g_ to their petro-based aromatic diamine counterparts and a 2-fold higher adhesive strength than poly(BA-a) (Figure 9c). 

### 2.2. Second Generation Benzoxazines

#### 2.2.1. Multiple Polymerization Mechanisms

In second-generation monomers, besides oxazine functionality, the monomer also contains additional reactive functionalities. Either these groups undergo polymerization by itself, or they assist polymerization of oxazine ring. The former category includes acetylene, allyl, epoxy, glycidyl, maleimide, methacrylate, nitrile, norbornene, oxazoline, phthalonitrile, propargyl, vinyl ester, cyanate ester, etc. The latter involves phenol, carboxyl, primary amine, methylol, ethylol, hydroxyl ethylether, etc.

**Figure 10:**
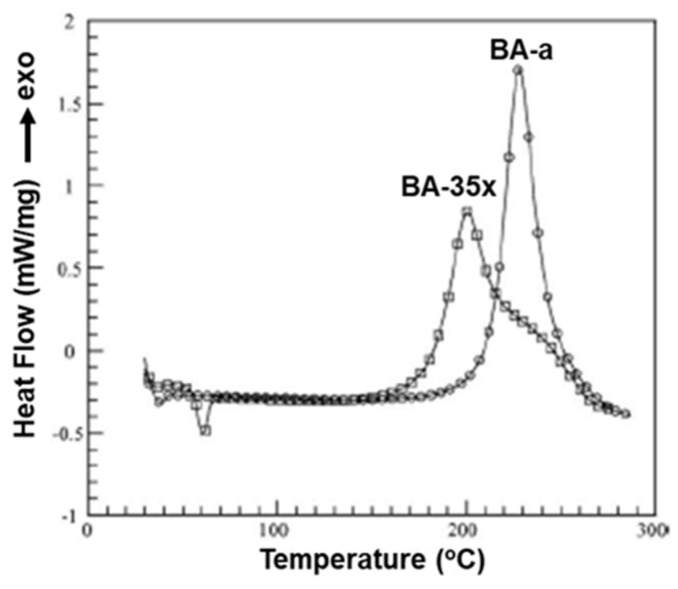
DSC thermograms of BA-a and (◦) BA-35x (▫) [165]. Copyright 2006. Reproduced with permission from Elsevier Ltd.

The position, nature, and number of oxazine rings affect the polymerization behavior and lead to various types of linkages in the resultant network. Figure 10 clearly shows a broad DSC exotherm in 3,5-xylidine (35x) based monomer, BA-35x, as compared to aniline based BA-a. This infers the existence of two polymerization reactions in BA-35x; the lower temperature exotherm is accounted for the ROP reaction, while the higher temperature shoulder corresponds to the side reactions such as formation of bisphenolic methylene linkages, arylamine Mannich bridge and methylene linked structures. Ishida and Sanders [142,161] supported existence of these linkages in BA-35x by FTIR spectroscopy. The structural variation of the polymer network is highly dependent on the nature of constituent phenol and amine and the additional functionalities present therein. BA-m and BA-a showed a significant effect on degree of polymerization and the crosslink density as investigated by ^13^C solid-state NMR [162]. A substantial influence on the degradation behavior of resultant polybenzoxazines is observed. Secondary amines are detected at temperatures <400 °C while phenols are detected at temperatures >400 °C in aliphatic amine based polybenzoxazines. In the case of poly(BA-a) and poly(22PP-a) below 600 °C, the degradation behavior of Mannich base cleavage under an oxidative environment is influenced significantly by the nature of constituent phenols below 600 °C [163].

Table 1 clearly shows that both introduction and position of methyl substituent on arylamine influences growth and degradation behaviors of PBz bis-benzoxazines prepared with various amines [164], possess the processing window as BA-35x < BA-mt < BA-a. The *T*_g_ were found to be consistent with the *T*_p_ of the polymerization exotherms, suggesting a significant variation in structure dependent reactivity to form crosslinking networks. The *T*_g_ of PBz based dimethyl substituted aniline (35x) is much higher at 243 °C than unsubstituted aniline of 168 °C [161,164]. Additionally, poly(BA-mt) and poly(BA-35x) display a delayed onset of thermal degradation (*T*_5%_) until around 350 °C, as compared to poly(BA-a). A regioselective control was found to improve the mechanical and thermal properties due to the occurrence of secondary crosslinking mechanism, leading to a predominance of thermally stable arylamine bridges [83,162,163].

Likewise in BA-35x, a very broad DSC polymerization exotherm comprised of several overlapped peaks was also observed in case of ethylenediamine (eda) based bis-benzoxazine monomers [137]. The two overlapped exotherm peaks of PH-eda are centered at temperatures that are atypically low for benzoxazines. This is attributed to the short ethylene linkage between the oxazine rings leading to steric hindrance or other structural interactions destabilizing the oxazine rings to affect polymerization at low temperatures [166]. A simultaneous occurrence of both polymerization and degradation is also reported in these structures. The highest temperature peak in these exotherms is ascribed to the degradation mechanism.

**Figure 11:**
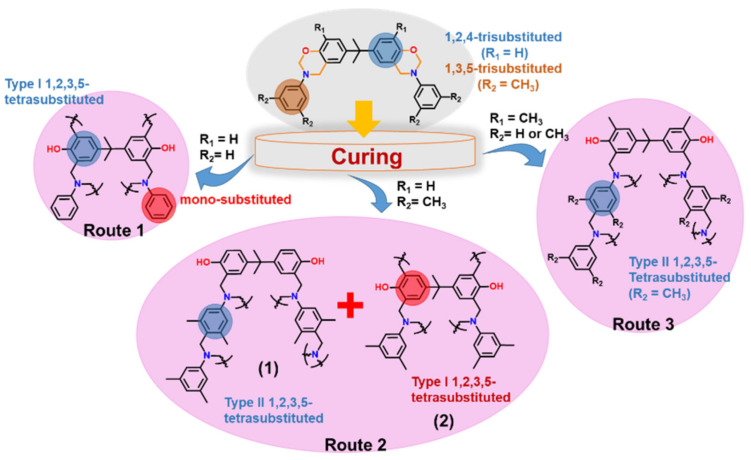
Possible polymerization routes for bisphenol-arylamine-based benzoxazines [83]. Copyright 2018. Reproduced with permission from Elsevier B. V.

A significant influence of methyl-substituent in Bz polymerization is also reported. In case of BA-a, *o*MBA-a and *o*MBA-35x, the polymer network growth proceeded by only one dominant auto-catalytic process with the existence of either phenol Mannich or arylamine Mannich bridge structure. On the other hand, BA-35x shows simultaneous occurrence of both crosslinking reactions. The nature and presence of methyl substituent in Bz monomer profoundly controls the type of linkages in polymer structure, as illustrated in Figure 11 [83,165].

**Figure 12:**
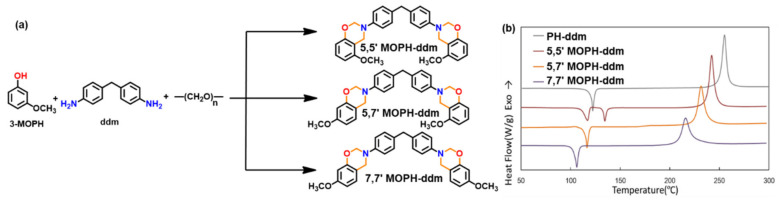
(**a**) Synthesis of 3-methoxyphenol and ddm based benzoxazine isomers and (**b**) DSC thermograms of MOPH-ddm isomers showing only well-defined single exotherm for each isomer [139]. Copyright 2020. Reproduced with permission from Royal Society of Chemistry, London, UK.

However, exhibiting multiple polymerization exotherms is not a common phenomenon in multi-functional benzoxazine monomers. Rather, multifunctional benzoxazines with possible multiple polymerization mechanisms often display a single exothermic peak. This seemingly a contradictory phenomenon was reported by Zhang et al. [167] and recently by Lyu et al. [139] Regio-isomers 5,5′-, 5,7′-, and 7,7′- based on 3-alkoxyphenol (methoxy, butoxy) and ddm, shown in Figure 12a, were purified to form single crystals to study the effect of benzoxazines with different reactivity within the same molecule. The DSC thermograms of individual isomers are shown in Figure 12b. Despite having 5- and 7-methoxy isomers in the same molecule, 5,7′-MOPH-ddm shows a symmetric and rather sharp exothermic peak at 226 °C, and this phenomenon is explained using the rate of reaction theory. 

**Figure 13:**
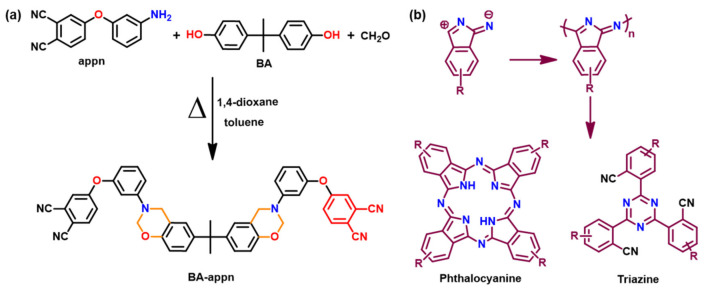
(**a**) Synthesis of phthalonitrile containing benzoxazine monomer [171] and (**b**) proposed nitrile groups based cyclic structures formed during polymerization [171].

Besides classical linkages formed by oxazine ROP, formation of other types of crosslinks such as ester linkages due to copolymerization, [168] triazine ester/isocyanurate [169] are also reported. Introduction of such additional linkages in PBz is advantageous and found to increase *T*_g_ of PBz. For instance, copolymerization of BA-a with 3,3′,4,4′-benzophenonetetracarboxylic dianhydride (BTDA) enhanced *T*_g_ to 263 °C. Nitrile based Bz monomers are prone to undergo phenolic group-mediated nucleophile triggering, which may form various nitrile-nitrile addition structures. These structures affect the crosslink density of the polymer network [170,171]. Figure 13a shows the synthesis of BA and 4-(3-aminophenoxy)phthalonitrile (appn) based phthalonitrile benzoxazine monomer. The nitrile groups form triazine and phthalocyanine structures, Figure 13b, in addition to oxazine ring-polymerization, thereby imparting a very high thermal stability (>400 °C) to poly(BA-appn). A simultaneous occurrence of oxazine ring-opening and triazine/phthalonitrile ring-forming reaction led to an improved mechanical performance, which is attributed to synergistic interactions between the polymer framework [172]. Curing kinetics of bisphthalonitrile Bz monomer was found to involve a free radical mechanism, where methine and methylene promote cyclization reactions of the cyano group [173].

**Figure 14:**
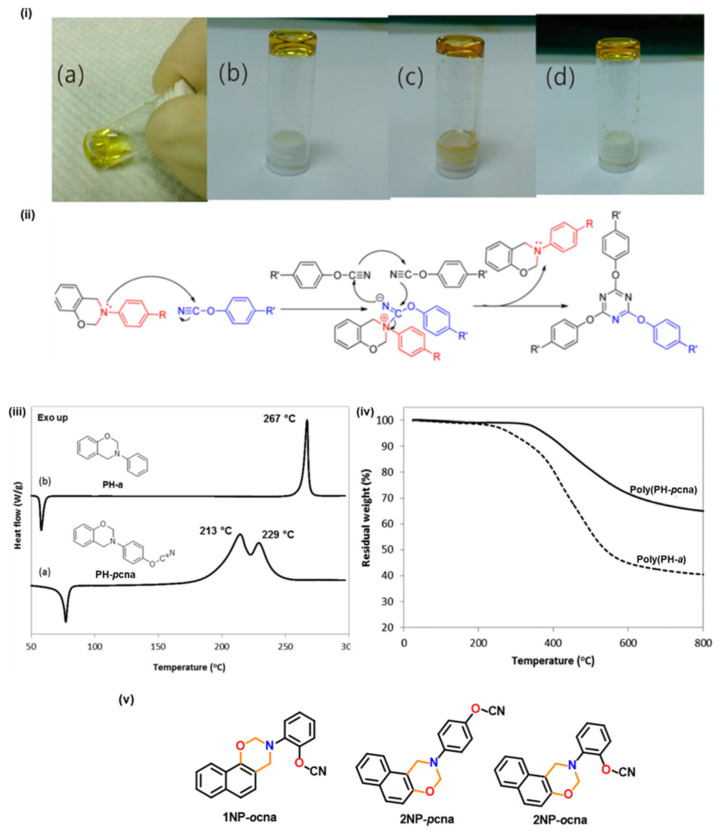
(**i**) Digital images of a 50 wt% methyl ethyl ketone solution of PH-oda/BADCY (Bisphenol A dicyanate ester) (1/1, mol/mol): (**a**) freshly prepared solution, and the thermally treated solution at (**b**) 30 °C for 24 h, (**c**) 50 °C for 4 h and (**d**) 100 °C for 2 h [178]. (**ii**) Proposed catalytic mechanism of benzoxazine to the trimerization of cyanate ester solutions [178]. Copyright 2015. Reproduced with permissions from American Chemical Society. (**iii**) DSC thermograms of monomers [181]. Copyright 2015. Reproduced with permissions from American Chemical Society. (**iv**) TGA thermogram of poly(PH-a) and poly(PH-*p*cna) polymerized at 220 °C/2 h [181]. Copyright 2015. Reproduced with permissions from American Chemical Society. (**v**) Cyanate ester functionalized benzoxazine (cna) [182].

Like nitrile, cyanate ester functionality also affects polymerization of benzoxazine functionality [174,175,176,177,178]. It was proposed that the phenolic hydroxyl group generated by benzoxazine ring-opening reaction reacts with the cyanate ester group to form iminocarbonate intermediate, which triggers cyclotrimerization of cyanate groups [174,175,179,180]. Further, it was proposed that the ring-opened oxazine rings insert into the triazine rings to form cyanurates and isocyanurates, which then probably further react with oxazine rings to form other structures [176]. Wang et al. [178] proposed the benzoxazine ring itself catalyzes both trimerization of cyanate ester and isomerization (cyanurate to isocyanurate) reactions upon heating. They showed the possibility of ROP for benzoxazine at 30 °C as evident from Figure 14i. This supported that the benzoxazine ring only induces the catalytic effect in trimerization of cyanate linkage as proposed in Figure 14ii. Ohashi et al. [181] proposed that the existence of cyanate ester functionality in the benzoxazine monomer affects its polymerization at a lower temperature than the benzoxazine/cyanate ester blends. Figure 14iii clearly supports the existence of multiple exotherms due to two different types of crosslinking processes in the DSC trace. They proposed the cyanate ester reaction promotes benzoxazine cationic ring-opening polymerization more efficiently when it is present in the benzoxazine structure itself. TGA thermogram, Figure 14iv, shows the improved thermal stability of PBz containing cyanate ester group. Besides cyanate ester crosslinking reactions, the high thermal stability is also attributed to the co-reaction of the resultant amine moiety to form additional crosslinks and thereby may prevent decomposition or evaporation of aniline component from PBz during heating. 

Despite expected higher thermal properties of PBzs based on naphthol, use of non-functional naphthoxazines is somewhat limited due to evaporation of the naphthoxazine units prior to polymerization [183,184]. Naphthoxazines functionalized with cyanate ester, Figure 14v, revealed minimization of such mass losses as indicated by multiple exotherm maxima, to support cyanate ester trimerization along with ROP of Bz ring. Additionally, incorporation of cyanate ester functionality not only lowered the polymerization temperature but also led to a relatively higher stability than their aniline counterparts as shown in Table 2 [182].

A simultaneous trimerization and oxazine ROP is observed only in cases of 1NP-*p*cna and 2NP-*p*cna while *o*-substituted cyanate esters, 1NP-*o*cna and 2NP-*o*cna, showed multiple exothermic peaks with a lower exothermic temperature than *p*-substituted cyanate ester functional naphthoxazines.

Other reactive functionalities, such as propargyl [185,186,187], epoxide [188,189], oxyalcohol [190] and amino [191] groups, are also incorporated in benzoxazine structures to impart additional crosslinks or other related benefits due to ease of structural control at the molecular level. Interestingly, aldehyde-containing benzoxazine, upon modification to a Schiff base, enables its applicability to sequester metals ions [192].

#### 2.2.2. Smart Benzoxazine Monomers

In addition to obvious reactivities of the additional substituent other than an oxazine ring as discussed in the previous section, there are certain structures or groups, which themselves do not possess ability to polymerize and yet can influence the rate or mechanism of oxazine polymerization. Such smart molecules can be quite useful as demonstrated in the following sections. 

**Figure 15:**
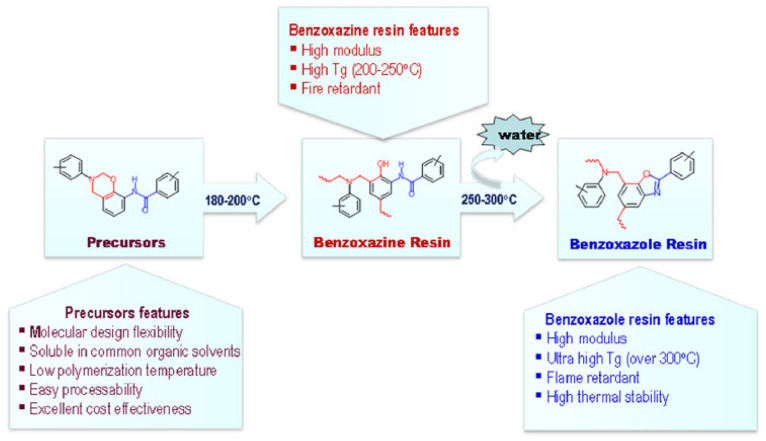
Smart features for easy synthesis of benzoxazole resin via *o*−amide functional benzoxazine monomer [193]. Copyright 2012. Reproduced with permission from American Chemical Society.

A unique class of *o*-amide functional Bzs was found to polymerize at much lower temperature than traditional Bz, without any help of initiator or catalyst. The presence of intramolecular H-bonding between an amide group and the adjacent oxazine ring acts as an internal trigger to stimulate ROP of oxazine ring at low temperature to form PBz with *o*-amide phenol functionalities. The *T*_p_ of Bz with *o*-amide found to be lower than *p*-amide isomer by 47 °C supporting the catalyzing effect induced by neighboring amide functionality via a 5-membered intramolecular H-bonding interaction. With further treatment at higher temperature (>ROP temperature) during post-curing or any event of catching fire, intramolecular cyclization between the neighboring hydroxyl and amide groups occurs to form oxazole rings with the elimination of water. Such interconversion of PBzs to polybenzoxazole imparts a better set of thermal properties due to formation of a more stable polymeric network as shown in Figure 15.

Regiochemistry of *o*- and *p*-amide substituents in Bz monomer containing pyridine ring affects the strength of intramolecular H-bonding interactions with the O in oxazine ring and N-H⋯N interaction with pyridine ring affecting polymerization characteristics and its conversion to PBO [194].

**Figure 16:**
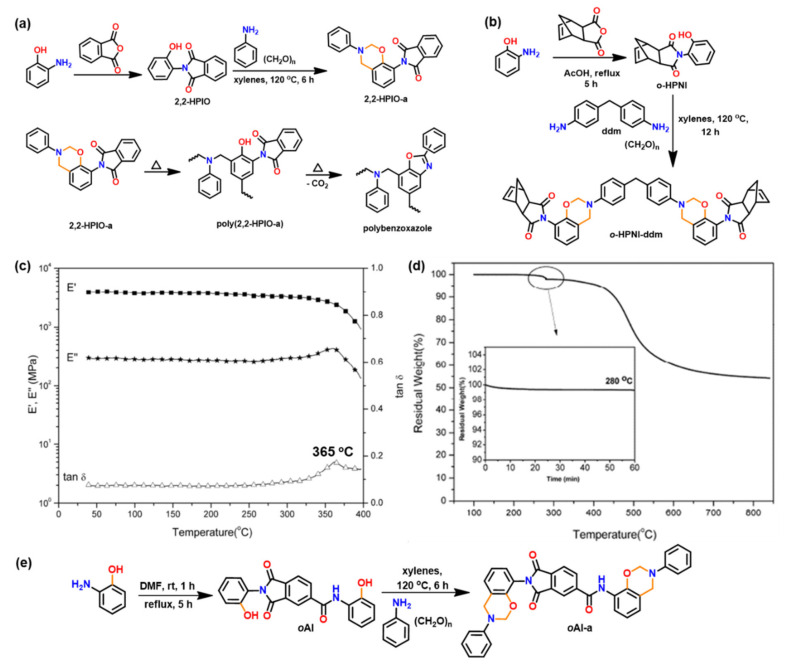
Synthesis of (**a**) *o*-imide [195], (**b**) *o*-norbornene functional imide Bz monomer [196] and (**c**) dynamic mechanical spectra of poly(*o*HPNI-ddm), upon further thermal treatment (280 °C, 1 h) [196]. (**d**) TGA of poly(*o*HPNI-ddm). At 280 °C, isothermal heating was applied for 1 h [196]. Copyright 2015. Reproduced with permission from Elsevier Ltd. (**e**) Synthesis of amide-co-imide functional benzoxazine monomer [197].

Similarly, *o*-imide functional Bz monomers are synthesized using the simplistic approach shown in Figure 16a via condensation reaction of 2-(2-hydroxyphenyl)isoindoline-1,3-dione (2,2-HPIO) with aniline (a) [195]. Benzoxazine resins with *o*-imide functionality revealed advantageous properties as compared with *p*-imide functional isomers. The crosslinked polybenzoxazine based on *o*-imide unit undergoes decarboxylation at high temperature to form a more thermally stable crosslinked polybenzoxazole (with *T*_5%_ = 505, *Y*_c_ = 73%). This method allows elimination of the traditionally used expensive raw materials and harmful solvents for polybenzoxazole synthesis.

Utilization of an *o*-norbornene functionalized, phenol sourced, *o*HPNI Bz monomer as illustrated in Figure 16b [196] led to minimization in degradation from the usual reverse Diels–Alder reaction. A very high *T*_g_ (365 °C) and high thermal stability (*T*_5%_ = 463 °C, *Y*_c_ = 61%) are reported, Figure 16c,d suggesting synergism provided by the co-existence of alkene and benzoxazine origin networks in the resultant polymer.

The polybenzoxazole thermosets based on the *ortho*-(amide-*co*-imide) functional benzoxazine monomers (BHPICA-a and BHPICA-ddm) are synthesized as shown in Figure 16e. As illustrated in Table 3, the attainment of very high *T*_g_ and thermal stability of these polymers upon heating at high temperature (400 °C) is supported by the formation of thermally stable linkages. This strategy demonstrates an easier and promising synthetic route of such polymers which can be used for high performance demanding applications [198].

The presence of *ortho*-nitrile group functionality in *ortho*-phthalimide-functionalized benzoxazine monomer revealed exceptionally high thermal stability (*T*_5%_ = 550 °C) and high char yield value (70%). This is accounted to by the formation of highly crosslinked network, stemming from the occurrence of multiple polymerization reactions, namely, oxazine ROP, cyano cyclotrimerization and benzoxazole ring formation [199]. Benzoxazine monomers with atropisomerism [200,201,202,203] account for a more interesting set of properties than traditional benzoxazine structures. The formation of highly cross-linked networks through multiple polymerization behaviors in single-component resins is advantageous in terms of ease of processability and high performance properties of *ortho*-maleimide functional benzoxazines with additional crosslinking such as cyano or acetylene functionalities [203].

**Figure 17:**
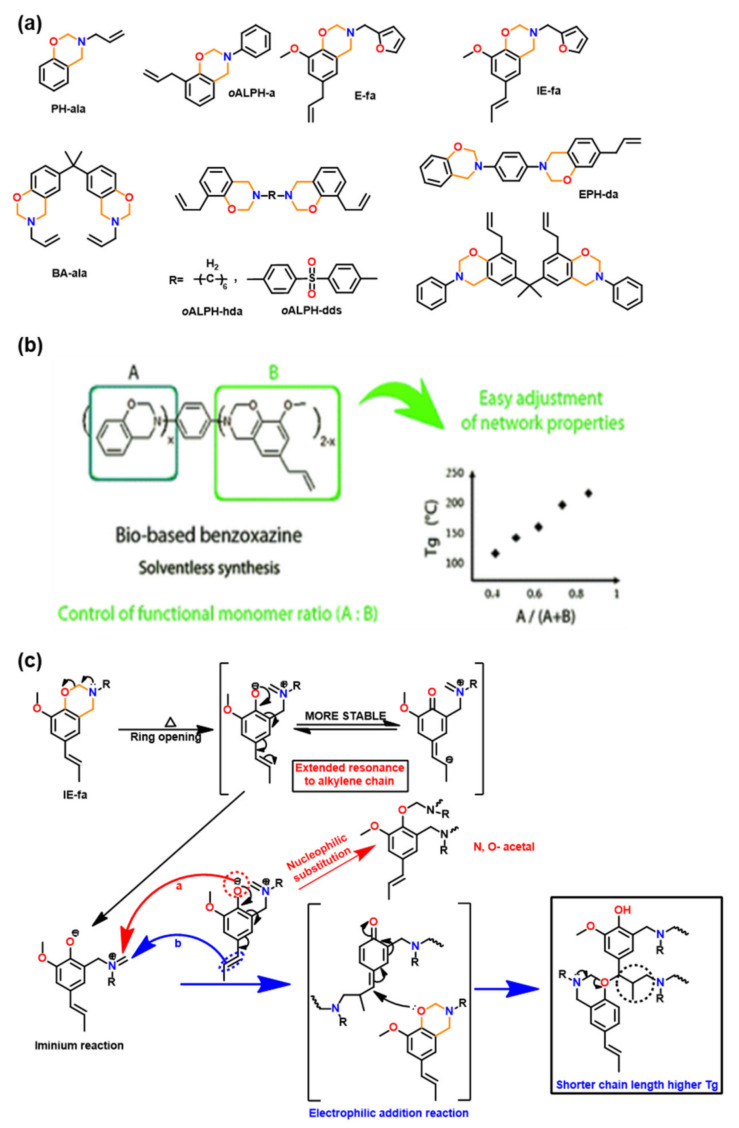
(**a**) Structures of allyl-functional benzoxazines [53,95,204,205,206]. (**b**) Strategy to affect *T*_g_ using hybrid bio-based monomer [94]. Copyright 2015. Reproduced with permission from Royal Society of Chemistry. (**c**) Probable mechanism of ring-opening polymerization in IE-fa Bz monomer [95].

A wide family of allyl functionalized benzoxazine monomers is shown in Figure 17a. Variation in position, number and hybrid structure of benzoxazine monomer is reported in the literature. Allyl groups were found to influence the polymerization behavior of Bz monomer [53,94,95,204,205,206]. The higher rate of polymerization of *N*-allyl substituted benzoxazine (PH-ala) as compared to *N*-(*n*-propyl) benzoxazine is attributed to the neighboring group participation of allyl group to assist oxazine ring-opening reaction [53]. PH-ala showed two exotherms with *T*_p_ at 207 and 260 °C that are associated with the thermal polymerization of the allyl group (attached to N) and formation of PBz, respectively. With an allyl group at the *o*-position, for *o*-allylphenol aniline (*o*ALPH-a) monomer, a single exotherm *T*_p_ at 263 °C is observed. Steric hindrance to polymerization is inferred from the observed low value of *T*_g_ and poor thermal stability of poly(*o*ALPH-a) [205]. Among difunctional allyl benzoxazines (*o*ALPH-hda and *o*ALPH-dds), sulfone bridged PBz showed relatively high thermal stability which is due to the significant difference in polarity of aliphatic and sulfoxide linkages [206]. The presence of inherent allyl groups in naturally occurring phenol, eugenol (E), was also explored for PBz. However, due to blocked *o*- and *p*-positions, it revealed limited polymerizability. This can be overcome by synthesizing hybrid bis-Bz monomer using phenol and eugenol. It was found that a variation in phenol to eugenol ratio in polymer allowed tunability in *T*_g_ (Figure 17b) along with the renewable content [94]. Isoeugenol (IE) is a naturally occurring structural isomer to eugenol, which has a propenyl instead of allyl group at the *p*-position. This variation in the position of double bond in phenol made a lower *T*_p_ value of IE-fa in comparison to E-fa. This suggested a prior involvement of double bonds to mediate ROP reaction of Bz monomer as the ring-opened phenolate ion is in conjugation with the alkenyl π bonds in IE-fa. A copolymerization of double bonds with oxazine is proposed as shown in Figure 17c [95]. Similarly, involvement of propenyl group in the crosslinking reactions was also noticed by Sha et al. [207].

**Figure 18:**
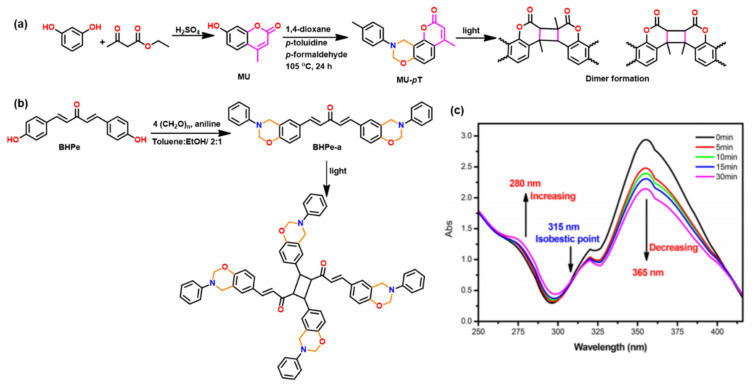
Synthesis of photoreactive unit: (**a**) coumarin [208] and (**b**) bis-benzylidene containing benzoxazine monomers and their light mediated dimerization [213]. (**c**) UV absorption spectra of BHPe-a in DMAc at a concentration of 0.8 mg/40 mL after irradiation at 365 nm for various periods of time [213]. Copyright 2017. Reproduced with permission from American Chemical Society.

Introduction of photoreactive functionalities, coumarin [208,209,210,211], chalcone [212], and bis-benzylidene as another smart feature in Bz monomer is reported. This created their capability for photodimerization [213,214] and exploration for self-healing applications. Kiskan and Yagci [209] utilized photoreactive coumarin functionality containing phenol, methyl substituted umbelliferone (MU), and *p*-toluidine (*p*t) based benzoxazine monomer, which upon irradiation at 300 nm underwent photodimerization via [2π + 2π] cycloaddition with alkenyl bonds as shown in Figure 18a. Like coumarin based Bz monomer, photosensitive bis-benzylidene linked Bz, BHPe-a (structure is shown in Figure 18b [213]) also undergoes [2π + 2π] cycloaddition to form cyclobutane ring-linked dimer. This allowed an increase in oxazine functionality and an extended monomer capability to undergo crosslinking reactions at the oxazine ring and double bonds. Figure 18c shows monitoring of dimerization reaction of monomer by UV–VIS. studies. A decrease in intensity at 365 nm due to π−π* absorption of a double bond of bis-benzylidene acetone, and increase in intensity at 280 nm due to δ−δ* absorption of newly formed cyclobutane ring with the irradiation time was observed.

**Figure 19:**
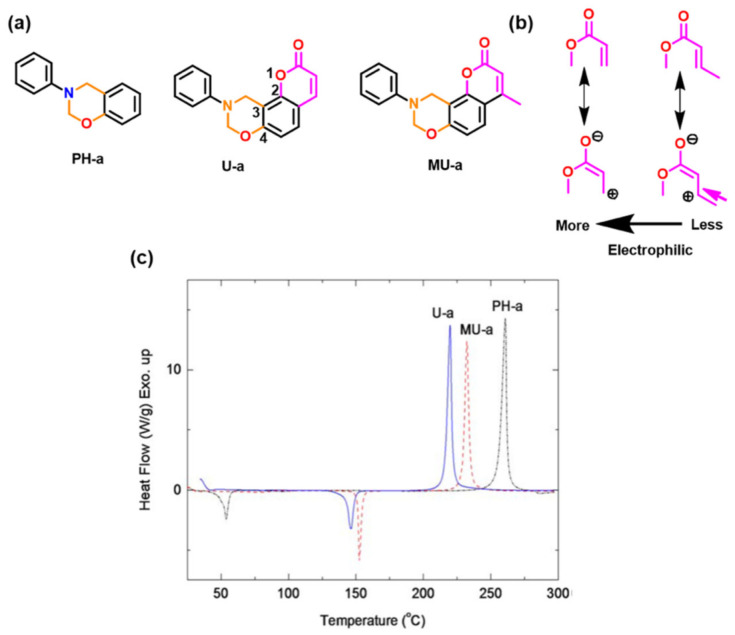
(**a**) Coumarin based monomers, (**b**) schematic depiction showing the resonance of a compound and the electronic implications, evidencing the origin of the activated electrophilic olefins, also referred to as Michael acceptors, and (**c**) DSC thermograms of the benzoxazine monomers [210]. Copyright 2015. Reproduced with permission from 2015 Wiley Periodicals, Inc.

Besides the capability for photo-induced dimerization, the coumarin as compared with non-coumarin containing monomers (Figure 19a) showed a low *T*_p_ for ROP reaction [210]. High stability of the ring opened phenoxide ion via extended resonance occurs in both U-a and MU-a. This resulted in a reduction in *T*_p_ of PH-a from 261 °C to 220 °C (U-a) and 232 °C (MU-a) as illustrated in Figure 19b,c [210]. The methyl group in coumarin in MU-a indirectly affects the reactivity of oxazine by being less electrophilic, i.e., less electron-withdrawing in nature, and thus, it has lower stability and hence accounts for observed higher ROP temperature than U-a. Alagar et al. [214] extended copolymerization of chalcone benzoxazine with bismaleimides to improve thermal stability of polymers.

**Figure 20:**
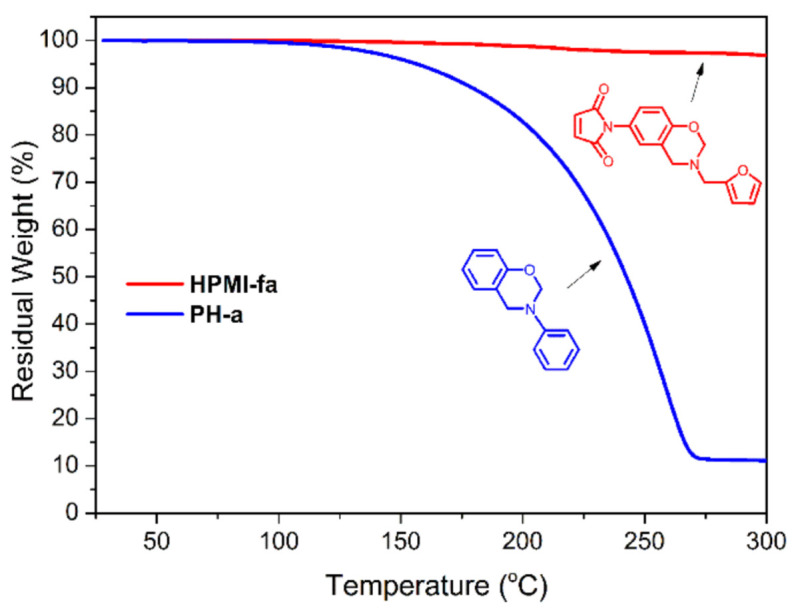
Thermogravimetric analysis of HPMI-fa and PH-a, showing nearly complete prevention of the evaporation of the resin prior to polymerization [215]. Copyright 2019. Reproduced with permission from American Chemical Society.

As discussed previously, mono-oxazine functional monomers suffer from a few problems. They tend to evaporate significantly prior to polymerization and, upon polymerization, form small oligomers with molecular weight of several hundred to few thousands, despite their processing advantage of very low viscosity and ease of purification upon synthesis. Zhang et al. [215] reported the first example of AB-type benzoxazine monomer having maleimide and furan as the terminal functionalities of the monomer. They proposed that the Diels–Alder reaction takes place between the maleimide and furan below the polymerization temperature of oxazine to form small oligomers of several repeat units during melt processing. This approach maintains the advantage of very low viscosity mono-benzoxazine yet prevents evaporation of the monomer, as the increased molecular weight of the oligomer means it will not evaporate at the processing temperature as compared in Figure 20 for mono-oxazine benzoxazine monomers with and without Diels–Alder reacting pair. This further leads to a crosslinked polymer chain structure that exhibits good mechanical and thermal properties by reducing the number of dangling chain ends, a usual issue in mono-benzoxazine polymers. The main-chain type oligomers with several repeat units have been reported to offer good trade-off between processability and high thermal properties as discussed in Section 2.2. They exhibit high char yield, resulting in one of the smallest heat release capacities (HRC), 33.4 J/g.K, of all polymers. Certain monomers possess self-catalyzed cationic ring-opening polymerization for example, deoxybenzoin-based benzoxazine monomer [216]. Lowering the polymerization temperature reduces the chance of monomers to evaporate.

### 2.3. Third Generation Benzoxazines

The third generation consists of reactive PBzs (with both thermoplastic/thermoset characteristics), namely, (i) main-chain type, where oxazine group is part of every chemical repeat unit of the oligomer chain; (ii) side-chain type based on thermoplastic backbone, where oxazine ring is part of the side chain of every chemical repeat unit; and (iii) telechelic, where oligomer of any polymer chain is terminated by reactive oxazine functionalities at each of the chain ends.

In general, mono-oxazine based PBzs containing several dangling-chain ends account for a lower thermal stability [131,217,218,219,220]. This concept of the chain end of PBz being the cause of the lowest degradation temperature of multiple degradation scheme was first presented by Chernykh et al. [221].

**Figure 21:**
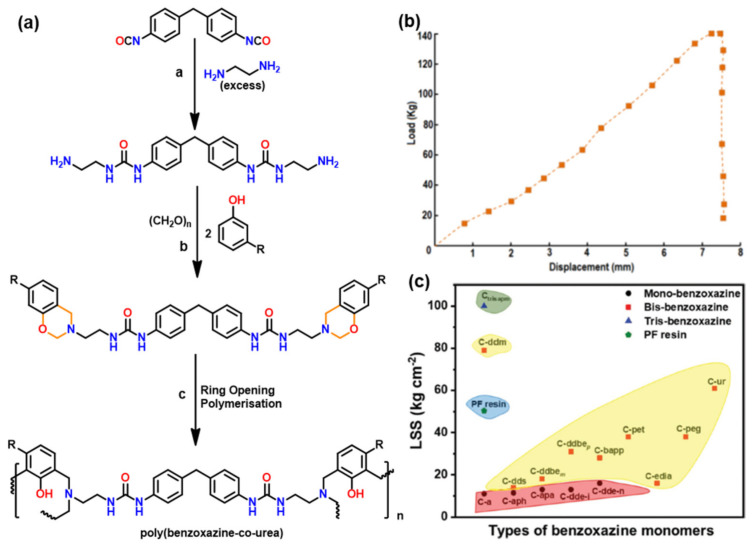
(**a**) Synthesis of main chain-type urea linked polybenzoxazine and (**b**) representative stress-strain curve for a stainless steel coupon held together with poly(benzoxazine-co-urea) as adhesive for LSS measurement [225]. Copyright 2017. Reproduced with permission from John Wiley & Sons, Inc. (**c**) Adhesive strength of polybenzoxazines based on cardanol [90]. Copyright 2018. Reproduced with permission from WILEY-VCH Verlag GmbH & Co. KGaA, Weinheim, Germany.

The incorporation of benzoxazine moieties as repeating units into polymer main chains reduces the number of chain ends in the crosslinked polymers, thus providing an enhanced thermal stability. Therefore, if the mono-amine used as the raw material contains an additional reactivity either by having reactive substituent or added reactivity of the phenyl ring by regioselectivity, the thermal stability of the polymer was found to improve substantially [141]. This main-chain type architecture provides benefits of both thermoplastic and thermoset polymers. Advantages include both solution and melt processability, tunability in *T*_g_ and production of self-supporting films, which further upon crosslinking improve thermal and mechanical strength [222,223,224]. The presence of additional functionalities other than oxazine is beneficial to modify properties, e.g., poly(benzoxazine-*co*-urea) [225], Figure 21a, showed attractive adhesion properties due to existence of polar linkages of both urea and ring-opened benzoxazine and ductile nature of the reactive polymeric chain precursor. The LSS value of poly(benzoxazine-co-urea) determined from plot Figure 21b was found to be 61 kg cm^−2^, which is lower than poly(C-ddm) [87] (79.0 kg cm^−2^). This could be attributed to a higher crosslinking density in the latter and existence of better adhesion properties of ring-opened oxazine structure vs urea linkages in the polymer framework. Cardanol based PBz resins revealed a great potential in many applications [117]. For instance, the adhesive strength of cardanol derived monomers was found to increase with the increase in number of benzoxazine rings in the monomer, as shown in Figure 21c [90]. This enhancement in LSS values is accounted to an interplay of the low viscosity of monomers and flexibility and crosslink density of the polymer.

**Figure 22:**
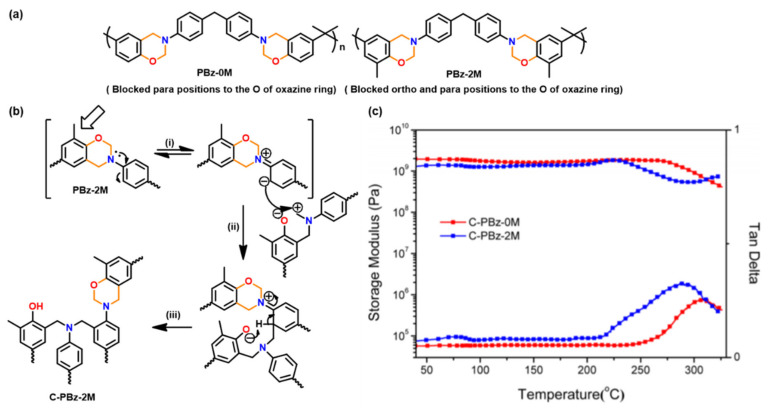
(**a**) Mode of polymerization of main chain type *o*- ad *p*-blocked benzoxazine polymer. (**b**) Proposed ROP Mechanism for PBz-2M [226]. (**c**) DMA thermograms of C-PBz-0M and C-PBz-2M [226]. Copyright 2015. Reproduced with permission from American Chemical Society.

Main-chain type polybenzoxazine with and without free *o*- and *p*-positions to the O of oxazine undergoes polymerization due to the free *o*-position to the N of oxazine are shown in Figure 22. The latter polymer, which is based on the crosslinking from *o*-position to the oxazine N, revealed a lower *T*_o_ and a wider width of the tan δ peak suggesting the ROP occurs through the *o*-position to the O of oxazine led to better thermal properties than that through the *o*-position to the N of oxazine [226].

In summary, main chain type subclass polybenzoxazines provides benefits of an easy synthesis and processability without compromising thermal stability and mechanical property between the polymers obtained from monomeric and high molecular weight oligomeric oxazine precursors. Furthermore, additional reactive functionality other than oxazine ring provides capability to optimize the benefits of both thermoplastic and thermoset polymers. For example, additional functionalities modify the properties of the polymer such as tunability in *T*_g_, solubility in solvents, formation of self-supporting films and may provide opportunities to control over ductility and adhesive properties to name a few.

**Figure 23:**
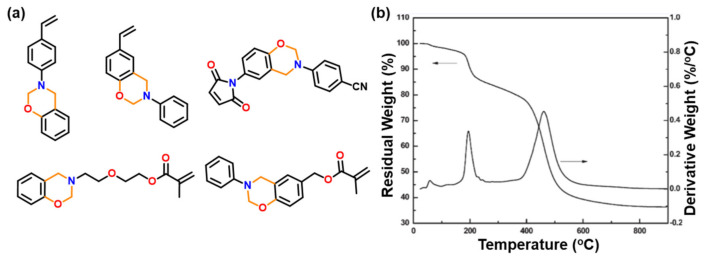
(**a**) Representative photopolymerizable benzoxazine containing monomers [228,229] and (**b**) TGA curve of methacryloyl-benzoxazine monomer [228]. Copyright 2011. Reproduced with permission from American Chemical Society.

Selective introduction of benzoxazine moieties into polymer side chains and terminals [227], and the attachment of radically polymerizable groups such as methacryloyl group to Bz monomers [228,229], led to more variation in functional properties than those discussed above. Few representative structures are shown in Figure 23. Radically polymerizable functionalities in benzoxazine monomers were synthesized to incorporate the benzoxazine units within the side-chain of constituent repeat unit.

Radically polymerizable groups bearing benzoxazine monomers first photopolymerize and are then followed by a thermally activated ROP reaction. However, polymerization of oxazine ring is not realized due to the limit of thermoplastic polymer degradation at a much lower temperature of 200 °C, as can be seen from TGA trace in Figure 23b [228,229]. Copolymerization of poly(PH-*p*va) (va, vinylaniline) with methyl methacrylate (MMA), and *n*-butyl acrylate (BuA) followed by polymerization led to the formation of a tough and flexible film, respectively [230]. The molecular weight of both homopolymer i.e., poly(PH-*p*va) and poly(vPH-a) (vPH, vinylphenol) was found to be relatively low, which is attributed to the steric hindrance of benzoxazine groups and can be affected by copolymerization with *N*-phenylmaleimide [231]. The polymer, poly(PH-*p*va), showed a higher stability (>400 °C), supporting the concept that control at molecular level is the key to guide properties.

**Figure 24:**
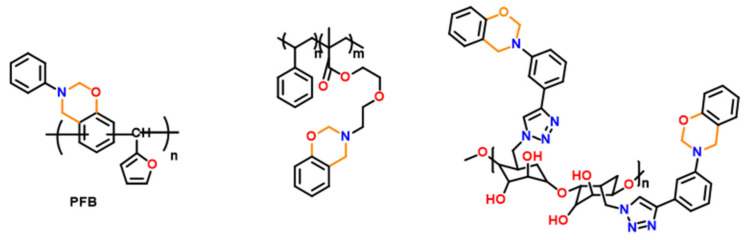
Representative side-chain benzoxazine linked polymers [229,232,235].

Instead of polymerizing monomers containing benzoxazine units, another facile approach involves tethering of pre-synthesized monomers to the polymer backbone directly via potential and affordable chemistries [232] such as click [185,233,234,235], esterification chemistry, and so on [229]. Representative examples are shown in Figure 24.

Interestingly, in the aforementioned side-chain polymers, the benzoxazine units are grafted at a higher concentration. Sparse population of benzoxazine can also be possible by milder photochemical approaches. Temel et al. [236] synthesized a one-component type II photoinitiator based on the BOP-a benzoxazine monomer derived from benzophenone (BoP). This monomer revealed better light absorption characteristics than BoP alone. BoP-a initiated free radical photopolymerization of MMA to form BoP-a tethered PMMA, showing a higher *T*_g_ (135 °C) than neat PMMA (100–105 °C). Bai and Shi [237] reported a BoP based bisbenzoxazine photoinitiator revealed four times higher photopolymerization rate of acrylate monomers than BoP based system.

**Figure 25:**
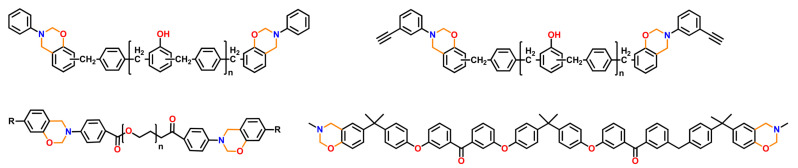
Representative structures of telechelic benzoxazines [46,238,239,240].

Telechelic structures are macromonomers containing non-reactive oligomeric chains in the structure with terminal oxazine groups. The low concentration of oxazine functionality allows for development of lightly crosslinked networks. However, they can be effective for copolymerization with other monomers and polymers. Several reactive thermoplastic telechelic architectures are reported with the variation in linking units from flexible to rigid linkages as shown in Figure 25, to tailor their properties [238]. Unfortunately, due to the dilution effect of the reactive oxazine rings, the *T*_p_ might increase as compared with ordinary monomeric counterparts.

### 2.4. Fourth Generation

#### 2.4.1. Smart Benzoxazines

**Figure 26:**
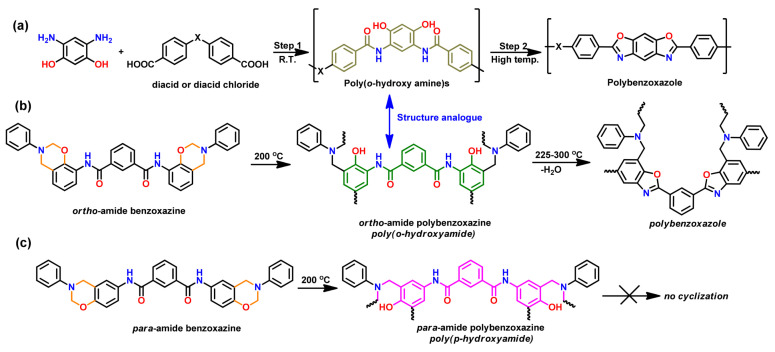
(**a**). Two-step condensation synthesis of polybenzoxazole via intermediate poly(*o*-hydroxyamine); (**b**,**c**) probable synthetic route of polybenzoxazole from *o*-amide benzoxazine monomer, *p*-amide benzoxazine monomer disfavor cyclization reaction [193].

Despite having outstanding performances, polybenzoxazoles (PBO), usage is still limited both by synthetic and fabrication challenges. Additionally, the stringent synthetic requirements, such as expensive and higher structural rigidity of raw materials as shown in Figure 26a, sensitive reaction conditions, and difficulty of completely eliminating the solvent used leads to aging of the properties. This demands exploration of alternative simpler and facile routes. Recently, a new class of benzoxazine monomers and main-chain type polymers that contain *o*-amide, *o*-imide, and *o*-amide-imide groups have been reported [56,195,198,241]. These benzoxazine resins undergo usual ROP to form crosslinked PBzs at temperatures lower than ordinary benzoxazine polymerization. Upon further heating the crosslinked polybenzoxazines, *in situ* structural transformation takes place that converts polybenzoxazine to PBO. Correlating this intermediate structure with the smart designing of the benzoxazine monomer, Agag et al. [193] first reported an easier synthetic route for PBO via polybenzoxazine through the process shown in Figure 26b. This synthetic aspect was further confirmed as only *o*-amide benzoxazine isomer account to PBO formation, but *p*-amide benzoxazine failed to undergo such intramolecular cyclization reaction, as illustrated in Figure 26c.

**Figure 27:**
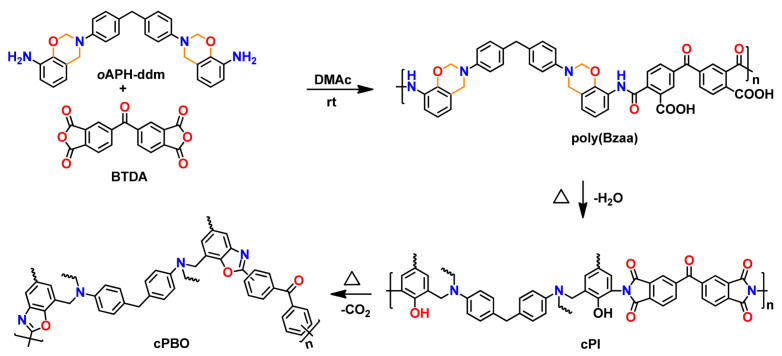
Preparation of cPBO via poly(Bzaa) from benzoxazine monomer [241].

The work was further extended by Zhang et al. [241,242] where they reported formation of crosslinked PBO via poly(benzoxazine amic acid), poly(Bzaa), using *o*-benzoxazine chemistry as shown in Figure 27. Synthesis involved imidization to form crosslinked polyimide, (cPI) which upon further heating undergoes simultaneous decarboxylation and cyclization reaction to give crosslinked polybenzoxazole (cPBO).

The synthetic route was further simplified by the exclusion of amic acid intermediate via small molecule *o*-imide functional benzoxazines as a precursor for structural transformation to PBO. The polymer showed a very high thermal stability with *T*_5%_ of 505 °C and an extremely high char yield of 73% [195]. Interestingly, *o*-amide functional benzoxazine monomers are found to undergo ROP at a much lower temperature than both the *p*-isomer and non-amide-based control monomer. The *o*-isomer revealed a lower polymerization temperature than the *p*-isomer due to the more stable intramolecular 5-membered ring hydrogen bonding interaction that accelerates the ring-opening process [243]. The above polymers revealed an extraordinary low heat release capacity (HRC) further making them strong contenders to replace poly(ether imide) and other high performance polymers.

**Figure 28:**
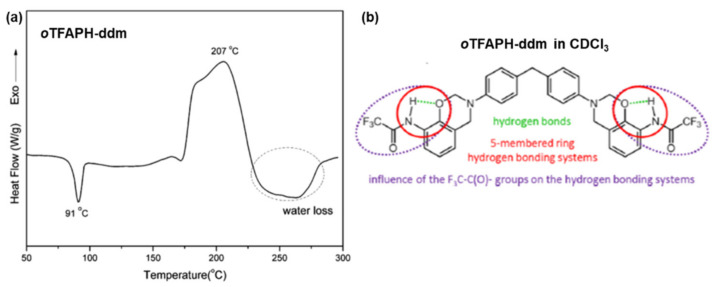
(**a**) DSC thermogram of *o*TFAPH-ddm and (**b**) five membered-ring hydrogen-bonding system at each side of the symmetric resin in chloroform. Copyright 2017. Reproduced with permission from American Chemical Society.

In above-mentioned methodologies, the temperature required for conversion of *o*-amide benzoxazines and *o*-imide benzoxazines into benzoxazole structure was found to be high, ~300–400 °C, which led to difficulties during their fabrication. Zhang et al. overcame this difficulty by developing the endcapping trifluoro group. The incorporation of *o*-trifluoroacetamide in benzoxazine (*o*TFAPH-ddm) exhibits a low polymerization temperature compared to other reported *o*-amide benzoxazines due to higher acidity of amide hydrogen, Figure 28. Furthermore, a remarkably low temperature (260 °C) for interconversion to benzoxazole is observed. Additionally, existence of fluorine in PBO led to an attractive feature of low dielectric constant (2.42–2.19 within the frequency range of 1 Hz–1 MHz) in addition to high thermal stability and *T*_g_ of 354 °C.

**Figure 29:**
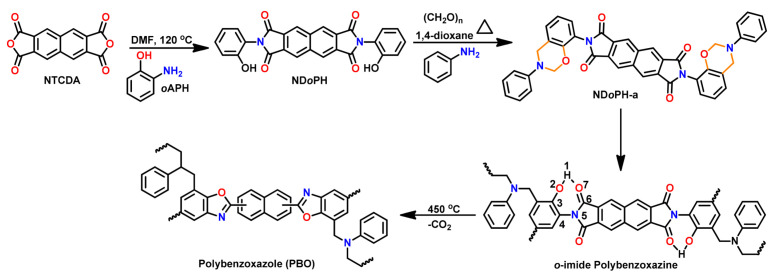
Direct synthesis of o-imide benzoxazine monomer followed by polymerization [244].

Mahdy and Kuo [244] reported a direct synthetic route for the preparation of *o*-imide containing benzoxazine monomer by condensation of anhydride (NTCDA) with *o*-amino phenol (*o*-APH) to produce *o*-imide phenol (ND*o*PH) which was then condensed with paraformaldehyde and aniline to form *o*-imide benzoxazine as shown in Figure 29. The monomer revealed miscibility with poly(4-vinylphenol) due to hydrogen bonding between C=O and OH groups. Upon polymerization of the monomer, the extensive H-bonding between polybenzoxazine led to an improvement in *T*_g_, thermal stability, and char yields of the resultant polymer blends.

**Figure 30:**
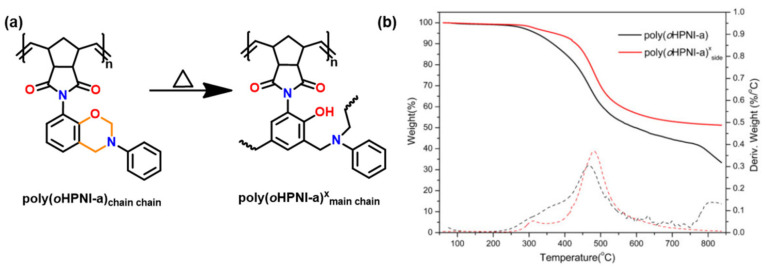
(**a**) Syntheisis of ring-opened main chain polymer; (**b**) thermogravimetric analysis of poly(*o*HPNI-a) and poly(*o*HPNI-a)^x^_main chain_ [245]. Copyright 2019. Reproduced with permission from Sage publications.

Zhang et al. [245] reported the synthesis of side chain containing an *o*-imide functionality to norbornene functional benzoxazine ring as shown in Figure 30. This allowed the monomer to undergo ring-opening metathesis polymerization (ROMP) to form a new polynorbornene functionalized benzoxazine. However, its interconversion to substituted PBO is not reported due to limitations of thermal stability of poly(*o*HPNI-a)^x^_main side_ as determined from the TGA curve in Figure 30b. 

#### 2.4.2. Oxazine Ring Substituted Benzoxazine

**Figure 31:**
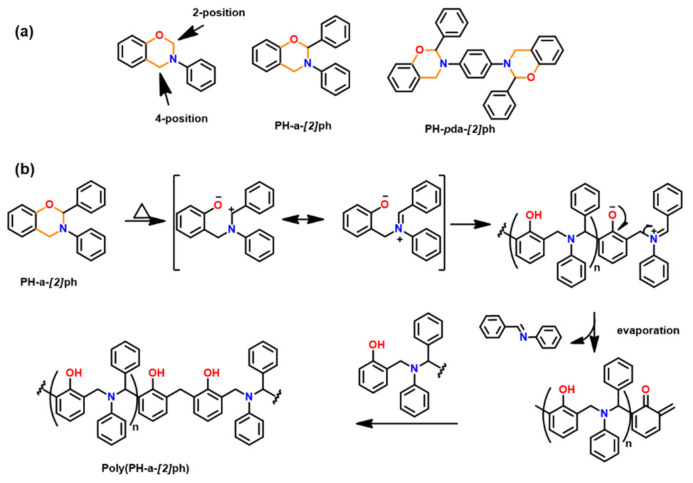
(**a**) Examples of oxazine ring substituted benzoxazine monomers; (**b**) proposed polymerization mechanism [247].

Both the synthesis of 1,3-benzoxazine monomer and polymer and their properties are widely reported in literature. Supplementarily, substitution in oxazine ring at 2- and/or 4-position as shown in Figure 31 presents an attractive structural design, especially in polymer chemistry [246,247]. The nomenclature adopted in such structures is an extension to earlier proposition of benzoxazine structures. For abbreviation of the ring substituted benzoxazine, the following nomenclature is proposed: abbreviated benzoxazine monomer name-[position of substitution in oxazine ring] abbreviated phenyl/alkyl in lower case characters. Thus, for example, PH-a with phenyl ring substitution at 2-position is represented as PH-a-[*2*]ph.

Ohashi et al. [247] first reported polymerization of phenyl substituted 1,3-benzoxazines. The substitution at 2-position is achieved via typical multi-step synthesis from salicylaldehyde involving condensation with benzaldehyde instead of formalin for ring closure reaction. The substitution in oxazine ring allowed a lower polymerization temperature than the general benzoxazines due to the resonance stabilization of iminium ion intermediate as shown in Figure 31b.

However, polymerization of oxazine ring-substituted monomers is currently limited due to evaporation of lower molecular weight *N*-benzylideneaniline molecule as a by-product accounting for a lower thermal stability of polymer. Alternatively, formation of ladder-type bisbenzoxazine PH-*p*pda-[*2*]ph lowered issues of volatilization, due to heavier and bulky nature of imine accounting for its entrapment in polymerization matrix and subsequent involvement in the polymerization reactions. Such losses can also be minimized to some extent by using catalyst mediated ROP at low temperature [248]. More recently, lack of the use of formaldehyde during the synthesis was emphasized as an environmentally friendly method by Travernier et al. [249].

#### 2.4.3. Fused Ring Benzoxazines

**Figure 32:**
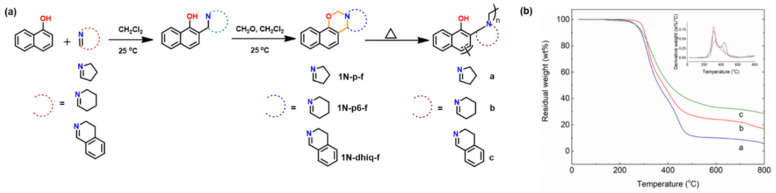
(**a**) Synthesis of fused ring benzoxazine monomer and proposed polymerization, (**b**) TGA thermograms of (**a**) poly(1NP-p6-f), (**b**) poly(1NP-p-f) and (**c**) poly(1NP-dhiq-f). Inset shows the derivative of the weight loss of poly(1NP-p6-f) (green line), poly(1NP-p-f) (blue line) and poly(1NP-dhiq-f) (red line) as a function of the temperature [254]. Copyright 2017. Reproduced with permission from American Chemical Society.

Fused-ring benzoxazine structures contain a sandwiched oxazine ring between an aromatic ring and a cycloaliphatic ring 1-naphthol (1-NP) [250] or 2-naphthol (2-NP) [251,252]. This approach involves a two-step synthesis, firstly of cyclic imines and activated phenols reacted to form aminocycloalkylphenols by the Betti reaction [253]. Secondly, ring-closure reaction of amino derivative with formaldehyde as shown in Figure 32 is carried out [254]. The nomenclature follows numbering of the positions on the oxazine ring as in benzoxazine nomenclature. The aromatic portion contains numbers accompanied by the character “x” as abbreviation for the aromatic ring (b and n for benzene, and naphthalene, respectively) and finally the fused ring using alphabetic letters. For instance, 1-NP and formalin (f) based benzoxazine fused with 3,4-dihydro-2H-pyrrole (p) will be abbreviated as 1NP-p-f. Arza et al. [254] reported the polymerization of fused ring benzoxazine. These monomers show a much lower *T*_p_ as compared to the conventional mono-oxazine monomers. This is due to high basicity of tertiary amines and van der Waals’ strain in sandwiched oxazine ring geometry. Despite interesting structure, low temperature mass losses of polymers as shown in TGA trace Figure 32b demands exploration of alternative synthetic designs.

## 3. Acceleration of the Rate of Polymerization via Intermolecular Interaction

Benzoxazine monomers tend to undergo thermally accelerated ring-opening polymerization in the temperature range 160–220 °C. This temperature range varies with the structure, number of oxazine rings, regio-position, and most importantly, purity of the monomer. In general, mono-oxazine benzoxazines have a *T*_p_ of ca. 250 °C. A low polymerization temperature is desired to improve processibility and compatibility with many other resins and substrates. Alternatively, physical blending of catalysts and initiators with the Bz monomer is one of the most efficient and economically viable approaches for the lowering of polymerization temperature [255,256,257,258,259].

**Figure 33:**
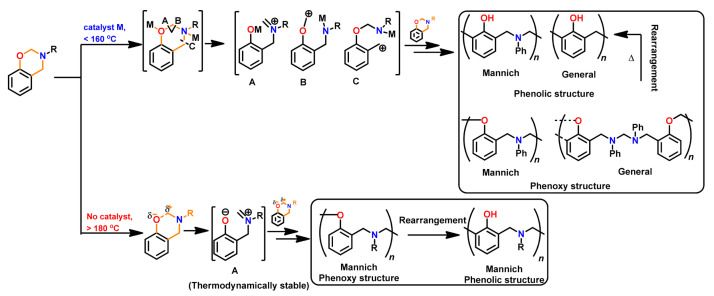
Proposed polymerization mechanism to reflect the effect on structure linkages in PBz with variation in temperature and catalyst.

Burke and co-workers [2,260] first reported the ring-opening reaction of Bz, indicating that aminoalkylation preferred to occur at the free *o*-position rather than at the free *p*-position of the phenol in the reaction of 1,3-dihydrobenzoxazine with a phenol. Riess et al. [261] also observed preference of *o*-substitution during the investigation of the kinetics of mono-functional benzoxazines in the presence of catalytic amounts of 2,4-di-*tert*-butylphenol. McDonagh and Smith [262] suggested existence of ring-chain tautomerism in protonated benzoxazine. Dunkers and Ishida [256] proposed cationic ROP mechanism of Bz monomers. It occurs by protonation of the oxygen/nitrogen atom in oxazine ring in formation of an iminium ion intermediate. This then undergoes several electrophilic aromatic substitution reactions to form the PBz framework. A simplified structure of this phenolic polymer representation is shown in Figure 33. The oxazine ring in the monomer with free *o*-position undergoes a cleavage reaction at O-CH_2_-N bond to form a polymer with a Mannich base phenoxy-type polybenzoxazine structure [255]. This then rearranges to a thermally stable Mannich type phenolic polymer structure upon heating.

The Ishida group worked extensively in determining the thermally accelerated polymerization mechanism and supported ROP proceeds through cationic mechanisms [42,64,263,264,265,266,267]. The plausible mechanism is shown in Figure 33. More recently Liu et al. [268] provided an experimental evidence to support the intrinsic ROP without an added initiator using a high purity monomer, PH-a.

**Figure 34:**
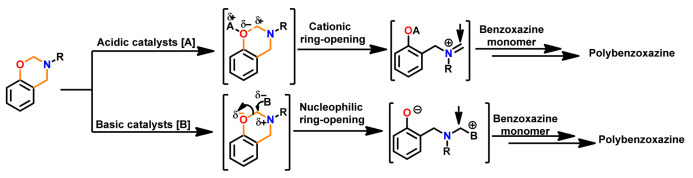
Proposed mechanism of ring-opening reaction in presence of acid and basic catalysts [268].

The ring-opening polymerization is primarily guided by the strong Lewis basicity of both N and O atoms present in the oxazine ring. It was proposed that the acidic catalyst allows polymer network growth via cationic ring-opening reaction while basic catalyst proceeds by nucleophilic ring-opening reaction as illustrated in Figure 34 [268].

**Figure 35:**
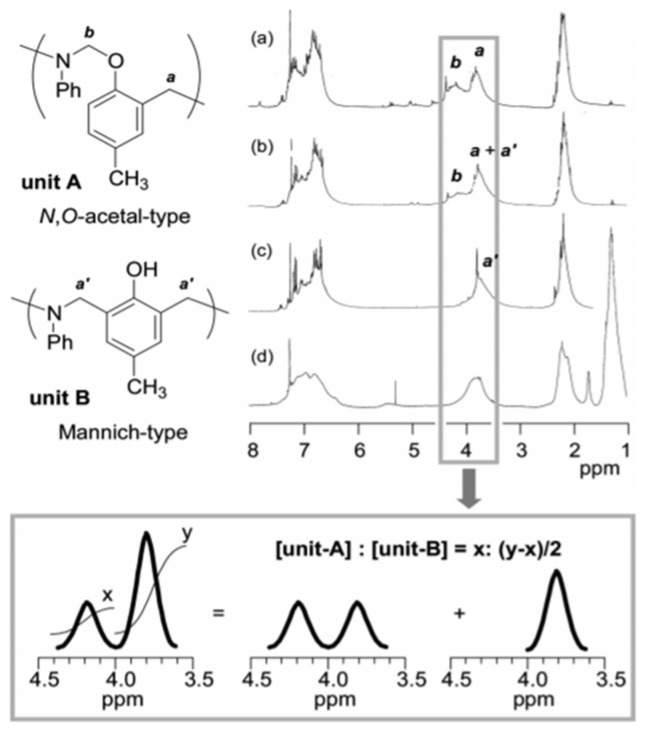
^1^H NMR spectra of the polymer obtained after heating for (**a**) 5 h at 150 °C in the presence of 1 mol% EMI, (**b**) 9 h at 150 °C in the presence of 1 mol% EMI, (**c**) 1 h at 200 °C and (**d**) the polymer obtained by the reaction of the polymer (**c**) and *tert*-butylisocyanate. [269]. Copyright 2008. Reproduced with permission from American Chemical Society.

Wang and Ishida first reported the formation of arylether structure instead of traditional phenolic structure as the repeat unit of polybenzoxazine chains when the monomer was polymerized at low temperatures using catalysts [255]. Sudo et al. [269] then reported that this *N,O*-acetal structure (unit A) formed during polymerization of *p*-substituted monomer at lower temperatures in the presence of a catalyst and can undergo structural transformation to thermally stable phenolic structures (unit B) upon heating above 150 °C. The existence of different types of linkages in polymer structure is guided by temperature, and their extent in polymer architecture is quantified by NMR spectroscopy as shown in Figure 35. Additionally, the main chain rearrangement was prominent in the polymerizations of *N*-aryl- over *N*-alkyl-1,3-benzoxazines, while the latter favors the formation of Mannich-type structure polymers.

An understanding of the polymerization mechanism provides a strategy to utilize or design structures of catalysts/initiators. Done effectively, this provides insight into methods for the acceleration of polymerization. Besides structure of monomers, polymerization behavior is a combination of effects which are primarily guided by the amount, acidity, basicity and structural compatibility of the initiator/catalysts. It must also be noted that purity of monomer is essential as the presence of unconsumed reactants and oligomers initiate polymerization reaction, thereby affecting the rate and temperature required for polymerization. The purity of a benzoxazine monomer has been documented to have a dramatic effect on melting and polymerization behavior [270]. 

### 3.1. Use of Cationic Initiators

#### 3.1.1. Ordinary Acids

**Figure 36:**
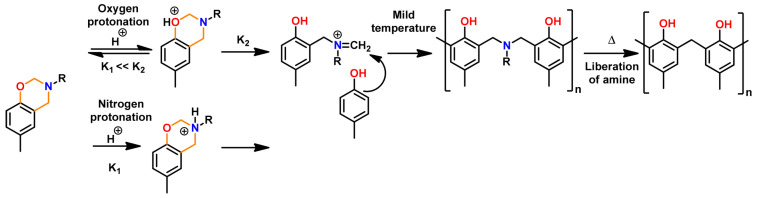
ROP of Bz monomers initiated by proton catalyst [271].

Acidic initiators are usually preferred due to ease of availability and variable acidity to mediate cationic ROP. Acids protonate either the oxygen or the nitrogen atom of oxazine ring and thereby facilitate intermediate iminium ion formation. The former is preferred as the resultant iminium ion intermediate is relatively more stable, as shown in Figure 36 [271]. Repetitive electrophilic reactions involving the generated intermediate and the *O*-attack, *N*-attack, and aryl-attack by the Bz monomer results in the growth of polymer, which may contain phenoxy and phenolic linkages. In structures where *o*-positions are blocked or sterically hindered, polymerization is expected to occur at an available *p*-position to the benzoxazine ring.

**Figure 37:**
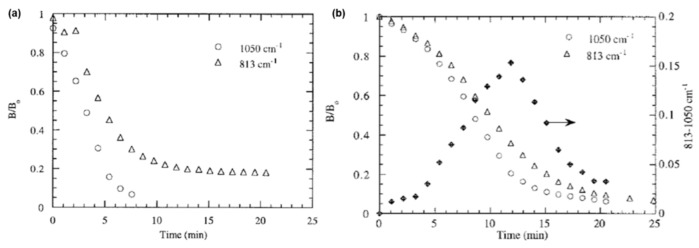
(**a**) Conversion of benzoxazine monomer (*p*C−ma) with (**a**) 10 mol% *p*C and (**b**) 9 mol% sebacic acid at 160 °C [256]. Copyright 1999. Reproduced with permission from John Wiley & Sons, Inc.

Ishida et al. [266] reported the use of phenols with a free *o*-position (BA, poly(*p*-hydroxystyrene), 2,2’-dihydroxybenzophenone and 2,6-di-tert-butyl-*p*-cresol), with mild and strong organic and mineral acids (acetic, adipic, sebacic, benzoic, sulfuric, *p*-toluenesulfonic, phosphoric acids) as catalysts for the ROP of Bz monomer. Adipic acid (6 mol%) was found to be the most effective and showed a 17% decrease in the curing parameters. PBzs obtained using strong carboxylic acids were inferior to those formed with weak carboxylic acids. The pK_a_ of the acid appeared to control the interconversion of the reactive intermediate, aminomethyl ester and the iminium ion affecting crosslinking reactions. Figure 37 shows a faster decrease in the normalized areas of the characteristic oxazine ring vibrations at 1050 and 813 cm^−1^ in presence of *p*-cresol (pK_a_ = 10.2) than sebacic acid (pK_a_ = 4.7, 5.4), supporting that the former mediates faster oxazine ring-opening reaction [256].

The effect of phenol on the polymerization reaction and associated reaction pathways was explicitly understood using the reaction of 2,4-xylenol (2,4-XY) with 3-aryl substituted benzoxazine. The intermediate species formed were several inter- and intra- molecular rearranged products [272]. Bisphenol F acts as a better catalyst than BA which may be due to differential electron donating capability of methylene vs isopropylidene bridge in biphenols [273]. A very high loading (~40 wt%) of phenolic compound, cashew nut shell liquid (CNSL), in BA-a led to a decrease in both *T*_p_ and Δ*H* from 216 °C and 246 J g^−1^ to 197 °C and 194 J g^−1^ [274]. Furthermore, the polymerization reaction proceeds in an autocatalytic manner due to the formation of ring-opened phenolic structures [266], which itself promotes the initiation reaction and induces a catalytic effect. For instance, resorcinol-aniline (R-a) benzoxazine undergoes polymerization (*T*_i_ 146 °C, *T*_p_ 179 °C) at a much lower temperature as compared to PH-a [146]. This is accounted to the formation of two vs one phenolic -OH in ring-opened structure in R-a vs. PH-a.

The low percentage of phenols act as an initiator and affect both rate and the temperature required for ROP reaction. Hamerton et al. [275] reported that TDA (3,3-thiodipropionic acid, pK_a_ = 4.11) is a superior initiator to 3,3-thiodiphenol due to its higher pK_a_ value. TDA has a greater effect on the reduction of *T*_o_ with simultaneous increase in crosslink density as reflected from a high *T*_g_ value in the resultant polymer.

Natural renewable phenolic acids, cinnamic [102], ferulic [101], coumaric [101] and phloretic acids [104] besides their utility as a feedstock for the synthesis of benzoxazine monomer, were also used as catalyst to reduce the polymerization temperature of the polymerization reaction.

#### 3.1.2. Thiols and Elemental Sulfur

**Figure 38:**
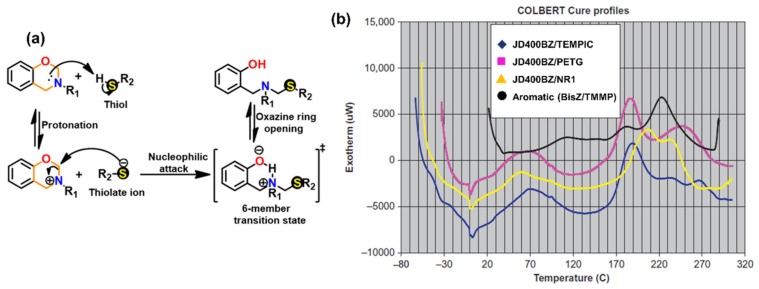
(**a**) Proposed COLBERT reaction mechanism and (**b**) DSC comparisons of aliphatic vs aromatic benzoxazine/thiol polymerization profiles [276]. Copyright 2011. Reproduced with permission from Elsevier B. V.

Unlike phenols, thiophenols react with Bz monomers in a reversible manner at ambient temperature. Additionally, high miscibility, inexpensive cost, and stability towards moisture are encouraging towards their practical use. As compared to phenols, thiol compounds are more efficient at lower weight percent loading due to their active hydrogen donating ability. Gorodisher et al. [276] in 2011, reported addition reaction of thiols with Bz for adhesive application. They proposed a two-step Catalytic Opening of the Lateral Benzoxazine Rings by Thiols (COLBERT) mechanism as shown in Figure 38a. A first step involving protonation of the nitrogen atom of an oxazine ring by thiol was proposed. Subsequently, the thiolate ion attacks >CH_2_ group between heteroatoms to enable oxazine ring-opening reaction. The overall process is similar to acid catalyzed nucleophilic addition and simultaneous ring-opening reaction of benzoxazines. Due to reversibility of the reaction, only small amounts of thiols reduce the polymerization temperature as shown in DSC thermograms in Figure 38b, and due to a continuous regeneration of active species, thiol and iminium ion, which then mediate polymerization reaction.

**Figure 39:**
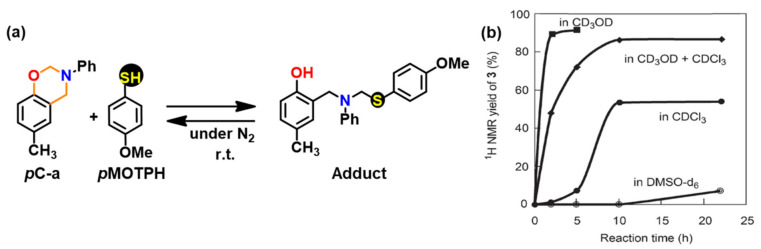
(**a**) Reversible addition-dissociation reaction of *p*C−a with p-methoxythiophenol (MOTPH) at 0.1 M concentration of reagents. (**b**) Time-dependence yield of adduct [280]. Copyright 2014. Reproduced with permission from Wiley Periodicals, Inc.

The protonation of the N or O atom of an oxazine ring by thiol is considered a rate-determining step as supported by catalytic, and inhibitory effect of acids, and bases on ROP of oxazine ring, respectively. It is observed that monomers sourced from aliphatic amines undergo a more rapid reaction with thiols than aromatic amines due to their more basic nature. The rate of reaction is strongly affected by the acidity of thiol and nature of solvent (protic vs aprotic). Kawaguchi et al. [277,278] investigated the reversible nature of polymerization-depolymerization reaction of *p*C-a with *p*-methoxythiophenol (*p*MOTPH) to form a ring-opened adduct as illustrated in Figure 39a. The yield of adduct is higher in polar solvents as compared to polar aprotic solvents, Figure 39b, which is due to stabilization of the polar zwitter ion/ammonium cation intermediate [277,279,280].

**Figure 40:**
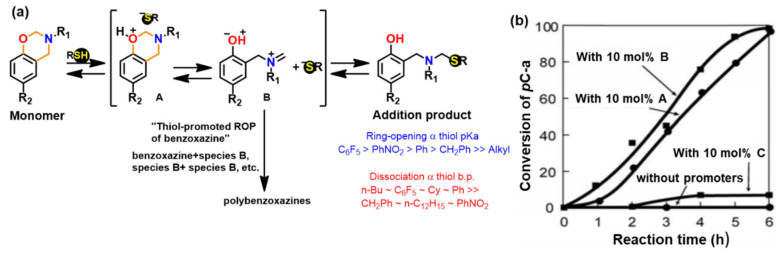
(**a**) Reversible reaction of benzoxazines and thiols [278,281]. (**b**) Time dependence of conversion of *p*C-a in its thermally induced polymerization in the presence of thiophenol (A), *p*-nitrothiophenol (B) and p-cresol (C) at 140 °C [278]. Copyright 2014. Reproduced with permission from Wiley Periodicals, Inc.

Besides monomer, main-chain type PBz was polymerized at room temperature with various thiols, namely, thiophenol, 2-ethanethiol and 1-butanthiol in CH_3_OH/CHCl_3_ for 24 h. Successful incorporation of the thiol compounds to PBz was confirmed by spectral and molecular weight characterizations [279]. Urbaniak et al. [281] proposed that reversible ring-opening of 1,3-benzoxazine with thiols proceeds via an iminium ion intermediate rather than the cyclic six-membered transition state, as shown in Figure 40 [278]. *p-*Nitrothiophenol and thiophenol promotes a substantial increase in % conversion of monomer as can be seen from Figure 40b, which illustrates the strong influence of thiols with low pK_a_ values. Their work emphasizes the relevance of the protonation step in the ring-opening reactions of benzoxazine with thiols in solvent/solvent-free conditions, allowing predominance of acidity over nucleophilicity [277,281].

**Figure 41:**
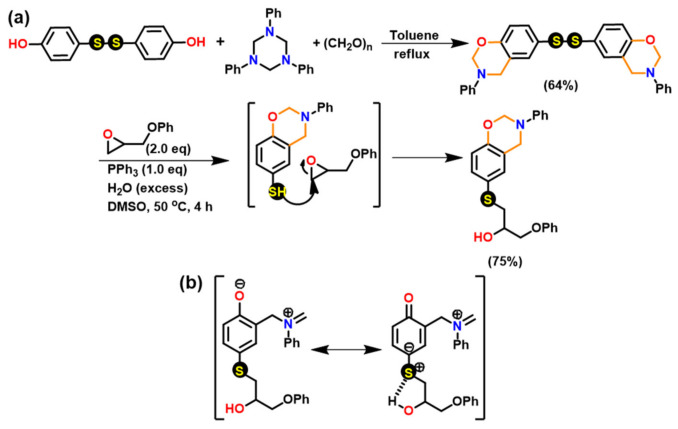
(**a**) Syntheses of sulfide-functionalized benzoxazines by the reaction of the in situ generated thiol Bz with epoxide. (**b**) Plausible acceleration mechanism for polymerization due to stability of the ring-open intermediate [282].

Instead of external thiol addition, in situ generated thiol-functionalized benzoxazine monomer from reductive cleavage of a disulfide linked precursor monomer was trapped with epoxides to form substituted Bz structures as shown in Figure 41a. The newly formed monomer exhibited much higher tendency to polymerize than those without sulfide moiety, due to stabilization of intermediate via dipolar interactions including resonance stabilization, as illustrated in Figure 41b [280,282].

Beyazkilic et al. [283] and Narayanan et al. [284] successfully polymerized Bz monomer by simultaneous photoinduced thiol-ene and COLBERT reactions due to the presence of double bonds and oxazine rings. The oxazine ring-opening and allyl group polymerization in BA-ala vs thiol treated BA-ala were observed at ~230 and ~278 °C vs. 175 and 260 °C, respectively.

**Figure 42:**
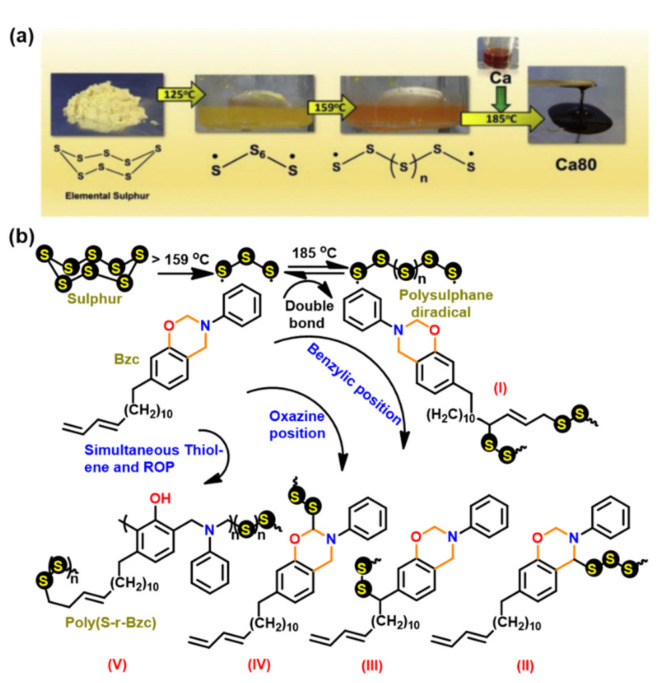
(**a**) Optical images at various stages of copolymerization of elemental sulfur with C-a [21]. Copyright 2016. Reproduced with permission from Elsevier Ltd. (**b**) Probable structure of copolymer formed by reaction of elemental sulfur and C-a of copolymerization [19]. Copyright 2014. Reproduced with permission from WILEY-VCH Verlag GmbH & Co. KGaA, Weinheim.

Besides thiol reagent, elemental sulfur (S) also acts as reactive reagent and was found to reduce the polymerization temperature of benzoxazine monomer [19,21,24,285,286]. Shukla et al. [21] demonstrated lowering of *T*_p_ from 263 °C, in C-a monomer, to the copolymerization (C-a and S_8_) reaction temperature of 185 °C. Figure 42a,b shows optical images of solventless copolymerization reaction at various stages and the possible mode of copolymerization mechanism, respectively. It was proposed that in situ generated polysulfane diradicals react with oxazine ring and double bonds to form poly(C-a-*ran*-S). The copolymers with sulfur demonstrated their utility as cathodic material in Li-S and Na-S battery [19,21,24,287].

**Figure 43:**
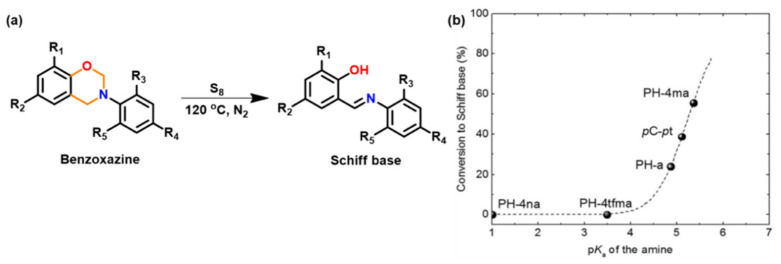
(**a**) Generation of Schiff base from the reaction between benzoxazine resins and S_8_ and (**b**) conversion of Bz monomer to Schiff base as a function of the pK_a_ of the amine in benzoxazine [286]. Copyright 2016. Reproduced with permission from Royal Society of Chemistry.

Arza et al. [286] demonstrated an effect of amine basicity in Bz monomer during co-reaction with S_8_ at 120 °C. They found conversion of monomer to Schiff base in the presence of S_8_ is lower for amines containing electron-withdrawing groups (nitro, trifluoromethyl) than electron-donating groups (methoxy) as shown in Figure 43. Arslan et al. [285] supported existence of radical chemistry and liberation of H_2_S gas during copolymerization of BA-ala with S_8_ in presence of a radical scavenger. 

**Figure 44:**
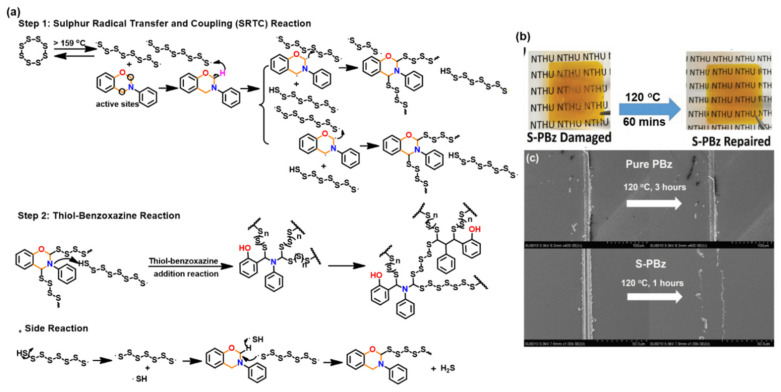
(**a**) Reaction mechanism between S_8_ and benzoxazine through the SRTC reaction and thiol–benzoxazine addition reaction, (**b**) optical images and (**c**) SEM micrographs showing the thermally induced repairing behavior of resins [288]. Copyright 2018. Reproduced with permission from WILEY-VCH Verlag GmbH & Co. KGaA, Weinheim, Germany.

A sulfur radical transfer and coupling (SRTC) reaction was proposed by Lin and Liu [288], as illustrated in Figure 44. PH-a acted as a radical transfer agent for the preparation of copolymers with S_8_. The copolymers revealed superhydrophobicity (with water contact angle ~140°) along with temperature mediated self-healing properties.

#### 3.1.3. Brønsted Acids

**Figure 45:**
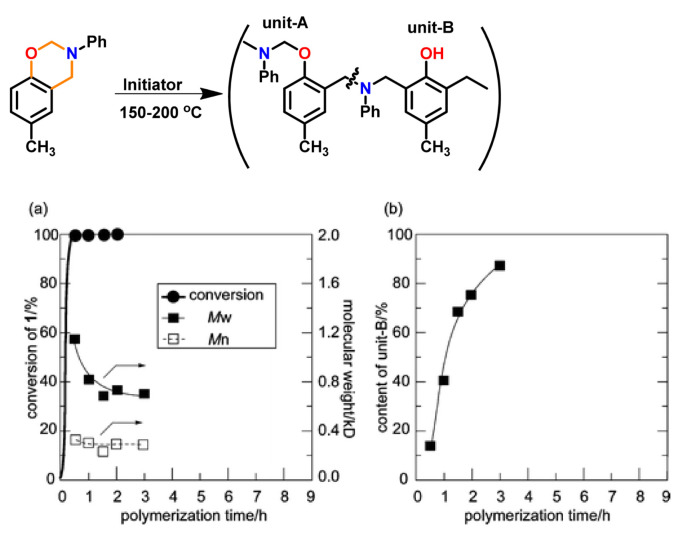
(**a**) Time-dependences of conversion of *p*C-a and molecular weights of the polymer structure and (**b**) content of unit-B in poly(*p*C-a) in the presence of 1 mol% TsOH at 180 °C [269]. Copyright 2008. Reproduced with permission from American Chemical Society.

Brønsted acids, especially *p*-toluenesulfonic acid (TsOH) [255,269,289,290,291] are widely reported to accelerate oxazine ring-opening by the protonation of nitrogen and/or oxygen atoms followed by a transformation into the final reaction products. The percentage of *N,O*-acetal (unit A) and Mannich bridge (unit B) structures in polybenzoxazine are affected by TsOH mediated polymerization of *p*C-a [269]. The former linkages are prominent in the presence of 1 mol% TsOH and low temperature (150 °C). It is evident from Figure 45a,b, at 180 °C, both the monomer consumption and main chain rearrangement were significantly accelerated with the predominance of phenolic Mannich linkages.

**Figure 46:**
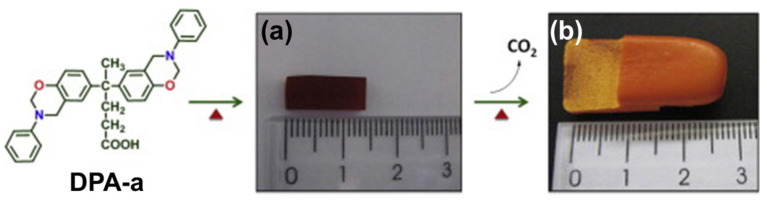
Thermal polymerization of DPA-a monomer. Photographs of (**a**) partially cured polybenzoxazine and (**b**) cross-section of polybenzoxazine foam after heating at 220 °C [111]. Copyright 2012. Reproduced with permission from Elsevier Ltd.

An intramolecular approach involving covalently linked acidic functionalities (Ph-OH, -COOH) [11,256,292,293] within the Bz monomer provides several benefits. They catalyze the polymerization reaction by acting as source of protons and stabilize the ring-opened structures. Substitution of carboxylic group *p*- to O atom of oxazine ring in benzoxazine ring assists stabilization of intermediate ion than when present at the *p*- to N atom of oxazine ring. The carboxylic groups undergo decarboxylation during polymerization and enhance the crosslink density. Furthermore, evolution of CO_2_ led to foaming of the resin, as shown in Figure 46 [11,111].

Hassan et al. [294] developed a low temperature curable reactive blend of BA-a with BA. The incorporation of BA serves a secondary function to increase the reactivity of the blend serving both as an initiator and as co-reactant. With an increase in BA content to 15 wt%, *T*_p_ reduced from 245 to 215 °C, and *T*_g_ from 162 to 147 °C. Beyond 15 wt%, BA incorporation led to a substantial decrease in thermal stability. This is accounted to by the existence of thermally labile BA rich dangling chains in the resultant polymer. Hemvichian and Ishida [295] reported primary decomposition product of PBz are mainly amine, benzene, phenol derivatives. The first mass loss at 150–200 °C is the degradation of BA branches or lightly crosslinked side chains in BA-a/BA polymer [206]. Addition of phenolic nucleophiles (1.5 equivalents), 2-methyl resorcinol (MR), hydroquinone (HQ), pyrogallol (PG) and 2,7-dihydroxynaphthalene (DHNP) to TPHB-a altered *T*_p_ from 238 °C to 133, 118, 115 and 128 °C, respectively. TPHB-a/DHNP copolymer holds industrial importance due to both high thermal stability and a low processing temperature [145]. In addition to the intermolecular catalysis, an intramolecular approach has also been studied. Chen et al. [296] reported heating a mixture of derivative of Meldrum acid and BA-fa monomer assisted ROP. It is proposed that the decomposition of acid produces ketene first, which upon hydrolysis, is converted to carboxylic acid, affecting polymerization.

Sulfonated poly(styrene divinylbenzene) [SP(St-DVB)] microspheres [297] were found to not only accelerate the polymerization of BA-a due to acidic sulfonic groups (decreased *T*_o_ and *T*_p_ values of the BA-a by 39 and 16 °C, respectively) but also increase toughness of the resultant composite. Similarly, incorporation of phenolphthalein polyphosphazene microspheres in BA-a reduced polymerization temperature and led to flame retardance properties due to P and N synergistic effect [298].

#### 3.1.4. Others

Photoinitiated cationic polymerization using diphenyliodonium (Ph_2_I^+^PF_6_^−^) and triphenylsulfonium (Ph_3_S^+^PF_6_^−^) salts led to the light induced generation of protons to form polybenzoxazine (72% conversion). A high monomer concentration (>0.5 mol L^−1^) resulted in oligomer formation, indicating predominance of chain transfer reactions. Besides protons, carbon-centered radicals generated by photolysis of DMPA are oxidized to the corresponding carbocations, which then induce the polymerization of the monomer [299]. Diphenyliodonium salts (ArI^+^ X^−^), reported as moderate initiators, are very effective photoinitiators due to their modest stability and low toxicity for cationic polymerization. In their presence, polymerization profiles of BA-a became broad and started at a much lower temperature (162 °C). An anion dependent effect is observed in the initiation activity of the photoinitiators salt. The polymerization proceeds in two steps: the first step involves generation of H^+^X^−^, and the second step is the initiation of acid-catalyzed ROP of benzoxazine monomer [300].

Filler-mediated catalysis is yet another approach that has attracted researchers in terms of catalysis and additional benefits such as microwave processing [301]. The acidic functionalities on the surface of montmorillonite (MMT) [191,302], polyhedral oligomeric silsesquioxane (POSS) [303], titania [304] and carbon fiber [305] induce a catalytic effect and lower the ROP of monomers.

### 3.2. Use of Catalysts

The lowering of polymerization temperature was mediated by addition of Lewis acid catalysts, [255,269,271,306,307] metal salts [52,259,308,309], metal organic frameworks [310,311], and nanoparticles [13,303,312,313], which catalyze ROP either due to the presence of empty orbitals or metal ions which have interactions with the lone pair of heteroatoms in oxazine ring and mediate ROP.

**Figure 47:**
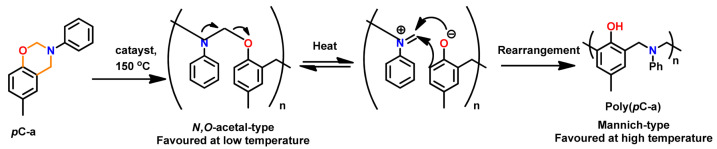
Formation of different linkages in polybenzoxazine framework at different temperatures [269].

**Figure 48:**
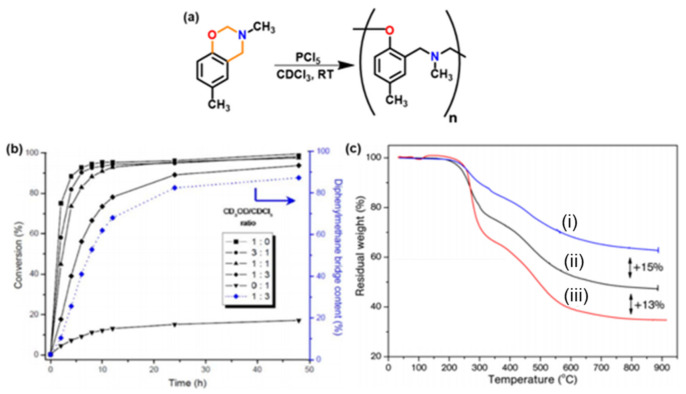
(**a**) Postulated structure of PCl_5_-initiated formation of poly(*p*C-ma) [255]. (**b**) Monomer conversion vs time for the BF_3_.OEt_2_ initiated polymerization of PH-a in CD_3_OD/CDCl_3_ mixtures. Percentage of diphenylmethane bridges vs time in a CD_3_OD/CDCl_3_ (1/3) mixture is also represented. Polymerization conditions: PH-a = 95 mg, solvent = 0.6 mL, [PH-a]_0_ = 0.75 mol/L, [PH-a]_0_/[initiator]_0_ = 40/1, and temperature = 60 °C [314]. Copyright 2013. Reproduced with permission from John Wiley & Sons, Inc. (**c**) TGA traces of poly(PH-ba): (i) neat, (ii) in presence of B(C_6_F_5_)_3_ (ii) 3% and (iii) 5% [315]. Copyright 2018. Reproduced with permission from MDPI.

The nature of phenolic vs *N,O*-acetal polymer network structure is dependent on the nature of initiator. A labile proton initiator, such as phenol, results in phenolic structure while a non-labile proton initiator, such as Lewis acid, leads to *N,O*-acetal structure if polymerization proceeds at a modest temperature. The catalysts affect the polymerization reaction, which proceeds at a lower temperature than usually required, to form phenoxy structures with *N,O*-acetal linkages, which then undergoes thermal cleavage to form the corresponding phenoxide and iminium ion polymeric intermediates. Further electrophilic attack by a benzene ring on iminium ion species form phenolic Mannich- type rearranged structures, which are apparently more thermally stable, as illustrated in Figure 47. Occurrence of these linkages was first proposed by Wang and Ishida as discussed in next Section 3.2.1. and shown in Figure 48a [255].

#### 3.2.1. Lewis Acids

Wang and Ishida [255] first reported Lewis acids such as PCl_5_, PCl_3_, POCl_3_, TiCl_4_, AlCl_3_ and MeOTf mediated cationic ROP of benzoxazines to form *N,O*-acetyl linked polymer structure at room temperature, as shown in Figure 48a. The polymer yield obtained was highest in case of PCl_5_. With PCl_5_ as an initiator, two different polymerization mechanisms competed during the polymer growth, depending on different methyl substitution positions in phenolic ring of the monomers. These mechanisms gave rise to both Mannich base phenoxy-type and phenolic-type polymers [267]. Later Sudo et al. [269] reported the low temperature favored arylether, *N,O*-acetal structure, a structure that is thermally unstable and can undergo structural transformation to the phenolic-type at an elevated temperature (>160 °C).

Even Lewis acid complexes such as BF_3_/H_2_O, PCl_5_, AlCl_3_, BF_3_·OEt_2_, etc. [255,257,314] affect benzoxazine polymerization as they increase the basicity of water and indirectly promote the generation of free protons. This then protonates the oxazine ring, which will lead to ring-opening reaction to generate a carbocation. The resultant carbocation reacts by chain transfer reaction to the benzene ring of another benzoxazine molecule, leading to the formation of dimer and other higher oligomers. BF_3_·OEt_2_ was found to be effective in alcoholic solution under mild conditions, and polymerization proceeds through an intermediate hemiaminal ether, which led mainly to the formation of diphenylmethane bridges, Figure 48b, along with the classical Mannich-type in PBz [314]. Thermal behavior of the PBzs formed using Lewis acids complexes exhibits high *T*_g_ and char yield due to the controlled polymerization conditions. The resultant polymer structure is more ordered as compared to the one obtained during thermal polymerization.

Metal centered compounds accelerate benzoxazine polymerization through the coordination of electron deficient site with the heteroatoms in the oxazine ring. The advantage of utilization of metal complex is due to the ease of tuning of their activity by combination of various metal with appropriate ligands or counter ions. Incorporation of metal simultaneously improves the properties of the resultant composite. Inopportunely, certain metal complexes are relatively intolerant towards moisture, which requires attention during their blending with the monomer, and sometimes the mechanical performance of the material may deteriorate with time. Low and Ishida [52] investigated an improvement in thermal stability of resin on addition of metal chlorides. Transition metal chlorides (~2 mol%) catalyzed polymerization while simultaneously improving the thermal stability and flame retardance characteristics. It appears metal complexes co-ordinate with the lower thermally stable defect structures generated during polymerization such as Schiff base and amide functional groups, thus forming more stable structures before char formation occurs.

Besides lowering of ROP temperature, Sudo et al. [259] studied 4th period transition metals (manganese, iron, cobalt and zinc) acetylacetonato (acac) complexes which allowed ease of processing too. Replacement of ligand from acac with a more electron deficient hexafluoro acac (F_6_-acac) ligand endowed increased Lewis acidity to the complex, and this resulted in an enhanced catalytic activity. Additionally, moisture-tolerance and high activity of F_6_-acac complexes, without their detrimental effect on thermal stability of the polymer, further favor its utility. For instance, acetylene functional benzoxazines undergo thermally activated polymerization where acetylene groups and oxazine rings polymerize simultaneously, releasing large quantities of heat with a sudden increase in viscosity in an extremely short time, affecting processability of pristine monomer. Nickel acetylacetonate hydrate/PPh_3_ allowed controllable and mild polymerization conditions as they prepolymerize acetylene groups to polyacetylene Bz structures. Both *T*_p_ and ∆*H* reduced from 247 °C and 879 J g^−1^ to 152 °C and 555 J g^−1^, respectively, with enhanced thermal stability [316]. Addition of Fe(acac)_3_ (~3.5 mol%) assisted completion of both ring-opening polymerization of oxazine and cyclotrimerization of the cyano group to triazine ring at 350 °C in CN-functionalized Bz [317].

ZnCl_2_ is a more effective catalyst than AlCl_3_ and PCl_5_ for the polymerization, as the resultant polybenzoxazine obtained has a much higher char yield (by 19%) than typical phenolic Mannich type polybenzoxazine with similar *T*_g_ value [306]. Besides the nature of metal centers in catalysts, ease of dispersion of catalysts, such as cerium nitrate, in the monomer is another key factor which govern their catalytic efficiency [309]. Zinc stearate (10 mol%) was found to be the most effective catalyst to lower the *T*_o_ of ROP of bio-based C-a monomer from 242 °C to 169 °C [311]. Like zinc salts, zinc-based metal organic frameworks (MOF) led to lowering of *E_a_* of polymerization from 98 kJ/mol (neat resin) to 58 kJ/mol due to Lewis acidic nature of Zn_4_O nodes. It was suspected the voids between the MOF allowed swift seepage of monomer to access intrinsic Lewis Zn_4_O nodes catalytic centers. The polymer exhibited a relatively high thermal stability (*T*_5%_ = 416 °C) as compared to the pristine polymer (*T*_5%_ = 345 °C) [310].

The presence of empty d-orbitals or electron deficient centers in inorganic materials is prudential for ROP. Silica- and boron-modified polybenzoxazine hybrids demonstrated an enhanced thermal stability along with strong catalytic properties due to the Lewis acidic characteristics of Si and B atoms. Use of trisilanolphenyl POSS [318], phenylboronic acid (PBA) [319], 2-phenyl-1,3,2-benzenediolborane [320], poly(resorcinol phenylboronate) (PRB) [321], tris(pentafluorophenyl)borane [315] is also reported. Especially, with only 3 mol% addition of boron-based Lewis acid B(C_6_F_5_)_3_, a significant increase in char yields (13%) over the pristine polymer is observed, Figure 48c. This is attributed to the high degree of crosslinking induced by the catalyst, coordination between boron (catalyst) and nitrogen atoms (polybenzoxazine) that may delay the amine degradation in polybenzoxazine, and the usual mode contribution of boron and fluorine atoms induced flame retardation effect [315].

#### 3.2.2. Amines

**Figure 49:**
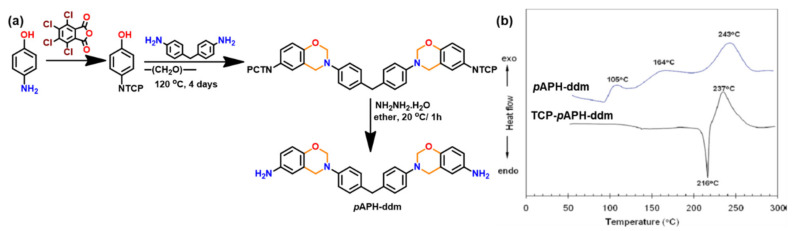
(**a**) Preparation of amino-functional benzoxazine monomers using TCP-protected *p*APH, (**b**) DSC thermograms of TCP-protected and unprotected Bz monomers [322]. Copyright 2010. Reproduced with permission from American Chemical Society.

Agag et al. [322] reported involvement of amine in ROP reaction of benzoxazines. Figure 49a shows the synthetic scheme of monomer, and Figure 49b shows the DSC profile. The *p*APH-ddm revealed multiple exothermic peaks both at a low temperature and at the similar temperature as amine protected *p*APH-ddm monomer. This supports multistage consumption of the amine as co-reactant. The presence of amide linkage in NTCP functional *p*APH-ddm has an effect in the reduction of polymerization temperature.

**Figure 50:**
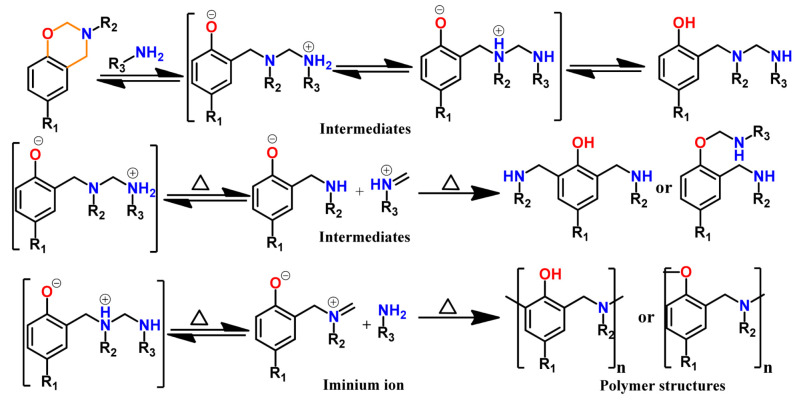
Possible reaction mechanisms between amine and benzoxazine [323].

Recently, Sun and coworkers [323] proposed a reaction mechanism of Bz with amines as shown in Figure 50. The ring-opening addition reaction of amine to benzoxazine and the subsequent progression of the reaction led to polymerization via cationic mechanism. The polymerization reaction mechanism involves a reversible reaction of the amine with benzoxazine to form the zwitter ionic intermediates with both phenolate and aminomethanaminium structures. Upon heating at elevated temperature, the aminomethanaminium moiety decomposes to an iminium ion and proceeds towards an electrophilic substitution reaction with the aromatic ring to form a stable aminomethylphenol structure. Amines act as the nucleophile and allow polymerization to proceed at a much milder temperatures (120–150 °C) with faster rates. The *E*_a_ for polymerization reactions follows the basicity order of amine, arylamine > alicyclic amine > alkylamine.

Primary, secondary and tertiary amines successfully assist ring opening reaction of mono-oxazine in polar solvents, and crosslinking reactions was realized in case of reaction of diamine with bis-benzoxazine at room temperature [324]. Addition of polypyrrole (PPy, 5 wt%) also mediates the ROP reaction, and *T*_p_ value was significantly reduced for C-fa and BF-A, from 245 to 185 °C and 226 to 165 °C, respectively [325]. Imidazoles with and without the labile hydrogen are also reported as good catalysts [326,327,328,329,330].

#### 3.2.3. Latent Catalysts

Mixing active initiators/catalysts with benzoxazines often initiates ROP at low temperature and increases the viscosity significantly during the storage, thus, reducing the shelf life for practical use. Latent catalysts are usually dormant and have minimum activity under normal conditions thereby eliminating storage issues [290,331]. The latent curing systems are usually pre-mixed formulations containing both monomer and hardening reagent, to allow ease of usage with controllable processing characteristics [332]. Generally, they are in form of salt and liberate the active components by an external trigger, which is usually heat [331,333]. Both the anionic (acid) and cationic (basic) species may be involved in the overall polymerization process. They can be organic, inorganic or both. Besides decreasing the polymerization temperature, they also affect the nature of linkages in resultant polymer. This results in variation of both thermal and mechanical properties from those of the pristine polymers [17,334]. Latent catalyst works based on the combined mechanisms. Typically, when the system temperature is raised to the reaction temperature, a heterogeneous latent catalyst undergoes a transition, such as melting, to become an active catalyst before the temperature reaches the polymerization temperature. Some homogeneous latent catalyst achieves time delay based on the non-linear kinetic activation.

##### Heterogeneous Latent Catalysts

These are solid catalysts and are nearly insoluble in monomer at room temperature. Their existence in different phase is mainly responsible for the suppression of their catalytic activity. However, they induce adverse effects on both fluidity and viscosity of the polymerizing reaction mixture. In this class, the catalysts reported are alkyltosylates (ROTs) [258,290,334], TsOH/amine (isopropanolamine or methylamine) [335], ammonium chloride [336], lithium iodide [307], etc.

**Figure 51:**
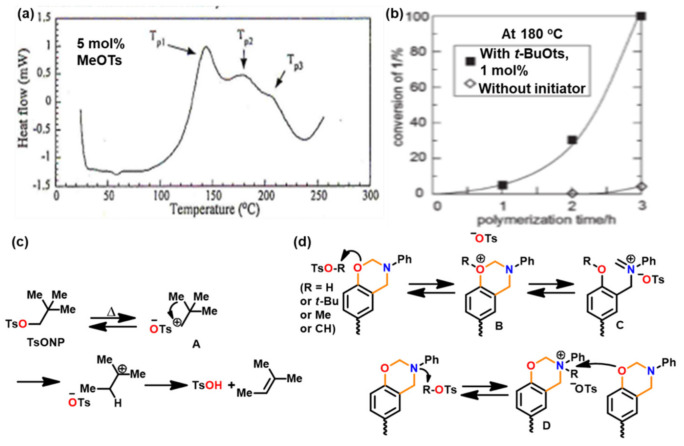
(**a**) Dynamic DSC thermogram of BA-a/5 wt% MeOTs [258]. Copyright 1999. Reproduced with permission from American Chemical Society. (**b**) Time conversion relationship for the tosylated initiated polymerization [290]. Copyright 2011. Reproduced with permission from Wiley Periodicals, Inc. (**c**) Mechanism for the formation of TsOH by thermal dissociation of CH-OTs (CH cyclohexyl) and *tert*-BuOTs and (**d**) possible pathways involved in the initiation by alkyl tosylates [290].

Wang et al. [258] reported addition of methyl *p*-toluenesulfonate (MeOTs, 5 wt%) in BA-a resulted in a complex and broad DSC polymerization profile with multiple exothermic peaks at 144, 179 and 200 °C as compared to neat BA-a (*T*_p_ 231 °C), shown in Figure 51a. These low temperature peaks are assigned to the polymerization reaction of BA-a by cationic initiation by MeOTs. Replacement of the counter ion from tosylate to triflate in methyl substituted initiator led to a significant increase in *T*_g_ from 142 to 193 °C of the resultant polybenzoxazine inferring variation in the polymerization structure [255].

It is apparent from Figure 51b that the percentage conversion of monomer is nearly 100% in 3 h at 180 °C in presence of *tert*-BuOTs than the pristine monomer. The activity of TsOH is higher than ROTs, and the activity of ROTs varies with the nature of alkyl group. The alkyl *p*-toluenesulfonates acts as “thermally latent catalysts”, and their susceptibility to dissociate at the elevated temperatures is highly dependent on its structure. The generated corresponding alkyl cations, TsOH and/or ROTs initiate polymerization reactions as illustrated in Figure 51c,d [290]. A dual system composed of TsOH and EMI (imidazole) effectively promotes the main chain rearrangement reaction from the initially formed *N,O*-acetal-type to Mannich-type linkage in the solid state [269].

Latent curing salt TsOH with amine catalyzes both polymerization and copolymerization reactions [335,337,338]. The bivalent amine (isopropanolamine, methylamine) was found to be more effective than the univalent amine (diethanolamine or diethylethanolamine). Addition of 10 mol% of latent catalyst to BA-a with bisphenol-A-based epoxy resin led to improvement in *T*_g_ and flame resistance characteristics of the resultant copolymer. This is accounted by the enhanced crosslink density as both the released amine and acid induce both homo- and co-polymerization reactions [337]. It must be noted that the latent catalyst polymerizes epoxy resin and the resultant polymerization products further catalyzes Bz ring-opening reaction. Thus, it belongs to a secondary latent catalyst for Bz.

**Figure 52:**
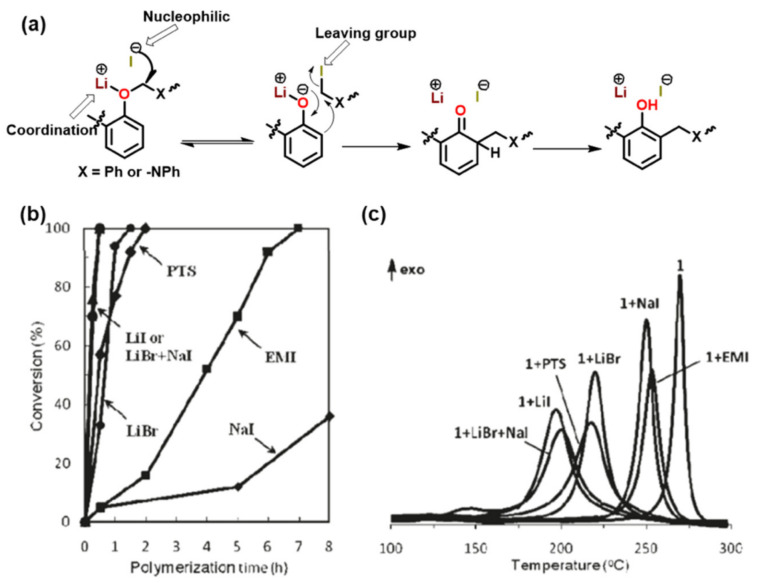
(**a**) Proposed LiI promoted rearrangement reaction, (**b**) time dependences of conversion of pC-a for the polymerization with 1 mol% various catalysts at 150 °C, (**c**) DSC plots of pC-a and its mixtures with 1 mol% of various catalysts [307]. Copyright 2011. Reproduced with permission from American Chemical Society.

Similarly, in polymerization assisted by lithium iodide, lithium cation acts analogous to TsOH due to its very high affinity toward oxazine oxygen atom. Iodide ion serves a dual role, due to its good nucleophilic and leaving group properties [307]. A probable mechanism of LiI catalyzed polymerization is shown in Figure 52a. The obtained polymer mainly furnished phenolic CH_2_ units with a high percentage of true phenolic CH_2_ units. As a comparison, under similar conditions TsOH provided phenolic CH_2_ units with more amounts of general phenolic CH_2_ units, and EMI mainly afforded phenoxy CH_2_ units, supporting the concept that LiI is a better catalyst to favor true phenolic linkages. Figure 52b,c represents time dependence percentage conversion and DSC polymerization profiles of monomers following the order NaI < EMI < TsOH < LiBr < LiBr/NaI ≈ LiI, confirming LiI is more effective and has nearly the same catalytic effect as LiBr + NaI.

Amine HX salts reduce the ROP temperature, and reduction is found to be both cation and counter-ion dependence and follows the order of I^−^ > Br^−^ > Cl^−^ [331]. Recently, cyanuric chloride (2,4,6-trichloro-1,3,5-triazine, TCT) was used as a miscible latent catalyst; it remained dormant at RT and was activated due to moisture or nucleophile to form HCl and cyanuric acid, which then catalyzes the ROP reaction [339].

##### Homogeneous Latent Catalysts

**Figure 53:**
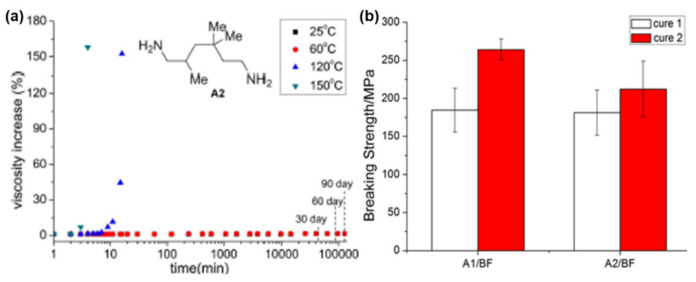
(**a**) Time dependence of viscosity of reactive mixture A2/BF stored at 25, 60, 120, and 150 °C, (**b**) material properties of cured resins: breaking tensile strength [340]. Copyright 2016. Reproduced with permission from Springer Nature.

A homogeneous liquid of benzoxazines with amines forms intermolecular latent curing system based on reaction equilibrium. This strategy differs fundamentally from the blocked hardeners proceeding to release active groups via thermal deprotection. The reversible reaction of Bz and amine acts as a latent curing system, forming a reaction equilibrium with the intermediate structures of low viscosity at room temperature for a long time as discussed in Section 3.2.2. An imbalance in reaction equilibrium is achieved by heating, leading to rapid increase in viscosity due to polymerization as shown in Figure 53a. The pristine polymer, poly (BF-a), showed a lower tensile strength of 44 MPa as compared to PBz (180 MPa) formed by co-reacting with amine, Figure 53b [340]. 

In addition to intermolecularly associated compounds such as a salt or complex, intramolecularly associated compounds such as intra-molecular H-bonded material or zwitterions or intramolecular electronic effects also affect the ROP temperature. The stable intramolecular H-bonding interactions at low temperature between the phenolic -OH group with oxazine ring N-atom [333], or N-H of amide group with oxazine ring O atom [341], showed an enhanced shelf life due to inactive nature/unavailability of such acidic hydrogens. However, such hydrogen-bonded interactions are weakened or disrupted upon increasing the temperature leading to the formation of the free phenolic -OH groups inducing a latent catalytic effect on the polymerization reaction. Phenolic hydroxyl bearing naphthoxazines was found to be a latent catalyst for the ROP of simple 1,3-benzoxazines [342]. The presence of phenolic-OH sandwiched between the two oxazine rings in PG-fa monomer [343] and amide derivative of gallic acid-based main-chain benzoxazines revealed a latent effect [344]. Similarly, hydrogen-bonding between phenolic-OH and carbonyl motif in naturally occurring phenol, naringenin-based benzoxazine monomer, NA-fa, is accounted to have a latent effect and leads to a lower polymerization temperature, 166°C [109].

#### 3.2.4. Nanomaterials

**Figure 54:**
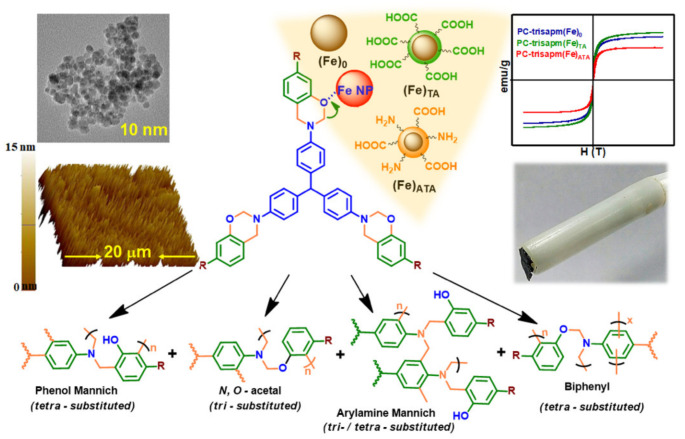
Probable linkages in the PBz network due to iron-oxide NPs mediated polymerization; AFM 3D images indicating surface topography of NPs in PBz; hysteresis curve and digital photos of composite showing magnetic property [90]. Copyright 2018. Reproduced with permission from WILEY-VCH Verlag GmbH & Co. KGaA, Weinheim, Germany.

Organic–inorganic nanocomposites could exhibit unexpected hybrid properties synergistically derived from two components that are dramatically different from their bulk counterparts. Further, due to their nano-size, nanocomposites feature an extensive array of interfacial interactions that can result in salient changes relative to their components properties. The surface of nanomaterials is usually modified to allow their ease of dispersion, reducing aggregation issues, and improve adhesion with the polybenzoxazine matrix. The surface carboxylic and hydroxyl groups on carbon nanotubes [312], core shell rubber (CSR) [345], and graphene oxide [346,347,348], graphene nanoplatelets [349] and metal-oxide nanoparticles (NPs) [13,313] reduce polymerization temperature of benzoxazine monomers with a reduction in *E_a_*. A higher *T*_g_ and char yield is indicative of improvement in intermolecular interactions in the polymerization reaction. Improvement in adhesive strength of steel plates and initial low viscosity of C-a monomer is credited to the higher surface area and acceleration in ROP due to exposition of aluminum atoms on the surface of NPs [13]. Such dual nature of interaction among polybenzoxazine has been studied using inverse chromatography by Xu et al. [350]. A 5 wt% addition of capped iron oxide nanoparticles in benzoxazine monomer endows the benefits of substantial lowering of the polymerization temperature (from 207 °C to 143 °C) and enhancing maximum thermal stability (increase of 34 °C) which is attributed to the iron ions and capping agent. Surprisingly, in addition to the usual chemical linkages in the polybenzoxazine network, Monisha et al. [313] reported the existence of biphenyl linkages due to oxidative polymerization, as shown in Figure 54. Polymer nanocomposites revealed uniform dispersion of iron oxide nanoparticles with good magnetic saturation and superparamagnetic behavior.

#### 3.2.5. Others

Yue et al. [351] investigated the catalytic potential of α-zirconium phosphate (α-ZrP, 3 wt%) and the resulting decrease in *T*_p_ values of BA-a by 18°C with concomitant increase in Δ*H* observed. The poly(BA-a)/α-ZrP composite showed improved thermal properties as exfoliated α-ZrP provided a physical barrier for the spread of flame by retarding the diffusion of both heat and degraded gaseous products.

**Figure 55:**
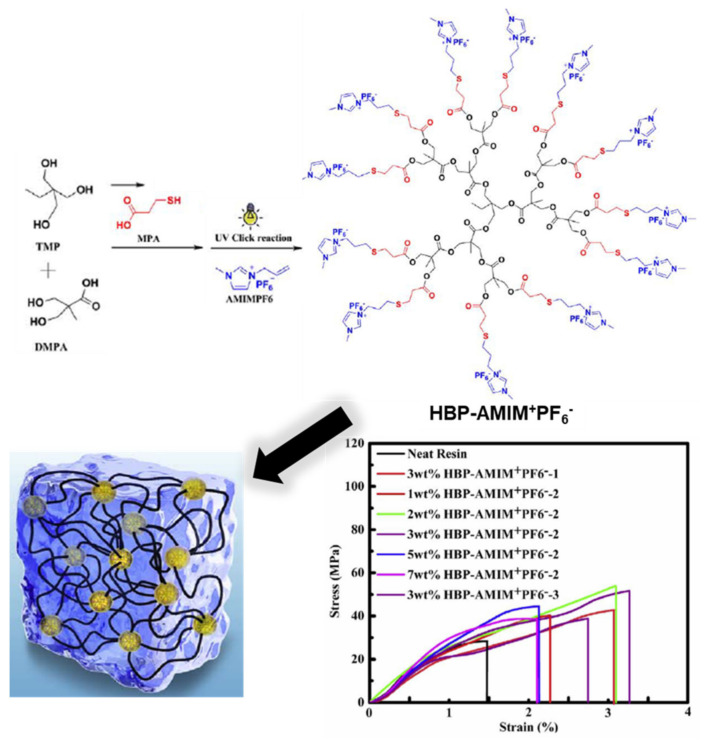
(**a**) Synthesis of hyperbranched polyester ionic liquids. (**b**) Representative stress−strain curves [352]. Copyright 2017. Reproduced with permission from Elsevier B. V.

Chen et al. [352] reported on the addition of a hyperbranched polymeric ionic liquid obtained via a thiol-ene click reaction. Thiol-ended hyperbranched polyesters and 1-allyl-3-imethylimidazolium hexafluorophosphate to BA-a and epoxy blend lowers both gelation time and polymerization temperature with an improvement in mechanical strength of benzoxazine/epoxy thermosets, Figure 55 and Table 4. The toughening mechanism was attributed to the in situ reinforcing and toughening mechanisms [352].

### 3.3. Others

#### 3.3.1. Intermolecular Influence on Oxazine Ring-Opening Equilibrium (OH Groups)

**Figure 56:**
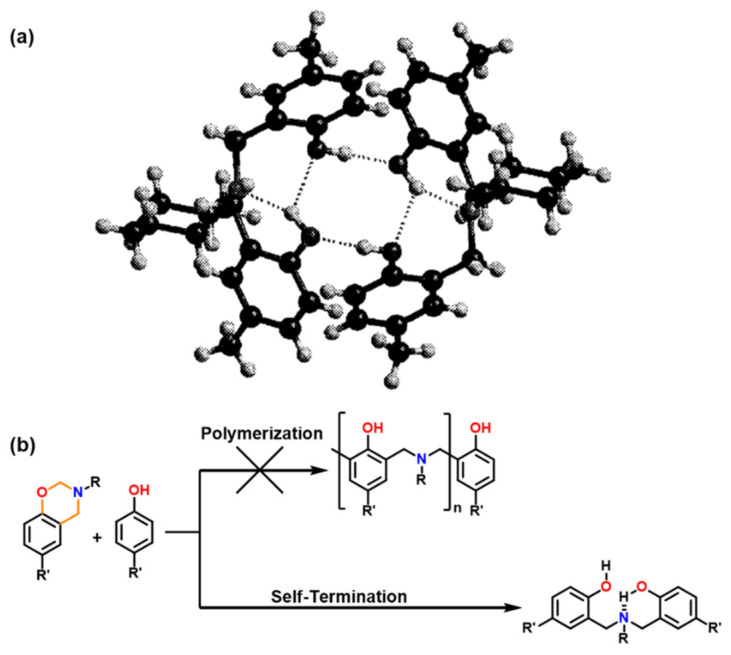
(**a**) Dimer 1 packing structure [80]. Copyright 2001. Reproduced with permission from American Chemical Society. (**b**) Self-termination of ring opening reaction of *p*-substituted phenol-based Bz [132].

Intermolecular H-bonding was found to influence both initiation and acceleration of ring-opening reaction [261]. H-bonding was also found to have negative influence and decelerate chain propagation reaction. The formation of benzoxazine dimer acts as a “self-selective reaction” due to reaction on only one site of phenol, owing to the existence of intramolecular hydrogen bonds as supported by XRD analysis, as illustrated in Figure 56a [80]. The asymmetric molecule thus formed deactivates the other phenolic-OH reactive site due to strong intramolecular hydrogen bond. A similar obstructive behavior due to intramolecular hydrogen bond leads to self-termination as shown in Figure 56b of ring-opening reaction of *p*-substituted benzoxazines is reported [132]. On the contrary, close vicinity of *o*-methylol group in PH-a assist ROP via inter-molecular H-bonding [353].

The H-bonding between the hydroxyl groups of polybenzoxazine influences physical crosslinks due to extension of intermolecular H-bonding with the polar functionalities (carbonyl, ether, amine, hydroxyl) present in other polymers such as poly(ɛ-caprolactone) (PCL) [354,355,356], poly(ethylene oxide), [357,358] chitosan (CS) [359,360,361,362], amino-cellulose [363] and polyurethane [364,365,366,367]. Although, the effect of OH groups on the polymerization of –OH or –NH_2_ containing benzoxazine monomers, non-benzoxazine monomers and polymers is not explicitly stated. However, this intermolecular H-bonding between polar groups of polymers and oxazine ring catalyzes the ring-opening kinetics and is expected to typically reduce the polymerization exotherm temperature in DSC thermograms.

**Figure 57:**
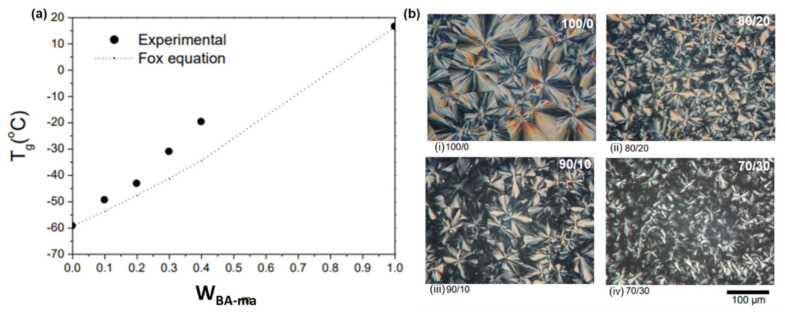
(**a**) *T*_g_-composition dependence of PCL/BA−ma blends, (**b**) spherulitic morphologies of PCL/BA-ma blends under POM crystallized at *T*_c_ = 35 °C at different ratios (100/0, 90/10, 80/20, 70/30) [369]. Copyright 2013. Reproduced with permission from Walter de Gruyter GmbH.

These intermolecular specific interactions influence the chain structure of main polymer backbone and affect microstructure morphology leading to variation in thermal and mechanical properties. A controlled nanoscale microphase separation is observed in a copolymer PBz and PCL. As shift in *T*_m_ value of PCL in blend to higher temperature suggested development of physical crosslinks between the two, which hinders the transfer of heat to the crystalline region of PCL [368]. PCL/PBz blends revealed a single *T*_g_ which supports miscibility of the two in the melt state as shown in Figure 57a [356,368]. Figure 57b shows a segregation of PCL spherulitic morphologies and an effect on its growth rate is observed which confirms a reduction in their chain mobility and dilution of PCL domains due to the interpenetrating PBz framework [369]. The polymer blends appeared to be homogeneous at the 40–70 nm scale but heterogeneous at the 2–4 nm scale as supported by ^13^C CP/MAS NMR spectroscopic analyses [370]. Instead of physical blend, naphthoxazine functional PCL macromonomers were synthesized using NP-ea as the coinitiator for the stannous-2-ethylhexanoate catalyzed living ring-opening polymerization of ɛ-caprolactone [371]. The flame resistance of poly(benzoxazine-co-ε-caprolactam) nanocomposites was improved by incorporation of cyclotriphosphazene fiber [372]. It is possible that H-bonding interactions between the produced hydroxyl groups of the open-ring Mannich base of the propagating species and poly(ε-caprolactone) or poly(ε-caprolactam) chains at molecular level may influence the fundamental structural changes of the polybenzoxazine crosslinked networks [354].

**Figure 58:**
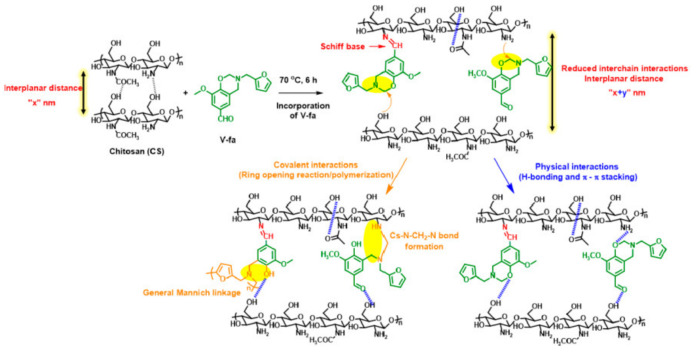
Mechanistic representation of interaction of V-fa with CS with the formation of covalent and physical linkages [361]. Copyright 2019. Reproduced with permission from American Chemical Society.

Chitosan (CS)/PBz blends revealed lowering in the ROP due to catalytic effect of amine group of CS [359,360,361]. Additionally, synergistic hydrogen bonding interactions may have role in the chain propagation in ROP reaction. The ROP temperature of V-fa was reduced remarkably to 70 °C from 204 °C when blended with CS due to Schiff base formation [361]. A probable existence of physical and chemical crosslinks network is supported, as shown in Figure 58. Among naturally occurring amine sources, amino acids, dopamine and mussel-inspired catechol-derivatives hold great potential in benzoxazine chemistry. A few other animal sourced amine or phenolic compounds that were used for making benzoxazines are catechol [100], dopamine [114] and amino acids [100,373,374].

#### 3.3.2. Participation of the Non-Oxazine Functional Group on the Polybenzoxazine Formation

**Figure 59:**
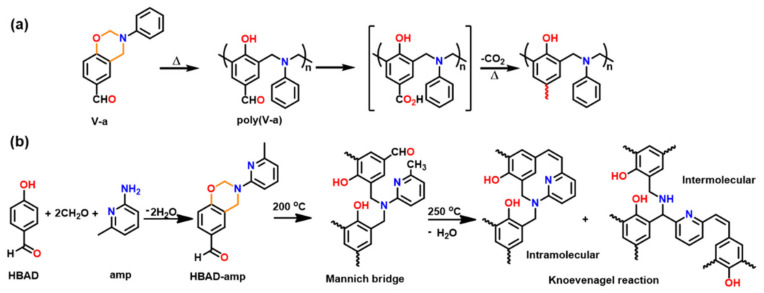
(**a**) Oxidation and decarboxylation of aldehyde groups in V-a [96]. (**b**) Synthesis of HBAD-amp and thermal mediated ring-opening polymerization and Knoevenagel reaction [376].

The existence of certain functionalities participates in the polybenzoxazine formation. The aldehyde group in V-a monomer undergoes in situ oxidation to carboxylic acid during polymerization, Figure 59a. The so-formed carboxylic acid group, like in *p*HBA-a [96,293,375], assists acid mediated polymerization of V-a and undergoes decarboxylation upon further heating and thus provides additional crosslinking sites for the growth of polymer framework.

Knoevenagel reaction [377,378] is a well-known synthetic method to form C=C group from dehydration reaction between aldehyde and methyl functionality. In HBAD-amp, electron-withdrawing character of -CHO group lowered the ROP temperature (*T*_o_ reduced to 208 °C from 240 °C) to the one without it. Heating the resultant polymer to higher temperature suppressed the formation of styrylpyridine-containing polybenzoxazine. This leads to a remarkably enhanced thermal stability due to occurrence of the intra- or inter-molecular Knoevenagel condensation reactions between benzaldehyde and methylpyridine groups as shown in Figure 59b, as indicated by FTIR studies [376].

#### 3.3.3. Influence by Molecular Alignment or Packing

**Figure 60:**
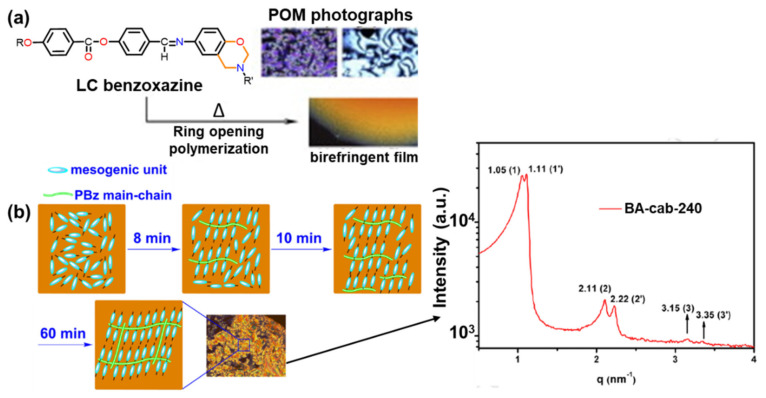
(**a**) A Schiff base based LC benzoxazine polymer with the corresponding POM image [382]. Copyright 2011. Reproduced with permission from Elsevier Ltd. (**b**) Development of liquid crystalline phases during isothermal curing and SAXS profile of BA-cab polymerized at 240 °C [383]. Copyright 2018. Reproduced with permission from Elsevier Ltd.

Introduction of Bz functionality in the side chain of polymer backbone imparted characteristic properties on the resultant molecules. Cationic ROP occurred at a lower temperature with a faster polymerization rate than ordinary benzoxazine resins in liquid crystalline (LC) benzoxazine resins [379]. Main-chain type linear benzoxaine polymers containing diacetylene and oxazine groups showed unusual polymerization behavior with the exotherm maximum below 200 °C and onset at 125 °C. It was suspected that the rigid diacetylene moieties in the polymer backbone favored local chain ordering beyond the melting temperature leading to lowered oxazine ring-opening polymerization temperature [380]. Faster polymerization (as low as ca. 110 °C) is observed even above the liquid crystal forming temperature. It is possible that the rigid chromophores maintain local order above the liquid crystal transition temperature, making it easier to polymerize. Birefringent polybenzoxazine film based on mono-oxazine structure was obtained; however, it suffers from dissolution issues due to low molecular weight of polymer [381]. Kawauchi et al. [382] reported with lower percentage conversion (~40%), the polymer film treated at 160 °C followed by cooling showed birefringence, indicating that regain of ordering at molecular level was due to reversibility in thermoresponsive phase transition, Figure 60a. The poly(BA-cab) exhibits a liquid crystalline phase after ROP of BA-cab at 240 °C. This may be due to the orderly stacking and arrangement of the mesogens favored by strong short-range interaction and availability of cholesterol-based mesogens in the side-chain rather than the backbone, as indicated from SAXS spectrum in Figure 60b. The crosslinked liquid crystalline PBz reveals a very high thermal conductivity [383].

## 4. Acceleration of the Rate of Polymerization via Intramolecular Interaction

### 4.1. Modification of Monomer Structures by Electron-Donating or -Withdrawing Groups

Structure of the mono-oxazine ring was shown to be a distorted semichair with ring strain. This strain, resulting from distorted molecular conformation, makes it possible for an oxazine ring to undergo ROP under favorable conditions. To affect ROP reaction, other than the electronegativity of oxazine ring (electron rich nitrogen and oxygen), free *o*-position of the benzene ring with respect to phenolic-OH was shown to be necessary towards thermal polymerization of monomer with or without catalyst in the temperature range 150–230 °C. In the presence of cationic initiator, propagation of polymerization reaction can proceed by reacting at unobstructed *o*-position of benzene ring to produce Mannich base phenolic type polymers. In this case, monomer propagates via formation of carbocation which is stabilized by intramolecular H-bonding and leads to high molecular weight polymer formation [267]. The conversion at maximum polymerization rate was found to be independent of the polymerization temperature. Study of the polymerization, especially kinetics of thermoset material, is necessary for determining its practical usage [384]. However, a comprehensive study of processing conditions and chemo-rheological behaviors, such as determination of time-temperature-transformation (TTT) diagram [385], has not yet been reported.

**Figure 61:**
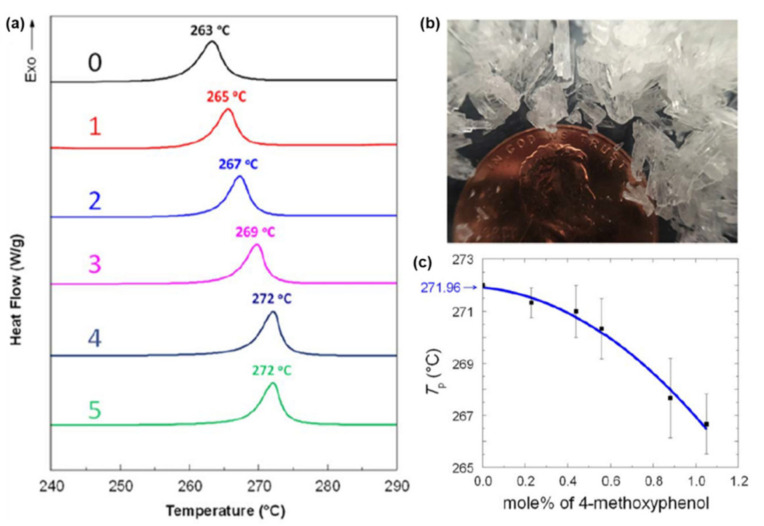
(**a**) DSC thermograms showing the change in the polymerization temperature of PH-a after successive recrystallization processes. (**b**) digital image of crystals of PH-a after all recrystallization processes and (**c**) variation of *T*_p_ as a function of the concentration (C) of 4-methoxyphenol, as the initiator (a quantitative relationship, *T*_o_ = 271.96 − 0.78C − 4.21C^2^ is obtained) [121]. Copyright 2017. Reproduced with permission from Wiley Periodicals, Inc.

The purity, type, position, number and nature of substituents on the benzene ring of phenol and/or amine and oxazine ring, chemical reactants, catalyst used and the polymerization conditions influence ROP and, consequently, the structure of polymer [82,138,292,375]. With increase in purity of benzoxazine, polymerization temperature increases. Han et al. [121] provided an experimental evidence to support the intrinsic ROP without an added initiator using a high purity monomer, as shown in Figure 61. Unlike initiator driven polymerization where *T*_p_ is expected to increase substantially towards zero initiator concentration, the *T*_p_ observed approached an asymptotic value near zero initiator concentration, supporting the hypothesis that oxazine ring can undergo intrinsic self-initiating ring-opening polymerization. The presence of impurities or catalysts decreases the polymerization temperature but not necessarily required to initiate the polymerization. This phenomenon was termed as thermally accelerated polymerization. 

The electronic character of substituents has great influences on the kinetics of polymerization of monomer. Electron-withdrawing groups promote the thermally accelerated ROP with a reduction in *E*_a_ by increasing the bond length and lowering the bond energy of C-O on an oxazine ring. Electron withdrawing groups at the *p*-position of both aromatic rings of PH-a, viz., nitro, formyl, chlorine and carboxylic, methacrylol, coumarin, etc., affect polymerization temperature [11,138,273,292,323].

With the increase in the electron-withdrawing nature of the substituents, the polymerization temperature decreases with the concomitant increase in Δ*H* values [97,210,228,375,386]. This effect is due to the generation of more acidic phenolic species as compared to unsubstituted monomer thereby increasing the catalytic activity. Furthermore, it is supported by reduced catalytic activity as indicated by an increase in the polymerization temperature of *p*-substituted photopolymerized methacrylol PH-a from 203 to 222 °C [228]. With -COOH, regardless of the position occupied, benzoxazine monomers were found to polymerize just after melting at much lower temperature. This behavior is attributed to the more pronounced effect that the acidic nature of the -COOH group has rather than its electronic effect. The -COOH catalyzes the reaction by increasing the concentration of oxonium species in the polymerizing medium. PH-a without any substitution showed *T*_p_ exotherm at 262 °C. On substituting *p*-position of both phenol and aniline moiety with –COOH, -OH group *T*_p_ decreased to 208 °C [11,292] as supported through polymerization kinetics and modelling studies. The opposite effect was observed when electron-withdrawing substituents were attached to the *p*-position of phenyl or aniline ring. A decrease in *T*_p_ is observed when an electron-withdrawing group (NO_2_, Cl) is present at *p*- to the phenyl ring while their presence at position *p*- to aniline ring increases the *T*_p_. This effect is attributed to the destabilization of phenoxide ion by electron-withdrawing substituents at *p*-position of phenyl ring. In the case of electron donor substituents such as methoxy and methyl group, no notable effect on the rate of polymerization was observed irrespective of its presence in phenyl or aniline ring [138]. The only exception to this is the substitution by -OH/NH_2_ group in phenyl ring [110,322]. The amine and ammonium groups were found to affect the occurrence of benzoxazine polymerization at low temperature [273,323,336,359,361,387]. The basicity of N atom affects the rate of oligomer formation and thus polymerization [140,388]. Surprisingly, presence of electron withdrawing acetylene--functional group in aniline at the *m*-position lowered the *T*_p_ by 36 °C as compared to its structure without acetylene group [389]. Both PH-fa and BA-fa showed a decrease of 14 and 20 °C, respectively, in ROP temperature as compared to PH-a and BA-a supporting the suggested role played by amine component is correct. Furan ring of furfuryl amine undergoes electrophilic aromatic substitution reactions more readily than a benzene ring of aniline [130]. The presence of fluorine at the *meta*-position to phenol ring facilitated both polymerization and crosslinking reactions, which is accounted to its smaller size and electron withdrawing nature [390]. The existence of phenolic-OH [391,392,393,394,395], phenyl thioether and hydroxyl [104,282,396,397] moieties lowers the polymerization temperature. Besides DSC plots, polymerization of benzoxazines can even be monitored by time-conversion plots at variable temperature [282], where either the conversion of monomer or formation of polymer can be monitored by various techniques. It was found that processing at low temperature, followed by heating at high temperature, interconverts the nature of linkages, i.e., induces conversion of labile to thermally stable crosslinking of network, with improved thermal properties.

Similar results were obtained when bridging electron-withdrawing groups present in diamine 4,4′-diaminodiphenylmethane (ddm) and 4,4′-diaminodiphenylether (dde). The ddm based Bz showed 10 °C lower *T*_o_ than dde based monomers. However, their counterparts containing methylol groups (named *o*MeOPH-dde and *o*MeOPH-ddm, respectively) at different positions plays a significant role in accelerating polymerization due to intramolecular assistance [398].

Similarly, even structural isomers in amine component with position of polar ether linkages (*p*- vs. *m*-), a prominence of electronic effects in polymerization temperature is obvious. A lowering in *T*_o_ value with biphenyl ether linkage at *p*- vs. *m*-position in diamine suggesting an interplay of electronic effects [399]. 

**Figure 62:**
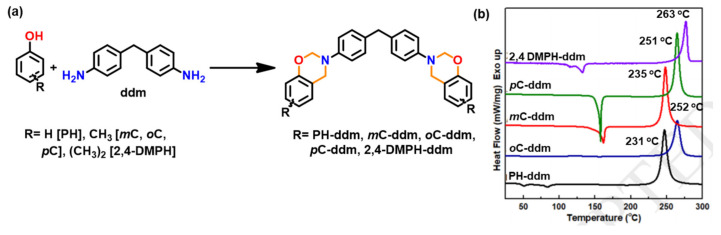
(**a**) Methyl substituted bis-benzoxazine monomers [167]. (**b**) DSC thermograms of aromatic diamine-based benzoxazine monomers at a heating rate of 10 °C min^–1^ [167]. Copyright 2018. Reproduced with permission from Elsevier B. V.

As compared to PH-ddm, no significant change in *T*_p_ is observed when methyl groups are present on phenol (*o/m/p*C, PH, 2,4-DMPH) rather on amine (ddm) in bis-benzoxazine as shown in Figure 62a. However, *o*- and *p*-blocked phenol based monomers showed a substantial increase in *T*_p_ as evident from DSC trace in Figure 62b [167].

**Figure 63:**
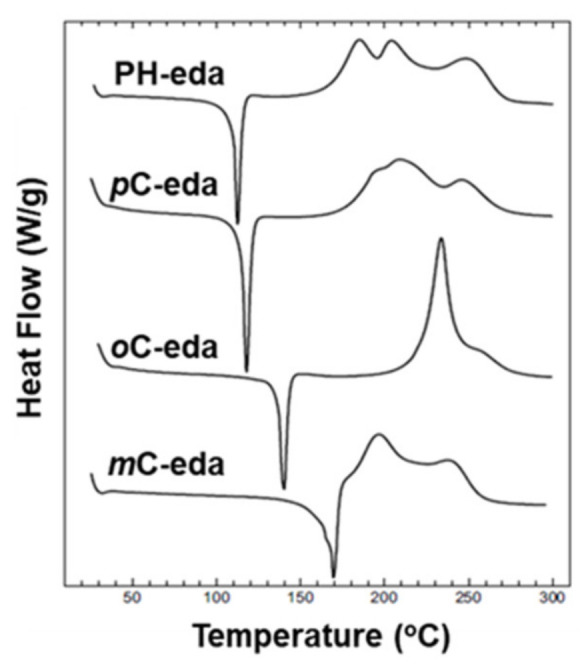
DSC thermograms of unsubstituted and methyl substituted phenol monomers based on ethylenediamine (eda) [137]. Copyright 2006 and 2008. Reproduced with permission from Elsevier Ltd.

A variation in diamine component from aromatic to aliphatic also affected the polymerization temperature. Allen et al. [137] investigated the effect of aliphatic diamine chain length and relative position of methyl substituent in phenol ring in benzoxazine monomer. Methyl substitution in phenol decreased the reactivity of the monomer and caused the position of the polymerization exotherm to increase to higher temperatures, as can be seen from Figure 63. Polymerization of the *o*-substituted monomer is forced to proceed through less favorable *p*-position as indicated by high temperature required for polymerization. Even the temperature required to thermally activate benzoxazine polymerization directly increases as a function of diamine chain length. The dilution of oxazine functionality with non-reactive functionalities such as alkyl, alkoxy or other inactive groups, increases the polymerization temperature [116,238].

**Figure 64:**
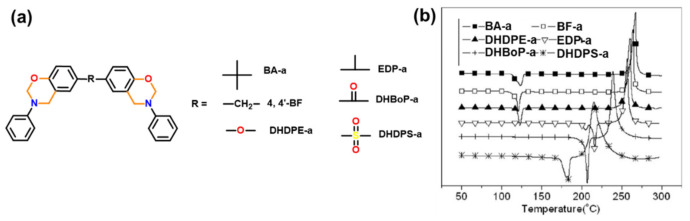
(**a**) Variation in biphenyl linkage in bisphenol based bis-benzoxazine monomers and their (**b**) DSC thermograms [400]. Copyright 2011. Reproduced with permission from Wiley Periodicals, Inc.

The electronic effects from bridging groups in bisphenol [400] or diamine [399] effects on ring-forming and ring-opening reactions have been studied using the compounds shown in Figure 64a. In general, electron-withdrawing groups present in phenol affect the condensation reaction to form the monomer as they induce electronic effect which lowers the reactivity of phenolic-OH. On the contrary, presence of such groups in phenolic ring promotes thermally accelerated polymerization due to the stabilization of the ring opened intermediate ion. The electronic effect due to various bridging groups such as >C(CH_3_)_2_, >CH_2_, >O, >CO, and >SO_2_ in bisphenol-A was studied. A variation in position of ether linkage, *p*- vs *m*- in the aromatic diamine found to lower the ROP temperature. Especially, stronger electron-withdrawing groups revealed predominance of arylamine methylene Mannich bridge structure in the polybenzoxazine network. Figure 64b shows that the value of *T*_p_ varies in the order BA-a < BF-a < DHDPE-a < EDP-a < DHBoP-a < DHDPS-a as predicted by electron donating or withdrawing nature of bridging groups [400].

Compared with BA-a, the bis-benzoxazine containing Schiff base linkages forms aligned chain segments requiring higher energy to facilitate the polymerization due to π–π stacking. This restricts the motion of oxazine rings, leading to less involvement of oxazine rings in polymerization and, therefore, resulting in lower Δ*H* values [192]. Incorporation of rigid spacer units, trimethylphenyl indane and tetramethyl spirobiindane between the reactive benzoxazine units, leads to mobility restriction imposed on the reactive sites by the increasing viscosity of the polymerizing medium, affecting the polymerization rate [401]. The effect of intermolecular H-bonding between the urea linkages allowed close proximity of oxazine rings, which in turn, lead to accelerated polymerization at much lower temperature [225].

Introduction of fluorenyl unit as bridging group in bis-amine or bis-phenol similar to BA-a did not show noticeable effect in affecting ring-opening temperature, suggesting non-participation of such functionality electronically in affecting intermediate stability [154,157]. Interestingly, certain functionalities in the monomer such as diethylphosphonate groups undergoes thermal dissociation to form acidic phosphite groups which catalyze the ROP as reflected with lower value of *E*_a_ [17].

Various structural isomers of bisphenol-F based benzoxazine isomers (2,2′, 2,4′ and 4,4′ substituted) showed rate of polymerization in the order 4,4′- < 2,4′- < 2,2′- isomer [152]. This trend is the opposite of all other non-benzoxazine polymers reported in the literature. Resorcinol based bis-oxazine monomer exhibited an exothermic *T*_p_ value 179 °C, much lower than 245 °C of BA-a [402]. 

Instead of introduction of functionalities in the phenol or amine group involved directly in the formation of benzoxazine ring, Bz ROP is also sensitive to when functionality is varied at the other positions. For instance, presence of electron withdrawing end-caps attached to the imide ring present at *ortho-*position to the phenol ring in Bz monomer also lower the polymerization temperature [202].

### 4.2. Design of Monomer Structure to Influence the Intermolecular Packing (Rigid Groups)

Over the time, to improve crosslinking density and hence *T*_g_ of polybenzoxazine, monomers containing multiple oxazine rings are synthesized using multifunctional phenol/amine as a starting material. With each increase in the number of oxazine rings, polymerization temperature reduces substantially. This lowering in temperature is attributed to the presence of intramolecular cross-interaction between oxazine ring and aromatic hydrogens as indicated by the respective ^1^H-^1^H NOESY NMR spectra of benzoxazine monomers. Another advantage observed was the decrease in the weight loss during curing (from 90% to 2.4%) with increase in *T*_g_ [147]. Introduction of the two oxazine functionality onto single benzene reduced the polymerization temperature by 62 °C. Incorporation of the third oxazine functionality was not much effective and ROP temperature was reduced only by 13 °C as its phenolic ring is completely substituted and has no free reactive position [144].

**Figure 65:**
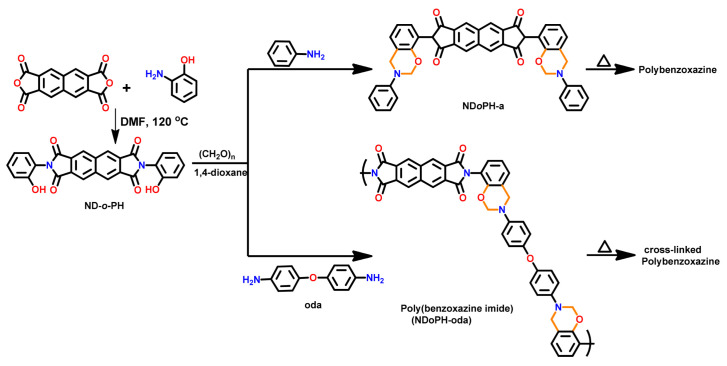
Synthesis of *o*-imide functional bisbenzoxazine monomer and main-chain type polybenzoxazine oligomers [244].

NTCDA was chosen to prepare indole-containing benzoxazine due to its rigid structure, which helped in decreasing the ring-opening temperature of dianhydride ring during its reaction with amines as shown in Figure 65 [244].

**Figure 66:**
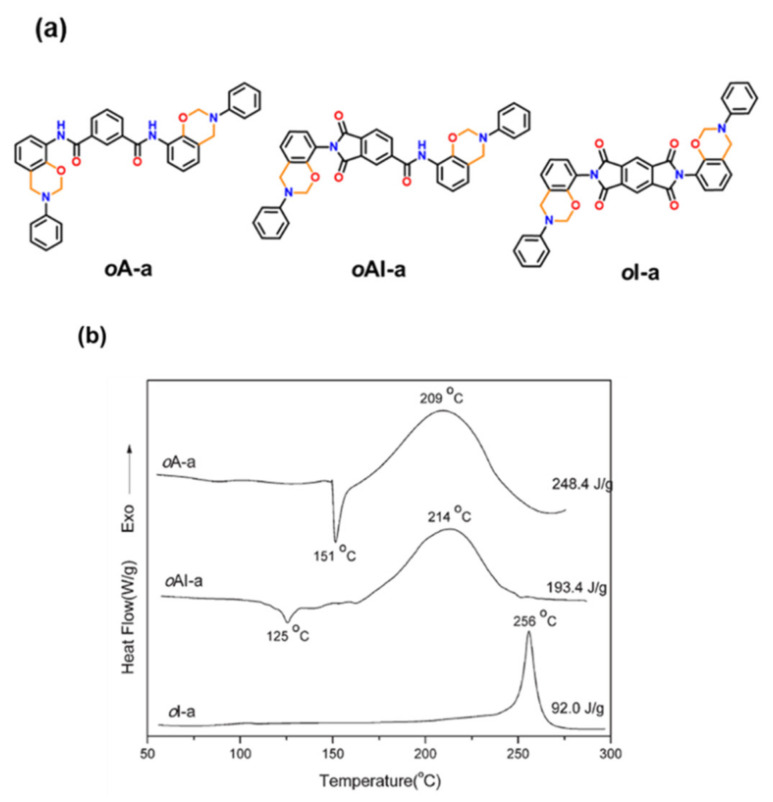
(**a**) Smart benzoxazine monomers having bis-*o*-amide, asymmetric *o*-amide-imide and *o*-bisimide groups and their (**b**) DSC traces [197]. Copyright 2015. Reproduced with permission from Royal Society of Chemistry.

The conversion from polybenzoxazine to polybenzoxazole accompanies water or CO_2_ formation when the precursor is *o*-amide or *o*-imide functional benzoxazine, respectively. While the amount of water produced is relatively small, it is nonetheless desirable to avoid small molecular compound formation in a short period to avoid potential void formation during processing. Taking advantage of different temperature for water and CO_2_ formation of the *o*-amide and *o*-imide functional benzoxazines, Zhang et al. [197] synthesized an asymmetric bisoxazine molecule having both *o*-amide and *o*-imide groups as shown in Figure 66a. Indeed, the volatile formation at each transition nearly halved. The maxima of ROP of *o*-(amide-imide) Bz monomer (oAI-a) lies in between *o*-amide (*o*A-a) and *o*-imide (oI-a) Bz monomer, as evident from Figure 66b, exhibiting melting point at 125 °C, whereas *o*-imide monomer polymerized without melting resulted in low degree of crosslinking and low heat of polymerization, which is due to highest rigidity *o*I-a. Unexpectedly, it was found that *o*-(amide-imide) polybenzoxazine has the highest thermal stability. This class of polymers exhibit unusual thermomechanical properties in that G′ is nearly constant in a wide temperature range from room temperature to 400 °C.

### 4.3. Influence of Oxazine Ring-Opening by Intramolecular Interactions: Neighboring Group Effect and 5- or 6-Membered Ring H-Bonding

**Figure 67:**
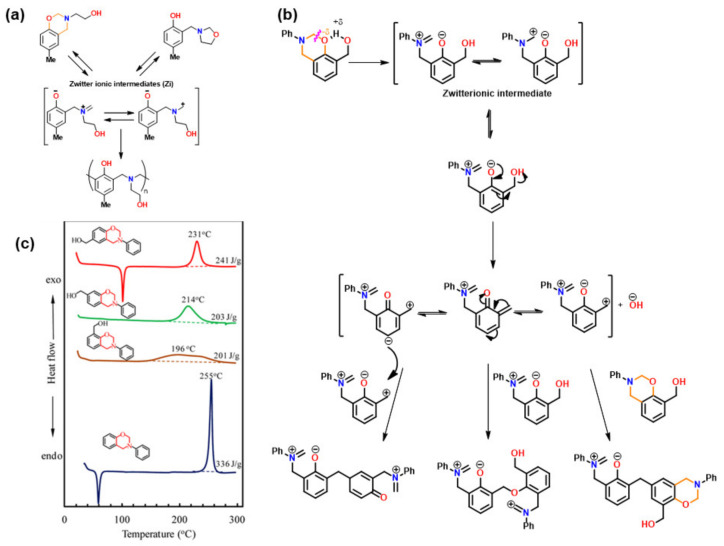
Acceleration mechanism of oxazine polymerization by the neighboring group effect. Variation in mode of polymerization of benzoxazine monomer with substitutions: (**a**) 2-hydroxyethyl [189] and (**b**) 2-hydroxymethyl group [403]. (**c**) DSC thermogram of benzoxazine monomers with and without methylol functionality [403]. Copyright 2012. Reproduced with permission from American Chemical Society.

Neighboring group participation of hydroxyl group in ROP of oxazine ring in a substituted phenol-based vs. unsubstituted or suitably modified benzoxazine (control) has been studied [189,398,403]. Kiskan et al. [190,217] synthesized hydroxyethyl terminated ether chain-functional benzoxazine monomers and found a reduction in polymerization temperature as compared to traditional unfunctionalized benzoxazine monomer. Kudoh et al. [189] extended the mechanistic knowledge of the polymerization reaction to elaborate the role of the hydroxyethyl functionality in activating the ring-opening reaction of the *N*-(2-hydroxyethyl)-1,3-benzoxazine monomer. *N*-(2-hydroxyethyl)-1,3-benzoxazine was found to polymerize at a much faster rate than non-polar structural analogue. This is accounted to the intramolecular reaction of hydroxyl groups with cationic moieties of the zwitterionic intermediate formed by the ring-opening reaction of benzoxazine and in situ generated a 5-membered cyclic *N,O*-acetal structure as illustrated in Figure 67a. Sudo et al. [404] reported utility of amino acid, namely, glycine and β-alanine as amine component (as a tetrabutylammonium salt) with *p*-cresol to form mono-oxazine monomer. The rate of polymerization is faster for glycine (with 100% conversion within 5 h at 120 °C) followed by alanine and then 2-aminoethanol followed by methylamine based benzoxazine. The reason attributed for such a fast polymerization rate is the neighboring group participation of carboxylate ion and the effect that polarity of the solvent and counter ion has. An increase in steric bulk to the substituent *N*-alkyl group of benzoxazine monomer led to a decrease in the polymerization rate [405]. This is accounted to the release of volatile *N*-alkylimine compound as a byproduct in bulk amount. Replacement of *N*-propyl to *N*-allyl functionality in benzoxazine exhibited a faster polymerization rate. This is again due to the stability imparted by the intramolecular interaction between the cationic species and the π-bond of the allyl group [406].

Altering 2-hydroxyethyl to 2-hydroxymethyl, i.e., *o*-/*m*-/*p*-methylol-substituted benzoxazines found to accelerate the rate of polymerization as compared to PH-a. Baqar et al. [403] studied the effect of methylol group at *o*-, *p*- and *m*-position of phenolic ring on polymerization as shown in Figure 67b,c. The heat of polymerization and *T*_p_ for the methylol substituted monomers is lower than the control, PH-a. This suggests the co-occurrence of ROP and the release of water as a byproduct from the polymerization. This also posits the electronic and intramolecular H-bonding interactions between methylol and oxygen in the oxazine ring and thus activating the ring to open and polymerize at low temperature. The effect is more pronounced in *o*-methylol, which is attributed to the resonance of the benzoxazine ring being affected by the methylol to form the intramolecular 6-membered hydrogen bonding, as proposed in Figure 67b. Methylol monomer exhibits lower *E*_a_ compared to the unfunctionalized monomer [407].

**Figure 68:**
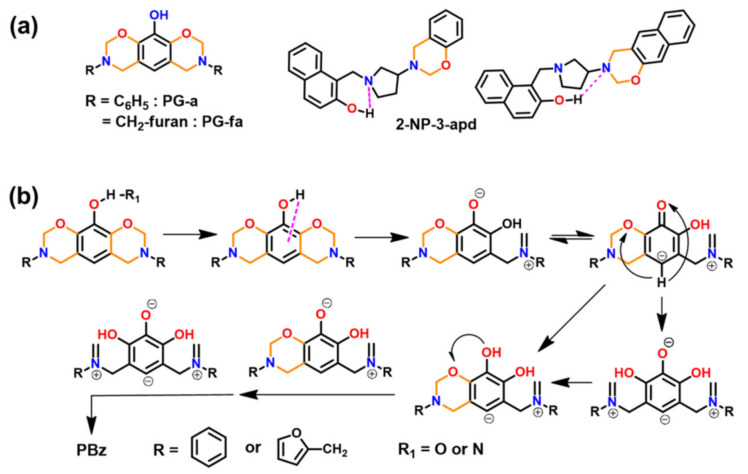
(**a**) Existence of polar phenolic-OH in benzoxazine monomer assisting ROP reaction. (**b**) Proposed structure evolution of PG-a and PG-fa during the ROP process [333,408].

Similarly, existence of phenolic-OH group in pyrogallol based benzoxazines PG-fa and PG-a and naphthoxazine revealed interesting H-bonding interactions, Figure 68. Low monomer loss due to evaporation is observed as ensured by stable intramolecular H-bonds with nitrogen atoms in napthoxazine. The -OH interacts with the N in the pyrrolidine ring and oxazine ring through stable intramolecular hydrogen bonds instead of presenting free -OH at room temperature, leading to the enhanced shelf life of the monomer. The free phenolic -OH initiates and catalyzes the polymerization. A latent catalytic effect is observed in 2-NP-3-apd [333]. Later temperature dependent effect of H-bonds on ROP was studied with the help of interaction of free phenolic hydroxyl between two oxazine rings attached to the same benzene ring as in pyrogallol. Interestingly, the unprotected hydroxyl group accelerates the cleavage of oxazine ring, thereby promoting ROP reaction. The intermolecular hydrogen bonding -OH⋯N (of another benzoxazine molecule R1) changed preferentially to the -OH⋯π intramolecular hydrogen bonding OH….π-electron of the benzene ring as shown in Figure 68b. As a result of these interactions, it may be anticipated that PG based benzoxazine monomer may show a lower *T_p_* and *E*_a_ than 2-NP-3-apd [408].

#### 4.3.1. Background of Intramolecular H-bonding in Benzoxazines

**Figure 69:**
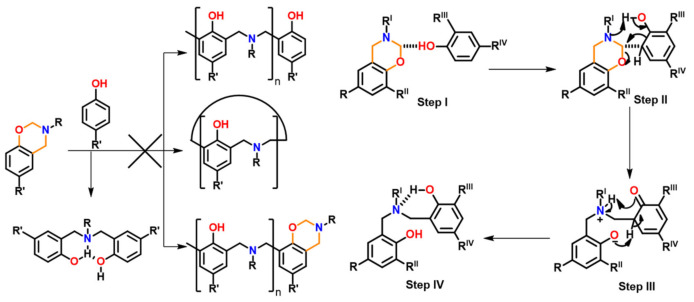
Hypothesis of the molecular mechanism for self-termination of a *p*-substituted phenol-based benzoxazine [132].

The structure of inter- and intra-molecular H-bonding occurring in benzoxazine dimers, trimers and tetramers was studied by high-resolution solid-state ^1^H NMR. The 2D single quantum-double quantum spectroscopic results provided evidence for local helical formation trend in polybenzoxazine chains despite the polymer being overall amorphous [409]. The persistence of intramolecular hydrogen bonding does exist even in the molten state [125]. It was proposed that benzoxazine with free *o*-position in phenol or two free hydroxyl groups in an open benzoxazine ring reveals co-occurrence of both inter- and intra-molecular H-bond between –OH---N–, depending on the pK_a_ value of the involved species. This led to the formation of asymmetric product due to preoccupancy of such van der Waals forces. Therefore the *p*-substituted phenol-based monomers undergo self-terminated ROP as soon as dimer forms instead of giving linear oligomer or polymer, with varied reaction conditions as supported by TLC, HPLC, FTIR, ^1^H-NMR and elemental analysis [80,132]. This feature is illustrated in Figure 69.

Intramolecular hydrogen bonds are the basis for ring-shape and helical conformations of trimeric and tetrameric units of Bz. Hydrogen bonds strongly influence the adopted structural conformation. Both trimers and tetramers contained stable intramolecular OH---N and usual OH---O hydrogen bonds giving them a closed-ring like geometry [409].

The -OH---N intramolecular hydrogen bonding forms a stable six-membered structure, resulting in a methylene proton deshielding effect in methylene protons. The amine functional group in the Mannich bridge is greatly responsible for the distribution of hydrogen bonding species. The strength of hydrogen bonding is dependent on the electronegativity of the side group or the basicity of amine functional group which is attached to the nitrogen atom [265,410]. Therefore, BA-m mainly consists of -OH---N intramolecular hydrogen bonding while BA-a has a large amount of intermolecular hydrogen bonding and relatively weak hydrogen bonding groups in the polymer network structure. The various proposed cyclic structures of *N*,*N*-bis(3,5-dimethyl-2-hydroxybenzyl)methylamine dimers showed different types of H-bonding interactions to various extents by FTIR analysis as tabulated in Table 5 [411], which are consistent with XRD structures of methyl-dimer [125].

**Figure 70:**
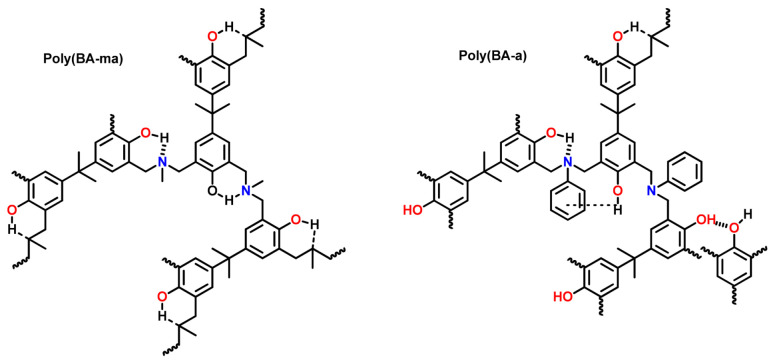
Probable H-bonded network structure in PBzs with variations in the nature of amine. [265].

The extent and nature of hydrogen bonding network is closely related to the basicity of the amine constituent [410]. A weaker amine, aniline (a) based poly(BA-a) showed great OH...N intermolecular hydrogen bonding while the remainder was intramolecular hydrogen bonding as shown in Figure 70. On the contrary, a monomer based on a stronger amine, methylamine (ma), poly(BA-ma) revealed a dominance of intramolecular hydrogen bonding interactions [265]. Besides basicity of amine, an interplay of the proton-transfer equilibrium between HO····N ⟷ O^−^····H⁺N in the Mannich bridge (as >NMe or >NPh) is also accounted to the variable distribution of hydrogen bonding networks. As a result of above interactions, the polarizability of the proton is shifted depending upon the nature of amine; it is found more towards the HO····N in case of aniline-based model compounds.

#### 4.3.2. Smart Benzoxazines

In general, external addition of initiators/catalysts suffer from thermal instability, limited shelf life and time-dependent leaching out of the polymer matrix. Furthermore, certain niche applications demand absence of such aids. Such initiators and proton donors can be incorporated into either the monomer or polymeric structures. This covalent tethering of initiator functionalities overcomes the issues of high volatility and migration rates often associated with low-molecular weight initiators. The covalent bond between initiator and monomers acts as “equivalent initiator monomers” [11,292,392,412] and inherent functionalities which mediates ring-opening reactions inter-molecularly [379,380,413] and intra-molecularly [189,414].

**Figure 71:**
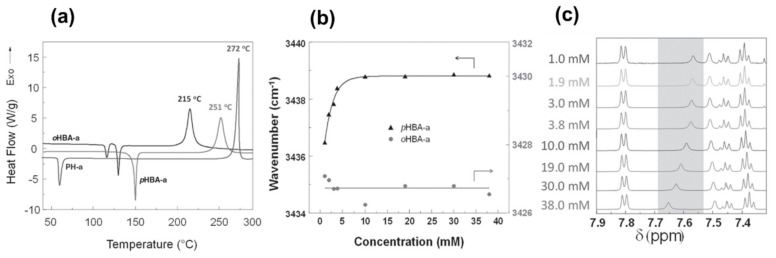
Effect of inter- and intramolecular H-bonding interactions in *o*HBA-a and *p*HBA-a vs PH-a: (**a**) DSC thermograms. (**b**) Variation in N-H stretching wavenumber vs concentration. (**c**) ^1^H NMR spectra of *p*HBA-a at different concentrations (recorded at 25 °C using CDCl_3_ as a solvent) [243]. Copyright 2017. Reproduced with permission from WILEY-VCH Verlag GmbH & Co. KGaA, Weinheim, Germany.

Figure 71a compares the DSC thermograms of mono-oxazine benzoxazine with and without the intramolecular five-membered ring H-bonding, showing dramatic influence on the polymerization exotherm temperature, supporting catalyzing effect of intramolecular H-bonding interactions [243]. The existence of intermolecular vs intramolecular five-membered-ring hydrogen bonding interactions between the NH (amide group) and the oxygen (oxazine ring) in *p*HBA-a vs. *o*HBA-a was supported by concentration independent FTIR and NMR experiments Figure 71b,c.

**Figure 72:**
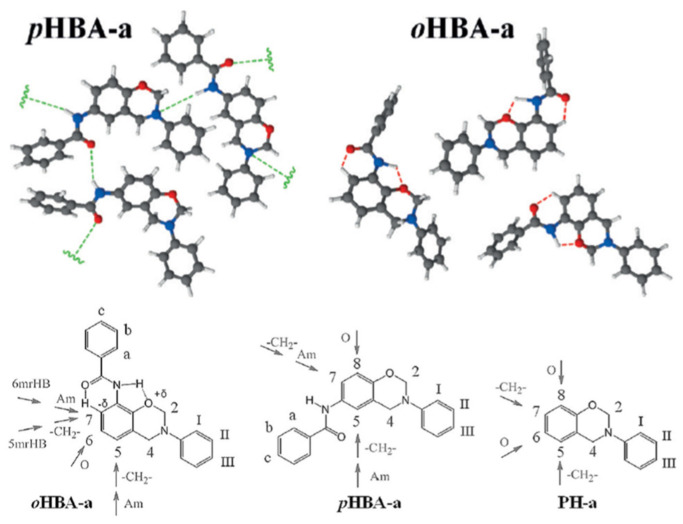
Proposed hydrogen-bonding interactions in oHBA-a and pHBA-a. Above is shown the intermolecular hydrogen bonding in both *o*HBA-a and *p*HBA-a, intramolecular in *o*HBA-a, and the influence of the amide substituent at the ortho and para positions in the benzoxazine nuclei. The arrows show the “activating action” produced by the different substituents and their effects, with Am referring to the amide group, >CH_2_ to the methylene group at the 4-position, O to the oxygen atom in the oxazine ring and 5mrHB and 6mrHB to the five- and six-membered-ring hydrogen-bonding systems, respectively [415]. Copyright 2016 and 2012, respectively. Reproduced with permission from John Wiley & Sons, Inc.

A comparison of *T*_p_ amongst all known mono-oxazine structures with *o*-amide containing benzoxazines revealed an outstanding tendency to polymerize at much lower temperatures without the use of either initiator or catalyst [193,195]. As illustrated in Figure 72, Froimowicz et al. [415] demonstrated the existence of a five-membered ring intramolecular hydrogen bonding in *o-*amide isomer (*o*HBA-a, HBA- hydroxybenzoic acid) vs. intermolecular H-bonding in *p*-amide (*p*HBA-a) benzoxazine isomer influencing *T*_p_ to vary, as 187 and 241 °C, respectively. Interestingly, the most favored reacting site for extending the polymerization is the 7-position (*m*- to phenolic-OH), unlike other conventional benzoxazines as presented in Figure 72.

## 5. Conclusions

The current review is designed and written in order to understand the importance of reagent choice in designing Bz monomers and their associated structure–property relationship, notably, polymerization characteristics. As such, the structure of Bz monomers has a strong influence on the formation of polymers and the related applications. The field is widely explored utilizing both petrobased and biobased/agrowaste origin feedstocks to form Bz monomers. Nevertheless, on one side, the utility of natural and waste origin chemicals as feedstocks for the manufacture of polybenzoxazines is steadily increasing, while on the other side, petrobased raw materials are used to structurally design new monomer structures to affect polymerization conditions and resultant properties. Advancement in lowering the polymerization temperature either by molecular tailoring at the structure level or by adding external aids and/or copolymerization with other polymer structures is still progressing on different frontiers. This progress is heavily dependent on the processability and end-use applications. Unlocking new frontiers using inherent functionalities thoughtfully may help in developing new domains of robust applications. While it is interesting and easier to exploit structure variation of phenol and amine to form the Bz structure, demand for new areas of exploration is high. Consequently, the extension of bonding characteristics, such as hydrogen bonding, in situ structural transformations, interactions other than the usual PBz linkages, etc., hold significant potential. An intense focus on expanding the scope of PBzs with high thermal stability, flexibility in the polymer backbone, low polymerization and processing temperature is needed to take research to next level. 

## Figures and Tables

**Table 1:** Thermal characterization of substituted polybenzoxazines [161].

	*T*_g_^a^ (°C)	*T*_p_ (°C)	*H* (J/g)	*T*_5%_ (°C)	*Y*_c_
BA-a	168	251	340	315	30
BA-*o*t	114	247	289	228	32
BA-*m*t	209	231	325	350	31
BA-*p*t	158	259	310	305	32
BA-35x	243	217	298	350	28

^a^: *T*_g_ values are reported from DSC data.

**Table 2:** Thermal characterization of naphthoxazine and general benzoxazine [182].

Monomer	*T*_p_ (°C)	*T*_5%_ (°C)	*T*_10%_ (°C)	*Y*_c_ at 800 °C (%)
1NP-*p*cna	206	332	359	57
2NP-*p*cna	215	311	330	47
1NP-*o*cna	172, 186	302	323	51
2NP-*o*cna	174,201	309	327	41
1NP-a [183]	156	310	341	46
2NP-a [183]	255	216	236	20
PH-a [181]	267	294	347	40

**Table 3:** Thermal characterization of *o*-(amide-co-imide) functional polybenzoxazines [198].

	*T*_g_^c^ (°C)	*T*_5%_ (°C)	*T*_10%_ (°C)	*Y*_c_ (%)
poly(BHPICA-a) ^a^	332	406	476	63
poly(BHPICA-ddm) ^a^	>400	410	473	52
poly(BHPICA-a)-400 ^b^	-	536	589	71
poly(BHPICA-ddm)-400 ^b^	-	503	555	60

Obtained after heating at 300 °C ^a^ and 400 °C ^b^ for 1 h to assist cyclodehydration reaction; ^c^ *T*_g_ values are reported from dynamic mechanical analysis.

**Table 4:** Thermal stability and degradation data from thermogravimetric analysis under nitrogen atmosphere [352].

	*T*_5%_ (°C)	*T*_i_ (°C)	*T*_50%_ (°C)	*T*_p_ (°C)	*Y*_c_ (700 °C)
BA/ECC	310	310	382	373	20
HBP-AMIM^+^PF_6_^−^-2 (1–7 wt%)	332–341	352–359	404–415	417–422	27–30
HBP-AMIM^+^PF_6_^−^-1 (3 wt%)	332	359	413	416	25
HBP-AMIM^+^PF_6_^−^-3 (3 wt%)	345	362	417	425	29

**Table 5:** Fraction of various hydrogen bonded species for different sizes of benzoxazine oligomers in 50 mM CCl_4_ solutions [411].

	Free OH	OH*-p* Intra HB	OH*-*O Intra HB	OH-N Intra HB	OH-O Inter HB
	3615 cm^−1^	3559 cm^−1^	3467 cm^−1^	3000 cm^−1^	3364 cm^−1^
Asym. Methyl-dimer	-	-	-	1.00	-
Methyl-dimer	0.09	0.14	0.11	0.53	0.13
Methyl-trimer	0.04	0.11	0.09	0.67	0.10
Methyl-tetramer	0.01	0.04	0.02	0.88	0.05

**Figure A1:**
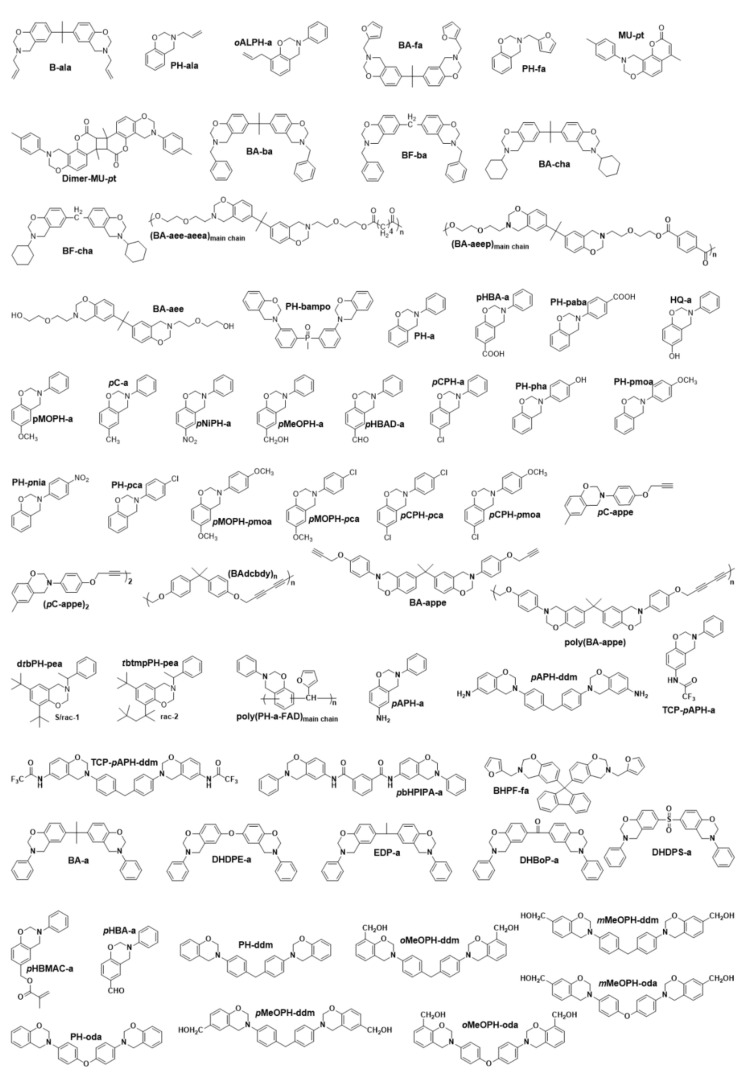

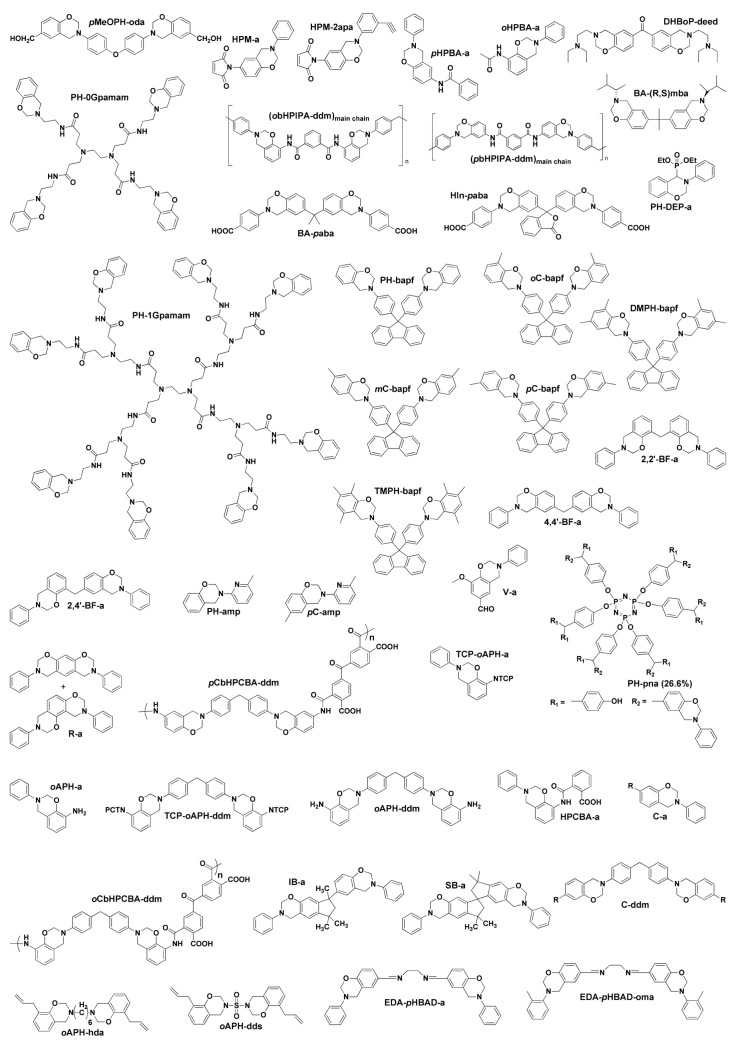

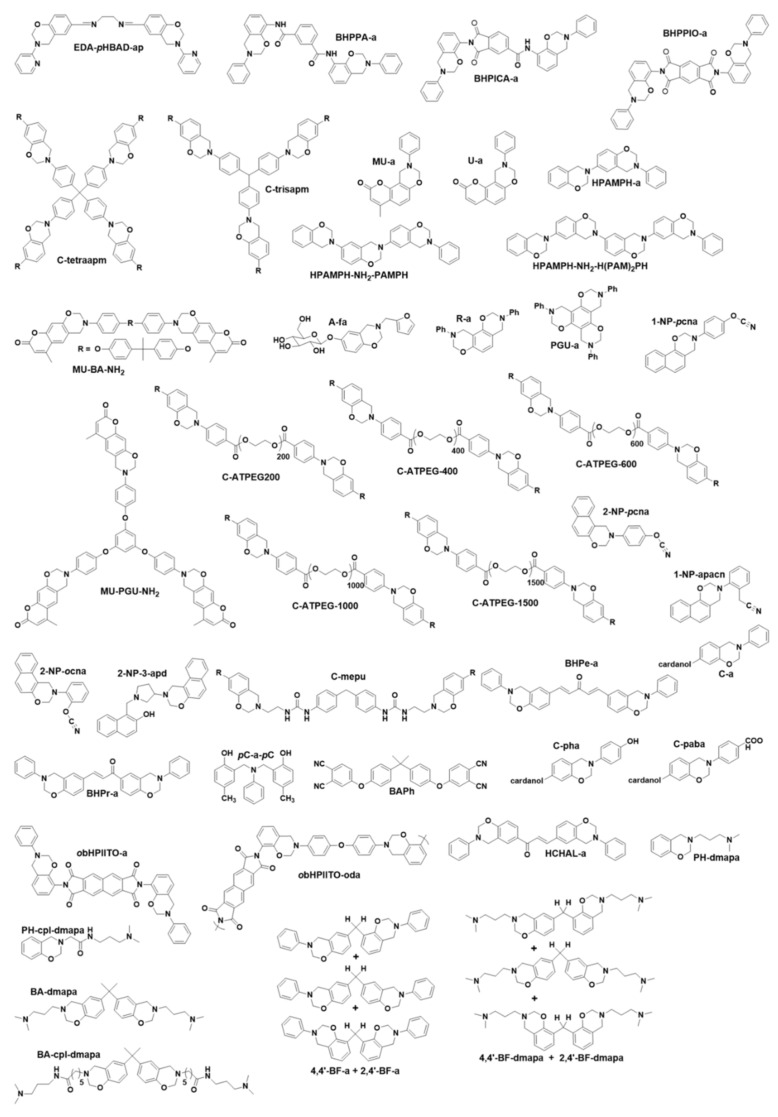

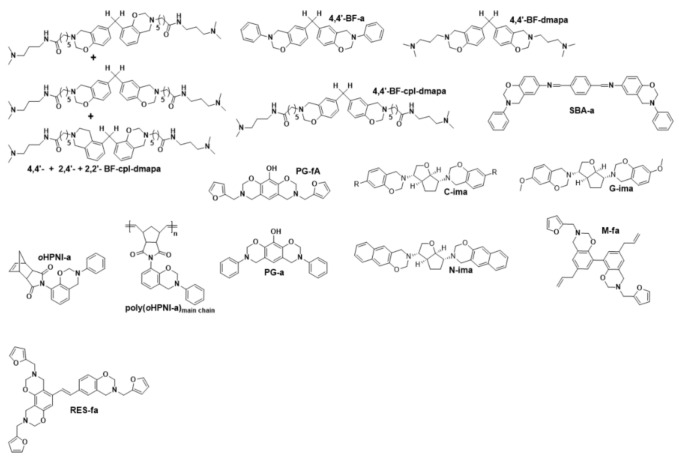
Representative structure of monomers and associated nomenclature.

## Abbreviations

0G	Zero generation
1G	First generation
35x	3,5-Xylidine
22PP	2,2′-Biphenol
α-ZrP	α-Zirconium phosphate nanoplatelets
a	Aniline
A	Arbutin
aa	Amic acid
aap	Aminoacetophenone
aba	Aminobenzoic acid
AC	Acacia catechu
acac	Acetylacetonate
aee	Aminoethoxyethanol
aeea	bis(2-(2-aminoethoxy)ethyl) adipate
aeep	bis(2-(2-aminoethoxy)ethyl) terephthalate
AEPA	α,ω-aminoligo(ethylene terephthalamide)
ala	Allylamine
ALPH	Allylphenol
AMIM^+^PF_6_	1-allyl-3-methylimidazolium hexafluorophosphate
Amp	2-Amino-6-methylpyridine
ap	2-Aminopyridine
apa	3-Aminophenylacetylene
apd	Aminopyrrolidine
APH	Aminophenol
appe	Aminophenyl propargyl ether
appn	3-Aminophenoxyl-*o*-phthalonitrile
aptes	Aminopropyltriethoxysilane
ArIFB	Diaryliodonium tetrafluoroborate
ATPEG	Amine terminated poly(ethylene glycol)
ba	Benzylamine
BA	Bisphenol A (for the sake of convenience bisphenol A is written as BA instead of BPA)
BADCY	Bisphenol A dicyanate ester
BAEPA	bis-(aminoethyl) terephthalamide
bampo	bis(*m*-aminophenyl)methylphosphine oxide
BA-NH_2_	4-(4-(2-(4-(4-aminophenoxy)phenyl)propan-2-yl) phenoxy)benzenamine
BAPBACP	3,3′-(((Propane-2,2-diylbis(4,1-phenylene))bis(oxy))bis(4,1-phenylene))bis(3,4-dihydro-2*H*-benzo[e][1,3]oxazine-6-carbonitrile)
Bapf	9,9-Bis-(4-aminophenyl)-fluorene
BAPh	Bisphenol A phthalonitrile
BEM	BA-a/DGEBA (2 wt%)/Imidazole (1.5 wt%)
BEP	Benzoxazine/Epoxy/Phenolic (6/4/1 by weight)
BF	Bisphenol F
BHPe	1,5-Bis(4-hydroxyphenyl)penta-1,4-dien-3-one
BHPr	1,3-Bis(4-hydroxyphenyl)propanone
BHPF	9,9-Bis(4-hydroxyphenyl)fluorene
BHPICA	*N*,2-bis(2-hydroxyphenyl)-1,3-dioxoisoindoline-5-carboxamide
bHPIITO	2,7-Bis(2-hydroxyphenyl)isoindolo[5,6-f]isoindole-1,3,6,8(2H,7H)-tetraone
bHPIPA	*N*^1^,*N*^3^-bis(4-hydroxyphenyl)isophthalamide
BHPPA	*N*^1^,*N*^3^-bis(2-hydroxyphenyl)isophthalamide
BHPPIO	2,6-Bis(2-hydroxyphenyl)pyrrolo[3,4-f]isoindole-1,3,5,7(2H,6H)-tetraone
BMI	4,4′-Bismaleimidodiphenylmethane
BO	Butoxyphenol
BoP	Benzophenone
BTDA	3,3′,4,4′-Benzophenonetetracarboxylic dianhydride
Bz	Benzoxazine
BZ-CN	Bisphenol A-based benzoxazine-functionalized phthalonitrile
C	Cardanol
ca	Chloroaniline
cab	Cholesteryl 4-aminobenzoate
CbHPCBA	4,4’-Carbonylbis(2-((4-hydroxyphenyl)carbamoyl)benzoicacid)
CH	Cyclohexyl
cha	Cyclohexylamine
Char yield	*Y*_c_
CHOTs	Cyclohexyl *p*-toluenesulfonate
CF	Carbon fibre
cna	Cyanatoaniline
CNSL	Cashew nut shell liquid
CNT	Carbon nanotubes
COLBERT	Catalytic opening of the lateral benzoxazine rings by thiols
cPBO	Crosslinked polybenzoxazole
CPH	Chlorophenol
cPI	Crosslinked polyimide
cpl-dmapa	Caprolactum modified dmapa
CSR	Core shell rubber
dab	1,4-Diaminobutane
dadd	1,12-Diaminododecane
dcbdy	1,4-Dichlorobuta-1,3-diyne
ddbe_p_	Bis-(4-(4-aminophenoxy)phenyl)ether
ddbe_m_	Bis-(3-(4-aminophenoxy)phenyl)ether
dde	4,4′-Diaminodiphenylether
ddm	4,4′-Diaminodiphenylmethane
dds	4,4′-Diaminodiphenylsulfone
deed	*N*, *N′*-diethylethylenediamine
DEP	Diethylphosphite
DGEBA	Diglycidylether of bisphenol A
DHBoP	4,4′-Dihydroxybenzophenone
DHDPE	4,4′-Dihydroxydiphenylether
DHDPS	4,4′-Dihydroxydiphenylsulfone
DHNP	2,7-Dihydroxy naphthalene
dma	Dimethylaniline
dmapa	*N*, *N′*-dimethylaminopropylamine
DMPH	Dimethylphenol
DPA	4,4′-Bis(4-hydroxyphenyl)pentanoic acid
DSC	Differential scanning calorimetry
d*t*bPH	2,4-Di-*tert*-butylphenol
ea	Ethanolamine
ECC	3,4-Epoxycyclohexylmethyl-3,4-epoxycyclohexanecarboxylate
eda	Ethylenediamine
EDP	4,4’-(Ethane-1,1-diyl)diphenol
EMI	2-Ethyl-4-methylimidazole
fa	Furfurylamine
FAD	Furfuraldehyde
FB	Tetrafluoroborate
(Fe)_0_	Iron oxide nanoparticle
(Fe)_TA_	Terephthalic acid coated iron oxide nanoparticle
(Fe)_ATA_	Aminoterephthalic acid coated iron oxide nanoparticle
G	Guaiacol
ha	Hydroxyaniline
hap	Hydroxyacetophenone
HBA	Hydroxybenzoic acid
HBMAC	Hydroxybenzyl methacrylate
HBP	Hydroxyl-ended hyperbranched polyesters
HBAD	Hydroxybenzaldehyde
HBN	Hydroxybenzonitrile
hda	1,6-Hexanediamine
HIn	Phenolphthalein
HCHAL	Dihydroxychalcone
HPAMPH	4-Hydroxy(phenylaminomethyl)phenol
HPBA	Hydroxyphenylbenzamide
HPCBA	2-((2-Hydroxyphenyl)carbamoyl)benzoic acid
HPIO	Hydroxyphenylisoindoline-1,3-dione
HPM	Hydroxyphenylmaleimide
HPNI	*o*-Hydroxyphenylnadimide
HQ	Hydroquinone
HRC	Heat release capacity
IB	Indane bisphenol
LC	Liquid crystal
LSS	Lap shear strength
M	Magnolol
ma	Methylamine
MAF	5-Furfuryl-2,2-dimethyl-[1,3]dioxane-4,6-dione
mba	3-Methylbutan-2-amine
MBM	Phenol- *N*,*N’*-dimethyl-1,3-propanediamine Bz
mda	Methylenedianiline
MeOPH	Hydroxymethylphenol
MeOTs	Methyl-*p*-toluene sulfonate
mepu	4,4′-Methylenebis(3-ethylamine-1-phenylurea)
MMA	Methyl methacrylate
moa	Methoxyaniline
MOF5	[Zn_4_O(BDC)_3_], BDC=1,4-Benzenedicarboxylate
MOPH	Methoxyphenol
MR	2-Methylresorcinol
MSA	Methanesulfonic acid
MU	4-Methylumbelliferone
MWCNT	Multiwalled carbon nanotube
[(nbd)RhCl]_2_	[(Norbornadiene)rhodium(I) chloride]_2_
NH_2_-H(PAM)_2_PH	4-Amino-2-(((4-hydroxy-3-((phenylamino)methyl)phenyl)amino)-methyl)phenol
NH_2_-PAMPH	4-Amino-2-((phenylamino)methyl)phenol
nia	Nitroaniline
NiPH	Nitrophenol
N, N-DBA	*N*, *N′*-dibenzylaniline
NP	Naphthol
Oda	4, 4′-Oxydianiline
*o*MBA	*o*-Methyl bisphenol A
*o*TFHPA	2,2,2-Trifluoro-N-(2-hydroxyphenyl)acetamide
pamam	Polyamindoamine dendrimer
PBA	Phenylboronic acid
PBO	Polybenzoxazole
PBOM	2,6-Diphenylbenzo[1,2-*d*:5,4-*d*’]bis(oxazole)
PBz	Polybenzoxazine
*p*C	*p*-Cresol
*p*C-a-*p*C	Ring-opened *p*C-a Bz with *p*-Cresol
(*p*C-appe)_2_	Dimer of *p*C-appe
PCL	poly(ε-Caprolactone)
pda	Phenylenedianiline
pea	Phenylethanamine
PF	Hexafluorophosphate
PH	Phenol
Ph_2_I^+^PF_6_^-^	Diphenyliodonium hexafluorophosphate
Ph_3_S^+^AsF_6_^-^	Triphenylsulfonium hexafluorophosphate
PG	Pyrogallol
PGU	Phloroglucinol
PGU-NH_2_	1,3,5-Tris(4-aminophenoxy)benzene
PIPF	Diphenyliodonium hexafluorophosphate
PIFA	Diphenyliodonium hexafluoroarsenate
*p*Jeff-V-a	*p*-Jeffamine funcionalized V-a
PN	Phosphazene
pna	Phosphonitrilic amine
POSS	Polyhedral oligomeric silsesquioxane
Pra	*n*-Propylamine
PRB	poly(Resorcinol phenylboronate)
Pyoa	4-(Prop-2-yn-1-yloxy)aniline
PU	Polyurethane
PVPH	Poly(4-vinylphenol)
R	Resorcinol
RES	Resveratrol
ROP	Ring-opening polymerization
SB	Spirobiindane bisphenol
St	Stearate
SP(St-DVB)	Sulfonated poly (styrene-divinylbenzene) microspheres
t	Toluidine
T	1,3,5-Tri(*p*-hydroxyphenyl)benzene
TA	*p*-Toluic acid
*T_5%_*	5% Weight reduction temperature
*T_10%_*	10% Weight reduction temperature
*T*_g_	Glass transition temperature
*T*_o_	Onset temperature of polymerization
*T*_p_	Exothermic peak temperature of polymerization
TBMI	*N*,*N*′-(2,2,4-trimethylhexan-1,6-diyl)bismaleimide
*t*btmpPH	4-(*tert*-Butyl)-2-(2,4,4-trimethylpentan-2-yl)phenol
TCP	Tetrachlorophthalic anhydride
TCP-*p*APH-a	TCP protected *p*APH-a
TDA	3,3′-Thiodipropionic acid
TDPH	4,4′-Thiodiphenol
TEOS	Tetraethoxysilane
tepa	Tetraethylenepentamine
tapm	Tetra-*p*-aminophenylmethane
TFAPH	Trifluoroacetamidophenol
TIPO	Titanium isopropoxide
tma	Trimethylaniline
TMPH	Trimethylphenol
tpa	Triphenylamine
TPHB	Triphenylbenzene
trisapm	*p*-Rosaniline amine
TsOH	*p*-Toluenesulfonic acid
U	Umbelliferone
V	Vanillin
va	Vinylaniline
XY	Xylenol

## Appendix A

**Table A1:** DSC characteristics of representative benzoxazine monomers in presence of aid (catalyst/initiator/co-reactant).

Year	Monomer/Aid	Category	Curing Parameters												Ref.
			*T*_o_	*T*_p_	Δ H(J/g)	*T*_5%_								*T*_10%_	
1995	BA-a ^a^	Monomer	188	226	313	-								-	[266]
	BA-a/adipic acid, sebacic acid, 2,2′-dihydroxybiphenyl (6 mol%)	Brønsted acid	150–154	188–203	352–322	-								-	
1999	2, 4-XY-cha/BF_3_.OEt_2_	Lewis acid	Curing time studied												[257]
1999	*p*C-ma/CF_3_COOH (10 mol%) Sebacic acid (9 mol%) *p*-cresol (10 mol%)	Brønsted acid and phenol	Catalysis studied with curing time												[256]
1999	BA-a	Monomer	-	231	-	-									
	BA-a/MeOTs (5 wt%)	Brønsted acid	~80	144, 179, 200	-	-									[258]
1999	BA-a/PCl_5_ (20:1 mole ratio, 2wt%)	Lewis acid		122, 189, 264	-	-					-				[255,416]
	PCl_3_, POCl_3_, TiCl_4_, AlCl_3_, SbCl_5_/oxetane, MeOTs, MeOTf, CF_3_COOH, BF_3_.OEt_2_, (C_6_H_5_)_3_CSbCl_6_, MeI, BuLi, (C_6_H_5_CO)_2_O_2_	Lewis acids, akylating agent, covalent initiator, anionic initiator, free radical initiator	DSC data not available												
2001	BA-a	Monomer	-	-	~320	-								-	[80]
	Ba-A/PCL (11–100 wt%)	Blend	-	-	~270–88	-								-	
2002	BA-a	Monomer	>360 min												[417]
	BA-a/Oxalic acid (0.07 wt%)	Acid	283 min.		Gelation time at 140 °C										
	*N,N*-dimethylamine (1 wt%)	Amine	104 min.												
	Epoxy resin (10 wt%) *N,N*′- dimethylbenzylamine (1 wt%)	Blends	193 min.												
2003	PH-a/Ph_2_I^+^PF_6_^−^ (0.1–2.4 molL/Ph_2_I^+^PF_6_^−1^)	Onium salts			17–72% % conversion at 300 nm										[299]
	Ph_3_S^+^AsF_6_/Ph_2_I^+^PF_6_^−^ (2.4 molL/Ph_2_I^+^PF_6_^−1^)	Onium salts			63%										
2003	BA-a/Organically modified clay	Nanocomposite	144 ^b^	217	298	-								-	[302]
2004	BA-a/TIPO (10 wt%)?	Nanocomposite	146	220	51 cal/g	350								425	[304]
2005	BA-a	Monomer	220 ^b^	239	-	-								-	[305]
	BA-a/CF (59 wt%)	Nanocomposite	176	214	-	-								-	
2006	BA-a	Monomer	~220 ^b^	~255	-	315								-	[191]
	MBM/BA-a/clay nanocomposite (10 wt%) ^a^	Nanocomposite	~185	225	-	331								-	
2005	BA-a	Monomer	~190 ^b^	~240	-	-								-	[52]
	BA-a/CuCl, CuCl_2_	Transition metalsLewis acid	~160	~223	-	-								-	
2006	BA-a	Monomer	~230 ^b^	~257	309	-								-	[312]
	BA-a/MWCNT (1 wt%)	Nanocomposite	240	248	286	-								-	
2006	BA-a	Monomer	2 h at 200 °C, heating												[301]
	BA-a/SiC (4 wt%)	Microwave radiation at 270 W	<20 min.												
2008	BA-a/Epoxy	Blends	~210 ^b^	~260	-	309									[335]
	BA-a (50 mol%)/Epoxy (50 mol%)/salt of diethanolamine and TsOH (10 wt%)	Latent curing agent	~130 ^b^	210	-	302									
2008	PH-pyoa	Monomer	225	240	772	348								386	[418]
	PH-pyoa/([(nbd)RhCl]_2_) (10 µmol)	Transition metal	188	221	130	330								371	
2008	BEP	Monomer	-	241	-	360								-	[345]
	BEP/CNT:CSR (1:0,0:11, 1:11 wt%)	Nanocomposite	-	233–236	-	360								-	
2010	BA-a	Monomer	-	263	-	-								-	[406]
	BA-a/EMI (5 mol%)	Amine	-	235	-	-								-	
	BA-a/TsOH. H_2_O (5 mol%)	Brønsted acid	-	212	-	-								-	
	BA-a/Mn(acac)_2_, Fe(acac)_3_ (5 mol%)	Lewis acid	-	203–209	-	-								-	
2010	BA-a	Monomer	187	219	271	-								-	[86]
	C-a		273	263	71	-								-	
	C-a/BA-a (38 wt%)	Blending	233	250	114	-								-	
2011	BA-a	Monomer	221	249	-	-								-	[419]
	BA-a/TEOS (39 wt%)	Blending	169	223	-	-								-	
	BA-a/TEOS (39 wt%)/PH-aptes (20 mol%)		191	242	-	-								-	
2011	BA-a	Monomer	~115 ^b^	191	-	283								293	[404]
	BA-a/resorcinol (10 mol%)	Urethane derivatives	~120	177	-	279								290	
	BA-a/resorcinol:phenyl isocyanate (10:10 mol%)		~125	~150	-	275								288	
2011	*p*C-a/TsOH, MeOTs, CHOTs	Alkyl sulfonates	32–95% conversion at 100 °C in 1 day												[290]
2011	BA-a	Monomer	~180 ^b^	237	277	-								-	[274]
	BA-a/CNSL (different ratios)	Phenol	~130–170	197–216	194–246	-								-	
2011	BA-a/BADCY, epoxy	Blend	~200–215 ^b^	~240–260	-	310–330								-	[175]
2012	BA-a	Monomer	~200 ^b^	242	313	-								-	[420]
	BA-a (100)/TDPH (10 wt%)	Blend	~130	218	361	218								-	
	BA-a/DGEBA (different ratios)		~225	258–275	276–326	-								-	
	BA-a/DGEBA/TDPH (different ratios)		~160	228–239	342–348	-								-	
2012	BA-a	Monomer	178 ^b^	227	232	-								-	[421]
	BA-a/Lignin (50 wt%)	Phenol	112	211	64	-								-	
2012	PH-pra/2-methylresorcinol (2:1 mol ratio)	% conversion at RT in methanol at different time interval													[422]
2012	PH-a	Monomer	247	254	298	-								-	[423]
	BA-a		249	261	277	-								-	
	PH-a, BA-a/Lignin (30 wt%)	Polyphenolic materials	175–176	203–212	202–303	-								-	
2012	BA-a	Monomer	240 ^b^	261	-	-								-	[424]
	BA-a/FeCl_3_ (1 wt%)	Lewis Acid	135	~205	-	-								-	
2012	BA-ala	Monomer	150	230, 278	-	334								363	[283]
	(BA-ala)/1,2- ethanedithiol	Blend/Thiol-ene reaction	140, 225	175, 260	-	-								-	
2013	BA-mda	Monomer	~175 ^b^	~250	232	362								384	[347]
	BA-mda/GO (1–5 wt%)	Nanocomposite	~180–~190	~250	224–155	365–366								389	
2013	PH-a	Monomer	0%	% conversion at 60 °C for 2 days											[314]
	PH-a, PH-ma, PH-ba, *p*C-a, BA-a/BF_3_.OEt_2_ (40:1 mol ratio)	Blend	0–98%												
2013	Bz (Epsilon 99100 RTM)	Monomer	214	-	329	-							-		[425]
	Bz (Epsilon 99100RTM)/CNT (0.1–1 wt%)	Nanocomposites	200	-	316–327	-							-		
2013	G-fa	Monomer	219 ^a^	240	62	332							401		[334]
	G-fa/MSA, TsOH, MeOTs (different ratios)	Acids	125–191	173–203	103–115	313–335							407–450		
2012	BEM-80/140/180	Epoxy/amine	~185–~200	227–244	169–204	192–199								243–275	[326]
2013	BA-a	Monomer	203	240	299	240								305	[275]
	BF-a		170	232	311	301								353	
	BA-a, BF-a/TDPH (12 mol%)	Blend with acid	132–172	218–222	300–331	214–252								308–312	
	BA-a, BF-a/TDA (15 mol%)		130–145	196–198	294–388	200–265								246–338	
	TDPH-a		173	221	350	313								334	
	TDPH-a/TDPH (12 mol%)		144	209	316	381								311	
	TDPH-a/TDA (15 mol%)		146	191	315	280								316	
2013	*p*C-a	Monomer	262	269	77 kJ/mol	-					-				[426]
	*p*C-a/(MX, M = Na, Li, NH_4_^+^, Zn^+2^, Cu^+2^, Al^+3^, Fe^+3^ Ag^+^, Co^+2^; X = I, ClO_4_, SCN, Br, OPh, SPh, Cl, OAc, OTf, DMAP, EMI, 2-/3-/4-hydroxypyridine, TsOH.2H_2_O	Catalysts	168–262	197–269	67–88 kJ/mol	-					-				
2013	C-a	Monomer	242	263	71	358					391				[11]
	*p*HBA-a		158	165	177	290					330				
	BA-a		187	217	271	313					342				
	C-a, BA-a: *p*HBA-a (1:0.1)	Binary blends	175–513	220–255	54–262	307–343					374–382				
	C-a:BA-a (1:3–3:1)		211–235	250–281	74–223	287–345	353–389								
	C-a:BA-a: *p*HBA-a (1–3:1–6:0.05–0.3)	Ternary blends	161–201	209–235	200–129	258–346	339–421								
2013	PH-a/BF_3_.OEt_2_ (40/1 wt%)	Cationic initiator	Polymer yield 98% at 60 °C in two days												[314]
2013	BA-a ^a^	Monomer	~200 ^b^	~240	-	-							-		[346]
	BA-a/MWCNT (1:1 wt%)	Nanocomposites	160	230	-	-							-		
	BA-a/MWCNT (1:1 wt%) thermal curing		No exotherm in DSC			-							-		
	BA-a/MWCNT (1:1 wt%) microwave curing					417							-		
2013	BA-a/TBMI (1/1 mol)	Blend	~190 ^b^	247	-	-							-		[427]
	BA-a/TBMI/Imidazole (1/1 mol/3 wt%)	Ternary blend	100	180, 220	-	-		-							
2013	BA-a	Monomer	241	261	149 KJ/mol	-		-							[428]
	BA-a/TBMI (1:1 mol ratio)	Blend	194	247	121	-		-							
2013	BMI/BA-a (2:1 wt ratio)/BADCY (30 wt%)	Ternary blend	150 ^b^	~220	-	333		-							[429]
2013	BA-a	Monomer	-	261	-	297		-							[346]
	BA-a/GO (0.5–3wt%)	Nanocomposites	-	236–250	-	307-ND		-							
	BA-a/Graphite (0.5–3 wt%)		-	264–266	-	300-ND		-							
2014	*p*C-a	At 150 °C for 6 h, without promoter	0%												[278]
	*p*C-a/Thiophenol, *p*-nitrothiophenol (10 mol%)	Acids at 150 °C for 6 h	97–99%												
2014	BA-a	Monomer	-	~250	-	-			-						[430]
	BA-a/PBOM (5–20 wt%)	Composite	-	210–220	-	-			-						
2014	PH-a	Monomer	~250 ^b^	265	-	-			-						[431]
	2,4-XY-tma		-	-	-	-			-						
	BADCY		~290	331	-	-			-						
	PH-a, 2,4-XY-tma/BADCY (1:1 mol%)	Blend	~175 ^b^	225–246	-	-			-						
2015	BA-had ^a^	Monomer	178	255	193	162			271						[432]
	BA-hda/1,2-ethanethiol; 1,6-hexanedithiol (2.4 wt%)	Thiols	165–169	205, 269	155–185	161–258			207–280						
2015	PU/PH-ma (70:30 wt%)	Blends	110 ^b^	191	52	-			-						[367]
	PU-Phenol/PH-ma (70:30 wt%)		140	215	18	-			-						
2015	BA-a	Monomer	213	234	309	293			331						[323]
	BA-a/aromatic and aliphatic diamine	Blend	57–206	80–254	4–70	251–318			266–351						
2015?	BA	Monomer	228	248	325	360				393					[319]
	BA/Phenylboronic acid (5–20 wt%)	Acid	165–209	211–232	230–287	284–330				356–412					
2015	PH-apa	Lewis acid	~175 ^b^	247	879	-				-					[316]
	PH-apa/Ni(acac) (0.01mol)/triphenyl phosphine (0.01mol)		~140 ^b^	184, 228	878	-				-					
2015	BAPBACP	Monomer		240, 341	246, 62	-				-					[317]
	BAPBACP/Fe(acac)_3_ (3.5wt%)	Lewis acids		218, 351	72, 46	-				-					
2016	BA-ala	Monomer	203	266	−316	342				362					[285]
	BA-ala/S_8_ (90 wt%)	Chemical linking	161	182	−11	219				283					
2016	*p*C-*p*t	Monomer	~242 ^b^	273	220	-				-					[286]
	*p*C-*p*t/S_8_ (0.5–5 wt%)	Blend	~170	~227–~250		-				-					
	HQ-a	Monomer	~230	~260	-	-				-					
	HQ-a/S_8_ (0.5–5 wt%)	Blend	~190–210	220–242	-	-				-					
2016	C-a	Monomer	240	263	-	345				393					[310]
	C-a/MOF5 (1–15 wt%)	Lewis acids	178–228	227–251	-	416-ND				429-ND					
2016	C-a/S_8_ (80 wt%)	Chemically linked sulfur	No exotherm and endotherm, cured during reaction			-				-					[21]
2016	T-a	Monomer	208	238	-	396				424					[145]
	T-a/Ph, MR, HQ, PG, *p*MOPH (1.5 eq.)	Blend	88–165	115–237	-	-				-					
2016	BA-a	Monomer	212	245	322	289				323					[294]
	BA-a/BA (5–25%)	Blend	122–180	192–229	333–349	265–297				293–325					
2016	BA-a/amine, NaI, diacid, indole, imidazole, *p*-*tert*-butyl phenol (10 mol%)	Amines, acid, phenol		216–254	-	-				-					[330]
2016	BF-a	Monomer	202	228	254	195				235					[340]
	BF-a/m-xylylenediamine, trimethylhexamethylenediamine (0.011 mol)	Basic catalyst	71–211	131–242	7–47	159–175				201–219					
2016	BA Bz (Huntsman)	Monomer	203 ^b^	243	-	-				-					[300]
	BA Bz/ArIFB (1–3 wt%)	Diaryliodinium salts	166–169	213–225	-	-				-					
	BA Bz/PIPF (1–3 wt%)		160–162	212–218	-	-				-					
	BA Bz/PIFA (1–3 wt%)		164–169	218–226	-	-				-					
2017	BA-a/PH-a/CY-179/High molecular weight phenoxy resin/XS-EP-7 (35:25:12.5:12.5:15 wt%)	Flame Retardant composition Blend	160 ^b^	~220	-	-				-					[433]
2018	PH-a, PH-dmapa, PH-cpl-dmapa	Brønsted acids	160–202	195–238	36–262	310–331				-					[273]
	BA-a, BA-dmapa, BA-cpl-dmapa		148–196	190–247	32–195	260–339				-					
	BF-a, BF-dmapa, BF-cpl-dmapa		122–152	196–225	37–189	193–391				-					
	4,4-BF-a, 4,4-BF-dmapa, 4,4-BF-cpl-dmapa		150–156	186–213	44–147	262–331				-					
2017	PH-*p*t	Monomer	190	224	124	275					305				[336]
	PH-*p*t/NH_3_OHCl, PhNH_3_Cl, PhNHNH_3_Cl, NH_4_Cl, EtNH_3_Cl (5 mol%)	Amine salts	158–173	185–205	74–161	242–261					284–294				
	BA-a	Monomer	211	237	125	-					-				
	BA-a/PhNH_3_OHCl, NH_3_Cl, PhNHNH_3_Cl, NH_4_Cl, EtNH_3_Cl (5 mol%)	Amine salts	170–181	208–214	35–124	-					-				
2017	C-a:BA-a (3:1)	Monomer blend	218	241	40	338					389				[13]
	C-a:BA-a (3:1)/nanoalumina (1–5 wt%)	Nanoparticle	195–212	239–244	46–95	389-ND					412-ND				
	C-a:BA-a (1:3)		211	231	223	291					356				
	C-a:BA-a (1:3)/nanoalumina (1–5 wt%)		191–196	228–231	110–165	285-ND					359-ND				
2017	PH-ddm/2-phenyl-1,3,2-benzenediolborane	Boron based catalyst	197	238	124	186									[320]
2017	Bz-CN	Blend	~160	230, 265	-	-					-				[172]
	Bz-CN/SH-2100 (10–50 wt%)	Thiol	~155–130	~210–183, ~257–248	-	-					-				
2017	PH-fa/MAF (1:1 mol ratio)	Blend	-	-	-	-					-				[296]
2017	BA-a (Huntsman)	Monomer	186	227	324	325					347				[351]
	BA-a/α-ZrP (1–3 wt%)	Nanocomposite	117–158	209–214	348–351	332-ND					355-ND				
2017	BA-a	Monomer	173	235	295	295					332				[434]
	BA-a/*m*-phenylenediamine formaldehyde oligomer	Blend	130	215	23	322					346				
2018	BA-a	Monomer	212	237	201	326					364				[315]
	BA-a/B(C_6_F_5_)_3_ (3–10 mol%)	Lewis acid Blend	125–180	174–217	103–156	294–323					340–347				
	PH-ba	Monomer	242	261	35	257					267				
	PH-ba/B(C_6_F_5_)_3_ (3–10 mol%)	Lewis acid Blend	163–217	215–251	45–130	244–258					263–297				
2018	BA/ECC (50 wt%) (BA-Huntsman)	Blend	~150 ^b^	~255	-	310					-				[352]
	BA/ECC)/HBP-AMIM^+^PF6^−^ (different wt%)		~125–175	~230–255	-	332–345						-			
2018	BA-a	Monomer	186 ^b^	235	391	303						335			[435]
	BA-a/AC (1–5 wt%)	Acid	164–179	224–230	303–376	306–321						338–355			
2018	C-trisapm	Monomer	254	272	108	387						421			[313]
	C-trisapm/Iron nanoparticles	Nanoparticles blend	172–206	218–246	71–89	306–340						365–386			
2018	C-a	Monomer	242	263	63	292						331			[311]
	C-a/Stearic acid; Stearate salts (1–10 wt%)	Lewis acid	169–226	202–247	68–97	351-ND						390-ND			
2018	BA-a (Polaris tech.)	Monomer	~195 ^b^	~230	-	-						-			[297]
	BA-a/ SP(St-DVB) (5–15 wt%)	Nanocomposite	~175–130	~220–205	-	-						-			
2019	V-fa/CS (0–50:0–50 wt%)	Composite	190-ND	204-ND	35.5-ND	-						91-389			[361]
2019	BA-tepa/Amino cellulose	Composite	181–226	224–256	-	-						-			[363]
2019	BA-a/4-nitrophthalonitrile	Composite	-	219	-	346						372			[57]

^a^ = 5 °C/min, ^b^ = 20 °C/min, ^c^ = *T*_i_, ^d^ = kJ/mol, ND = not determined.

**Table A2:** DSC characteristics of representative benzoxazine monomers.

Year	Monomer/Polymer	*T*_o_ (°C)	*T*_p_ (°C)	ΔH (kJ/mol)	*T*_5%_ (°C)	*T*_10%_ (°C)	Ref.
2003	BA, PH-ala	145	207–265	351, 531	343, 348	367, 374	[53]
	*o*ALPH-a	241	263	84	288	356	
2005	BA, PH-fa	~233	241, 247	250, 265	336, 347	382, 391	[130]
2007	MU-*p*t	225	229	292	304	323	[208]
	Dimer-MU-*p*t	187.5	203	205	293	324	
2007	BA, BF-ba	-	213, 249	67, 81	-	-	[140]
	BA, BF-cha	-	237, 245	72,93	-	-	
2008	BA-aee	180	202	75	-	-	[217]
	(BA-aee-aeea)_main chain_	215	250	105	248	278	
	(BA-aeep)_main chain_	210	243	95	288	328	
2008	PH-bampo	~210	~235	-	-	-	[81]
2008	PH, *p*HBA -a		262 ^c^, 180 ^c^	85 ^d^	-	338, 330	[292]
	PH, *p*HBA -*p*aba		204 ^c^, 208 ^c^	-	-	282, 265	
2008	*p*CPH, *p*MeOPH, *p*HBAD, *p*NiPH, *p*C, *p*MOPH, HQ, PH-a	-	175 ^a^–273 ^a^	77 ^d^–108 ^d^	-	142–490	[138]
	PH-*p*ha, *p*moa, *p*nia, *p*ca	-	195 ^a^–289 ^a^	73 ^d^–107 ^d^	-	335–375	
	*p*MOPH-*p*moa, *p*ca	-	254 ^a^, 274 ^a^	72 ^d^, 73 ^d^	-	315, 302	
	*p*CPH-*p*ca, *p*moa	-	269 ^a^, 257 ^a^	80 ^d^, 76 ^d^	-	300, 321	
2009	*p*C-appe	191	235	-	339	356	[380]
	(*p*C-appe)_2_	140	215	340^d^	349	368	
2009	(BAdcbdy)_n_	-	185, 260	-	-	-	[413]
	BA-appe	-	241	-	-	-	
	Poly(BA-appe)	125	185	-	367	-	
2009	PH-eda	185	185	444	291	-	[137]
	*p*C-dab	193	193	316	251	-	
	*o*C-eda	234	234	317	258	-	
	*m*C-eda	197	197	321	273	-	
	PH-hda		225	292	280	-	
	*p*C-hda		244	253	283	-	
	*o*C-hda		250	250	277	-	
	*m*C-hda		238	261	272	-	
	PH, *p*C, *o*C, *m*C- dadd	241–262	-	215–229	281–290	-	
2009	d*t*bPH, *t*btmpPH-pea	-	263 ^b^–294 ^b^	5.42–81.8	-	-	[160]
2010	poly(PH-a-FAD)_main chain_	198	241	-	-	-	[232]
2010	*p*APH-a, ddm	-	96–243	-	-	-	[322]
	TCP-*p*APH-a, ddm	184, 236	210, 237	-	-	-	
	*p*bHPIPA-a	194	223	-	341	380	
2010	C-a:BA-a (0–3:0–3)	187–242	219–263	71–271	-	-	[86]
2011	BHPF-fa	205	252	-	402	422	[154]
2011	BA, BF, DHDPE, EDP, DHBoP, DHDPS -a	206–261	215–267	157 ^d^–144 ^d^	-	-	[400]
2011	*p*HBMAC-a	193	203	315	-	-	[228]
2011	*p*HBA-a	162 ^a,b^	196	64 ^d^	-	-	[375]
2012	C-ddbe_p_, ddbe_m_, dds, ddm, a	195–245	233–267	71–271	292–355	350–389	[399]
2012	PH-0G, 1Gpamam	~177 ^a^,150 ^a^	~273, 271	-	265, 222	314, 304	[143]
2012	PH, *o*MeOPH, *m*MeOPH, *p*MeOPH-ddm	140–224	188–262	211–326	364–394	390–430	[398]
	P-oda	134–227	162–253	262–305	-	-	
2012	HPM-a	169^c^	205, 245	172	344	377	[389]
	HPM-2apa	196^c^	209	568	412	432	
2012	PH, *p*HPBA, *o*HPBA-a	-	187–263	-	-	-	[193]
	(*o*bHPIPA, *p*bHPIPA-ddm)_main chain_	-	212, 259	-	-	-	
2012	PH, *o*HBA, *m*HBA, *p*HBA-a	151–237	196–255	201–336	-	-	[403]
2012	DHBoP-deed	142	174	-	-	-	[237]
2013	BA, Hln-*p*aba	153 ^a^, 166 ^a^	227, 238	-	339, 430	408, 506	[293]
2013	BA-(R,S), (rac)mba	~183 ^b^, ~178 ^b^	223–238	114, 107	297, 298	329, 331	[159]
2013	PH-DEP-a	~ 190	210	83 ^d^	-	-	[17]
2013	PH, *o*C, *m*C, *p*C, DMPH, TMPH-bapf	253 ^c^–279 ^c^	266–301	98–226	322–401	353–432	[157]
2014	4,4′, 2,4′, 2,2′-BF-a	213–255	250–260	199–297	264–353	341–403	[152]
2014	PH, *p*C-amp	~200, 150	240, 208	-	306, 398	350, 475	[376]
2014	V-a	~200 ^c^	231	143	-	-	[97]
	*p*Jeff-V-a	~240 ^c^	256	-	-	-	
2014	PH-pna (26.6–75.2%)	97–171	202–222	-	375–427	407–474	[148]
2014	R-a	~150 ^c^, ~200	179, 229	-	267	-	[402]
2014	*p*CbHPCBA-ddm	~190 ^c^	~220	-	407	486	[242]
2014	*o*APH, TCP-*o*APH-a	~100 ^c^, 194 ^c^	149–222	-	-	-	[241]
	*o*APH, TCP-*o*APH-ddm	~110 ^c^, ~225	151–248	-	-	-	
	HPCBA-a	~150 ^c^	210	-	-	-	
	*o*CbHPCBA-ddm	~190 ^c^	223		511	567	
2015^c^	BA-a	129 ^c^	215	213	-	-	[401]
	IB-a	137 ^c^	214	143	-	-	
	SB-a	231 ^c^	259	134	-	-	
2015	C-a, -ddm/- trisapm/- tapm	140–225 ^c^	190–265	-	355–391	-	[87]
2015	*o*APH-hda, dds	185, 223	238, 281	-	242, 370	-	[206]
2015	EDA-*p*HBAD-a/-*o*ma, -ap	205 ^c^–236	229–252	-	128–395	287–420	[388]
2015	BHPPA, BHPICA, BHPPIO-a	~160 ^c^–~240 ^c^	209–256	92–248	507–536	559–589	[197]
2015	*o*-HPNI-a, ddm	~210 ^c^, ~210 ^c^	231, 245	155, 173	ND, 463	ND, 484	[196]
2016	PH, MU, U-a	215–255	220–261		288–320	343–361	[210]
2016	HPAMPH-a	236	251	359	350	373	[147]
	HPAMPH-NH_2_-PAMPH	224	242	311	353	377	
	HPAMPH-NH_2_-H(PAM)_2_PH	215	237	285	343	366	
2016	MU-a ^a^	~200 ^c^,	229	294	-	-	[211]
	MU-BA-NH_2_, MU-PGU-NH_2_	~220 ^c^	251, 241	90, 146	-	-	
2016	A-fa	150 ^c^	207	220	-	-	[110]
2016	PH, R, PGU-a		172 ^a^–234 ^a^		~214–237	~241–281	[144]
2016	C-ATPEG-200–1500	152–172	220–242	41–94	-	-	[238]
2016	C-BAEPA, AEPA	114 ^c^, 130	215, 238	107, 35	298, 380	-	[116]
2016	1-NP-*p*cna, apacn	~160 ^c^, ~130 ^c^	172–206	565, 603	332, 302	359, 323	[182]
	2-NP-*p*cna, *o*cna	~180 ^c^, ~140 ^c^	174–215	537, 623	311, 309	330, 327	
2016	2-NP-3-apd	171 ^a^	183	167	-	-	[333]
2016	*p*NiPH-*p*Nia, *m*Nia, *p*aap	~190 ^c^, ~210 ^c^	249–286	-	-	-	[386]
	*p*HBA, *p*hap-*p*Nia	~150 ^c^, ~200 ^c^	344, 237	-	-	-	
2017	C-mepu	103	202	-	322	369	[225]
2017	BHPe-a	~245 ^c^	265	23	408	-	[213]
2017	BHPr-a	~190 ^c^	220	257	431	-	[212]
2017	*o*TFHPA-ddm	~170 ^c^	207	-	471	512	[341]
2018	*p*HBN-oda	156	227	184			[170]
2018	BAPh	-	-	-			[171]
	BA, *N, N*-DBA, *p*C-a-*p*C/BAPh	241–304	265–>350	31–103	413–479	451–521	
	BA-appn	~175 ^c^	220, 260	-	456	511	
2018	C-*p*ha, -*p*aba, -a	~140 ^c^–~200 ^c^	186–255	-	-	292–302	[436]
2018	*o*bHPIITO-a, -oda	~150 ^c^, ~250 ^c^	234, 314	123, 58	-	400, 460	[244]
2018	*p*HBA-a	201	216	-	395	436	[353]
2018	HCHAL-a	~120 ^c^	206	-	335	375	[214]
2018	BAM-3–9	217 ^c^–230 ^c^	225–243	234–266	-	-	[437]
2018	PH-a	202	238	262	331	-	[273]
	PH-dmapa, cpl-dmapa	170, 160	205, 195	13, 36	323, 310	-	
	BA-a, dmapa, cpl-dmapa	148–196	190–247	32–195	260–339	-	
	4,4′-BF-a + 2,4′-BF-a	152	225	189	391	-	
	4,4′-BF-dmapa + 2,4′-BF-dmapa	135	219	169	249	-	
	4,4′-BF-cpl-dmapa + 2,4′-BF-cpl-dmapa	122	196	37	193	-	
	4,4′-BF-a	150	213	147	331		
	4,4′-BF-dmapa	156	197	68	262	-	
	4,4′-BF-cpl-dmapa	152	186	44	278	-	
2018	SBA-a	170 ^c^	231	180		~60	[192]
2018	*o*HPNI-a	~230 ^c^	245	173	371	431	[245]
	Poly(*o*HPNI-a)_main chain_	~225 ^c^	241	120	319	366	
2018	PG-fa, a	152, 166	177, 189	-	-	-	[408]
2019	β-NP, G, C-ima	238–246	253–266	81–183	-	318–358	[89]
2019	M-fa	-	229	-	440	463	[105]
2019	RES-fa	193	229	324	346	403	[106]
2019	HPM-fa	-	214	-	350	403	[215]
	Poly(HPM-fa)_main chain_	-	217	-	336	370	

^a^ = 5 °C/min, ^b^ = 20 °C/min, ^c^ = *T*_i_, ^d^ = kJ/mol, ND = not determined.
